# Conversational AI-Enabled Precision Oncology Reveals Context-Dependent MAPK Pathway Alterations in Hispanic/Latino and Non-Hispanic White Colorectal Cancer Stratified by Age and FOLFOX Exposure

**DOI:** 10.3390/cancers18020293

**Published:** 2026-01-17

**Authors:** Fernando C. Diaz, Brigette Waldrup, Francisco G. Carranza, Sophia Manjarrez, Enrique Velazquez-Villarreal

**Affiliations:** 1 Lineberger Comprehensive Cancer Center, University of North Carolina, Chapel Hill, NC 27514, USA; 2 Department of Integrative Translational Sciences, Beckman Research Institute, City of Hope, Duarte, CA 91010, USA; 3 City of Hope Comprehensive Cancer Center, Duarte, CA 91010, USA

**Keywords:** colorectal cancer, early-onset colorectal cancer, MAPK signaling pathway, cancer disparities, cancer genetics, precision oncology, FOLFOX chemotherapy, artificial intelligence, AI-agents, conversational AI

## Abstract

Background: Colorectal cancer (CRC) demonstrates substantial clinical and biological diversity across age groups, ancestral backgrounds, and treatment settings, alongside a rising incidence of early-onset disease (EOCRC). The mitogen-activated protein kinase (MAPK) pathway is a major driver of CRC development and therapy response; however, the distribution and prognostic value of MAPK alterations across distinct patient subgroups remain unclear. Methods: We analyzed 2515 CRC tumors with harmonized demographic, clinical, genomic, and treatment metadata. Patients were stratified by ancestry (Hispanic/Latino [H/L] vs. non-Hispanic White [NHW]), age at diagnosis (early-onset [EO] vs. late-onset [LO]), and FOLFOX chemotherapy exposure. MAPK pathway alterations were identified using a curated gene set encompassing canonical EGFR-RAS-RAF-MEK-ERK signaling components and regulatory nodes. Conversational artificial intelligence (AI-HOPE and AI-HOPE-MAPK) enabled natural language-driven cohort construction and exploratory analytics; findings were validated using Fisher’s exact testing, chi-square analyses, and Kaplan–Meier survival estimates. Results: MAPK pathway disruption demonstrated marked heterogeneity across ancestry and treatment contexts. Among EO H/L patients, *FGFR3, NF1, and RPS6KA6* mutations were significantly enriched in tumors not receiving FOLFOX, whereas *PDGFRB* alterations were more frequent in FOLFOX-treated EO H/L tumors relative to EO NHW counterparts. In late-onset H/L disease, *NTRK2* and *PDGFRB* mutations were more common in non-FOLFOX tumors. Distinct MAPK-associated alterations were also observed among NHW patients, particularly in non-FOLFOX settings, including *AKT3, FGF4, RRAS2, CRKL, DUSP4, JUN, MAPK1, RRAS,* and *SOS1*. Survival analyses provided borderline evidence that MAPK alterations may be linked to improved overall survival in treated EO NHW patients. Conversational AI markedly accelerated analytic throughput and multi-parameter discovery. Conclusions: Although MAPK alterations are pervasive in CRC, their distribution varies meaningfully by ancestry, age, and treatment exposure. These findings highlight *NF1, MAPK3, RPS6KA4*, and *PDGFRB* as potential biomarkers in EOCRC and H/L patients, supporting the need for ancestry-aware precision oncology approaches.

## Simple Summary

Colorectal cancer is not the same for every patient; its behavior can change depending on a person’s age, ancestry, and the treatments they receive. In this study, we examined tumors from more than 2500 people with colorectal cancer to better understand changes in the MAPK signaling pathway, a major driver of tumor growth and treatment resistance. We found that MAPK-related gene mutations were extremely common, but the specific genes affected differed across groups. For example, some MAPK alterations were more frequent in early-onset cancers diagnosed in Hispanic/Latino patients, especially when tumors had not been treated with FOLFOX chemotherapy. Other gene changes were more common in older individuals or in non-Hispanic White patients. When we looked at survival, we saw only small differences linked to MAPK alterations, suggesting that pathway changes alone may not determine outcomes. We used a conversational artificial intelligence system to rapidly compare patient groups and uncover patterns that might otherwise be missed. These findings show that personalized colorectal cancer care will benefit from considering age, ancestry, and treatment history, not just whether MAPK genes are altered.

## 1. Introduction

Colorectal cancer (CRC) remains a leading cause of cancer morbidity and mortality worldwide, yet its clinical course is increasingly heterogeneous across age groups, tumor biology, and population backgrounds [1,2]. A particularly concerning shift is the continued rise of early-onset colorectal cancer (EOCRC), which reinforces the need for biomarker strategies that are both precision-oriented and equity-aware, especially for populations that have been historically underrepresented in genomic research and clinical trial datasets [1,2,3]. Despite expanding access to next-generation sequencing and targeted therapies, the molecular determinants that shape treatment response and outcomes across clinically relevant subgroups remain incompletely defined [1,3,4].

A central axis in CRC pathobiology is the EGFR-RAS-RAF-MEK-ERK (MAPK) signaling cascade, which regulates proliferation, survival, invasion, and therapy resistance [5,6]. MAPK biology is tightly linked to the evolution of personalized CRC care: anti-EGFR monoclonal antibodies (cetuximab and panitumumab) represented an early breakthrough in targeted therapy, and biomarker-driven selection, particularly expanded RAS testing and the recognition of BRAF-driven disease biology, has improved patient stratification and reduced ineffective treatment exposure [4,7,8,9,10,11]. However, even within biomarker-selected populations, response to anti-EGFR therapy remains variable, highlighting the need for additional predictive biomarkers and a deeper mechanistic understanding of resistance beyond canonical RAS and BRAF alterations [7,9,11,12].

The MAPK pathway is also implicated in chemoresistance to CRC standard-of-care regimens, including oxaliplatin- and fluoropyrimidine-based therapies such as FOLFOX [13,14,15]. Mechanistic studies have shown that MAPK/ERK activation can promote epithelial–mesenchymal transition (EMT) and oxaliplatin resistance [16], while p38 MAPK signaling can facilitate survival programs in oxaliplatin-resistant CRC models and contribute to escape from apoptosis [13,17]. These findings underscore that MAPK activity is not merely a downstream readout of oncogenic drivers; it is dynamically shaped by therapeutic pressures and pathway cross-talk [14,15]. For example, inhibition of mTORC1 can trigger compensatory MAPK activation through a PI3K-dependent feedback loop, supporting the rationale for combination strategies that anticipate adaptive signaling [18]. Complementing these mechanistic insights, clinical correlative studies have linked MAPK-associated epigenetic regulation and signaling states to outcomes in patients treated with FOLFOX-based therapy, suggesting potential biomarker roles that are context dependent [19].

Meanwhile, therapeutic targeting of the MAPK pathway in CRC has advanced but remains challenging [20,21,22,23,24]. Although RAF and MEK inhibitors have transformed outcomes in several RAF-driven malignancies, CRC displays complex intrinsic and adaptive resistance mechanisms that can limit the durability of response [25,26,27,28,29,30]. Contemporary CRC precision oncology emphasizes that effective MAPK-directed strategies will require improved molecular stratification, rational combinations, and biomarker discovery beyond single-gene predictors [7,11,31,32]. These challenges are further complicated in real-world settings, where treatment patterns vary (e.g., FOLFOX, CAPEOX, FOLFOXIRI) and where subgroup-specific evidence, particularly in underrepresented populations, remains limited [31,33,34,35].

A practical barrier to progress is not only biological complexity, but also analytic complexity: actionable insights often require integrating age of onset, treatment exposure, clinicopathologic features, and ancestry or population descriptors with high-dimensional genomic features [1,2,3]. Traditional analytic workflows are rigorous but can be slow to iterate when investigators need to test multi-parameter hypotheses across stratified cohorts. This limitation is especially relevant for disparity-focused precision oncology, where subgroup-aware analyses are essential and sample sizes can be uneven across strata.

To address these gaps, we apply a conversational artificial intelligence (AI) framework, AI-HOPE [36] and its pathway-specific module, AI-HOPE-MAPK [37], to accelerate clinical-genomic integration and hypothesis testing through natural language-driven queries. In this study, we characterize MAPK pathway alterations across 2515 CRC cases, stratified by ancestry (H/L vs. NHW), age of onset (early vs. late), and FOLFOX exposure, and we assess associations with survival outcomes [38,39,40]. By combining conventional statistical testing with AI-enabled cohort interrogation, we aim to clarify how MAPK alterations vary by clinical context and treatment status, and to establish an efficient, reproducible approach for biomarker discovery that supports precision medicine for populations historically underrepresented in cancer genomics research.

## 2. Materials and Methods

### 2.1. Study Design and Data Provenance

This study employed a retrospective, integrative analysis of de-identified colorectal cancer (CRC) cases aggregated from large-scale, publicly available cancer genomics resources. Clinical, genomic, demographic, and treatment-level data were obtained from harmonized datasets hosted on cBioPortal, including The Cancer Genome Atlas (TCGA) Colorectal Adenocarcinoma (PanCancer Atlas), MSK-CHORD (2024), and the AACR Project GENIE Biopharma Collaborative (BPC) CRC cohort. These datasets were selected for their depth of somatic variant annotation, availability of longitudinal clinical metadata, and representation of real-world treatment exposure.

Eligible cases included patients with histopathologically confirmed colorectal adenocarcinoma and available tumor-based next-generation sequencing data. When multiple sequencing records were present for a single individual, one representative sample was retained to prevent duplicate inclusion.

### 2.2. Population Stratification and Clinical Subgroup Definitions

Patients were stratified by ancestry, age at diagnosis, and chemotherapy exposure. Hispanic/Latino (H/L) ancestry was determined using self-reported ethnicity fields when available. In datasets lacking explicit ethnicity annotation, probabilistic surname-based inference methods were applied to identify likely H/L ancestry. The comparator population consisted of non-Hispanic White (NHW) patients meeting identical inclusion criteria.

EOCRC was defined as a diagnosis prior to 50 years of age, while late-onset colorectal cancer (LOCRC) was defined as a diagnosis at age 50 years or older. These definitions were applied uniformly across all datasets to ensure consistency.

### 2.3. Treatment Exposure Annotation and FOLFOX Classification

Chemotherapy exposure was abstracted from structured treatment variables and curated medication records. Patients were classified as FOLFOX-treated if documented exposure included all three regimen components (5-fluorouracil, leucovorin, and oxaliplatin) administered within the same therapeutic line or within an overlapping treatment window consistent with standard FOLFOX protocols.

Patients lacking documentation of all three agents, or who received partial or alternative regimens (e.g., single-agent fluoropyrimidine, oxaliplatin alone, or non-overlapping administration), were categorized as non-FOLFOX. Treatment timing relative to sequencing was reviewed to confirm relevance to tumor molecular profiles.

### 2.4. MAPK Pathway Curation and Genomic Alteration Assessment

A curated MAPK signaling gene set was assembled by integrating canonical pathway definitions from KEGG and Reactome with contemporary CRC-focused literature describing MAPK-driven tumorigenesis, therapy resistance, and pathway cross-talk. This gene set encompassed upstream receptor tyrosine kinases, core RAS/RAF/MEK/ERK components, MAPK effector kinases, and regulatory feedback nodes.

Somatic mutation data were extracted from mutation annotation format (MAF) files and equivalent variant tables. Only protein-altering variants, including missense, nonsense, frameshift, splice-site, and start-loss mutations, were retained for analysis. MAPK pathway alteration status was defined at the patient level as the presence of at least one qualifying somatic alteration in any MAPK-associated gene.

### 2.5. Statistical Analyses

Comparisons of MAPK alteration frequencies across ancestry, age-of-onset, and treatment-defined subgroups were performed using Fisher’s exact test or chi-square test, as appropriate based on cell counts. Continuous clinical variables were evaluated using non-parametric tests.

Overall survival (OS) was defined as the interval from diagnosis or sequencing (depending on dataset availability) to death or last follow-up. Survival distributions were estimated using Kaplan–Meier methods, and group-level differences were assessed using the log-rank test. Exploratory Cox proportional hazards models were constructed to estimate hazard ratios with 95% confidence intervals, adjusting for available covariates including age, sex, tumor site, microsatellite instability (MSI) status, and chemotherapy exposure. All statistical analyses were conducted using R (version 4.3), with two-sided *p*-values < 0.05 considered statistically significant.

### 2.6. Conversational AI-Enabled Integration and Analytic Workflow

To complement traditional statistical workflows, we implemented AI-HOPE and its pathway-specific module AI-HOPE-MAPK, conversational artificial intelligence platforms designed to integrate clinical, genomic, demographic, and treatment variables within a unified analytic environment. These systems enable natural language-driven cohort construction, pathway-specific mutation querying, and rapid subgroup stratification across high-dimensional datasets.

AI-HOPE-MAPK was used to identify patients meeting compound criteria combining ancestry, age of onset, treatment exposure, and MAPK alteration status; generate pathway-level mutation frequency summaries stratified by clinical and demographic variables; perform iterative subgroup exploration to prioritize candidate ancestry- and treatment-associated MAPK alterations; and facilitate reproducible analytic logic through transparent, query-based workflows.

Conversational artificial intelligence tools (AI-HOPE and AI-HOPE-MAPK) were used as an orchestration and acceleration layer to facilitate cohort construction, subgroup stratification, and hypothesis generation across high-dimensional clinical and genomic data. The AI interface enabled natural-language queries to define inclusion criteria (ancestry, age at diagnosis, treatment exposure, and gene alteration status) and to rapidly generate candidate sub-cohorts. Importantly, all AI-generated cohorts and intermediate outputs were independently reconstructed using traditional rule-based filtering and verified for concordance in cohort size and composition. All statistical analyses, including mutation frequency comparisons (Fisher’s exact or chi-square tests) and survival analyses (Kaplan–Meier estimation and Cox proportional hazards modeling), were performed using standard, non-AI statistical methods. Only results that were fully concordant between AI-assisted and conventional analyses were reported.

All AI-assisted outputs were cross-validated against conventional data extracts and independently confirmed using standard statistical analyses. The AI platform was used as an analytic accelerator rather than a replacement for statistical inference, ensuring reproducibility, interpretability, and methodological transparency.

## 3. Results

### 3.1. Cohort Composition and Baseline Clinical Characteristics

Baseline demographic, clinical, and molecular features of the study population are detailed in Table 1. The final analytic cohort comprised 2515 colorectal cancer (CRC) cases, including 266 H/L (10.6%) and 2249 NHW (89.4%) patients. Distinct distributions were observed across age-of-onset and treatment-defined subgroups, underscoring population-specific differences in clinical presentation and therapeutic exposure.

When stratified by age at diagnosis and FOLFOX exposure, H/L patients showed a greater proportional burden of early-onset disease. Specifically, EOCRC treated with FOLFOX accounted for 27.4% of H/L cases compared with 16.7% among NHW patients, while EOCRC not exposed to FOLFOX was also more frequent in the H/L cohort (19.5% vs. 13.4%). In contrast, NHW patients were more commonly represented among late-onset CRC (LOCRC) receiving FOLFOX (40.9% vs. 34.2%) and LOCRC not treated with FOLFOX (29.0% vs. 18.8%), reflecting differential treatment patterns by age and ancestry.

Tumor anatomic distributions were similar across groups. Colon adenocarcinoma was the predominant diagnosis in both populations (61.7% H/L; 59.0% NHW), followed by rectal adenocarcinoma and colorectal adenocarcinoma not otherwise specified, with only modest proportional variation between cohorts. Sex distributions were likewise comparable, with males representing 59.4% of H/L and 56.3% of NHW cases. All tumors included in the analysis were derived from primary tumor specimens.

Clinical stage at diagnosis demonstrated broadly aligned patterns, although NHW patients exhibited a slightly higher proportion of stage IV disease at presentation (44.7%) compared with H/L patients (40.6%). Microsatellite status differed between groups, with microsatellite-stable (MSS) tumors more prevalent in the NHW cohort (86.3%) than in the H/L cohort (75.2%), while MSI-high tumors comprised a smaller fraction of both populations.

Ethnicity annotations reflected the cohort construction strategy. The H/L group was primarily composed of patients categorized as Spanish/Hispanic NOS (86.5%), with additional representation from Mexican/Chicano (11.3%) and other Hispanic subcategories. As expected, all individuals in the NHW cohort were annotated as non-Hispanic White. Together, these baseline characteristics provide the clinical context for subsequent analyses of ancestry-, age-, and treatment-associated MAPK pathway alterations.

### 3.2. Clinical and Genomic Stratification

#### 3.2.1. Hispanic/Latino CRC by Age of Onset and FOLFOX Exposure

To further dissect ancestry-specific heterogeneity within the H/L cohort, we performed a stratified analysis comparing EOCRC and LOCRC patients according to FOLFOX treatment status (Table 2a). This analysis integrated clinical metrics with MAPK-related genomic features to evaluate how age and chemotherapy exposure shape tumor biology within this disproportionately affected population.

Across both age strata, median age at diagnosis differed modestly by treatment status. Among EO H/L patients, those treated with FOLFOX were diagnosed slightly later than their non-FOLFOX-treated counterparts (median 42 vs. 40 years), approaching statistical significance (*p* = 0.054). In contrast, a significant age difference was observed in LO H/L patients, with FOLFOX-treated individuals diagnosed at a younger median age compared with those not receiving FOLFOX (59 vs. 62 years; *p* = 0.049), suggesting potential age-related treatment selection effects in older patients.

Global measures of TMB showed nuanced differences by age and treatment. Median mutation counts were comparable between EO H/L patients regardless of FOLFOX exposure, while LO H/L patients exhibited similar mutation burdens across treatment groups. TMB demonstrated greater variability in LOCRC: LO H/L patients not treated with FOLFOX had a significantly higher median TMB compared with those receiving FOLFOX (6.9 vs. 6.1 mutations/Mb; *p* = 0.044). In contrast, TMB differences among EO H/L patients did not reach statistical significance. The fraction of genome altered (FGA) did not differ meaningfully by treatment status in either age group.

At the gene-specific level, several MAPK pathway alterations exhibited strong age- and treatment-dependent patterns. In EO H/L patients, *FGFR2* mutations were observed exclusively in non-FOLFOX-treated tumors (7.7% vs. 0.0%; *p* = 0.028). Similarly, *NF1* alterations were significantly enriched among EO H/L patients not treated with FOLFOX (19.2%) compared with those receiving FOLFOX (4.1%; *p* = 0.014). *RPS6KA4* mutations, another MAPK effector, followed a similar pattern, occurring only in EO H/L tumors not exposed to FOLFOX (9.6% vs. 0.0%; *p* = 0.011).

Distinct trends emerged in late-onset disease. While *FGFR2* and *NF1* mutations were infrequent and not significantly different by treatment status in LO H/L patients, *NTRK2* and *PDGFRB* alterations were significantly enriched in LO H/L tumors not treated with FOLFOX (each 6.0% vs. 0.0%; *p* = 0.043). These findings suggest divergent MAPK pathway dependencies between EOCRC and LOCRC within the same ancestral group.

These results demonstrate that MAPK pathway alterations in H/L CRC are strongly modulated by both age of onset and chemotherapy exposure, with distinct gene-level enrichment patterns in early- versus late-onset disease. This stratified genomic landscape underscores the importance of age- and treatment-aware analyses when evaluating precision oncology biomarkers in underrepresented populations and provides a foundation for subsequent ancestry-comparative and survival analyses.

#### 3.2.2. Age-, Treatment-, and MAPK-Specific Genomic Patterns in Non-Hispanic White Patients

To delineate MAPK-associated clinical-genomic heterogeneity within the NHW cohort, we examined EOCRC and LOCRC cases stratified by FOLFOX exposure (Table 2b). This analysis integrated age at diagnosis, global mutational metrics, and gene-level MAPK alterations to assess how treatment context intersects with tumor biology in NHW patients.

Among EOCRC cases, median age at diagnosis was comparable between FOLFOX-treated and non-FOLFOX-treated patients (43 vs. 44 years; *p* = 0.56), indicating minimal age-based selection for chemotherapy in younger NHW individuals. In contrast, LOCRC patients demonstrated a pronounced age difference by treatment status: those receiving FOLFOX were diagnosed significantly earlier than their non-treated counterparts (median 63 vs. 66 years; *p* = 4.1 × 10^−7^), suggesting that age may influence chemotherapy utilization in older NHW populations.

Global mutational measures revealed limited differences in EOCRC but clear treatment-associated divergence in LOCRC. Median mutation counts and TMB were similar between treatment groups in EOCRC. However, among LOCRC patients, non-FOLFOX-treated tumors exhibited significantly higher median mutation counts (8 vs. 7; *p* = 1.2 × 10^−5^) and elevated TMB (6.6 vs. 6.1 mutations/Mb; *p* = 2.9 × 10^−4^) compared with FOLFOX-treated tumors. The fraction of genome altered (FGA) did not differ significantly by treatment status in either age group.

At the gene-specific level, multiple MAPK pathway components showed age- and treatment-dependent enrichment patterns. In EOCRC, *AKT3, FGF4, JUN*, and *RRAS2* mutations were significantly more frequent in tumors not treated with FOLFOX, whereas JUN alterations were enriched in FOLFOX-treated EOCRC tumors (3.2% vs. 0.7%; *p* = 0.027), suggesting distinct MAPK signaling contexts associated with chemotherapy exposure in younger patients.

In LOCRC, treatment-associated differences were more prominent across several MAPK-related genes. Tumors not exposed to FOLFOX demonstrated significantly higher frequencies of *CRKL, DUSP4, MAPK1, RRAS*, and *SOS1* mutations, while *JUN* alterations were again enriched in FOLFOX-treated tumors (1.4% vs. 3.2% in non-treated; *p* = 0.025). These patterns indicate that MAPK pathway perturbations in NHW CRC are not uniform but vary substantially with age and chemotherapy exposure.

These findings reveal that, within the NHW population, MAPK pathway alterations are strongly modulated by age of onset and FOLFOX treatment status, with relatively modest differences in EOCRC and more pronounced genomic divergence in LOCRC. This stratified landscape highlights the importance of incorporating treatment context into MAPK-focused biomarker analyses and provides a framework for subsequent ancestry-comparative and outcome-oriented investigations.

#### 3.2.3. Ancestry-Specific Genomic Features in Early-Onset Colorectal Cancer by Treatment Context

To directly compare ancestry-associated molecular differences in EOCRC, we evaluated H/L and NHW patients stratified by FOLFOX treatment status (Table 2c). This focused analysis allowed assessment of whether clinical and MAPK-related genomic features diverge by ethnicity within the same age-of-onset category.

Among FOLFOX-treated EOCRC cases, median age at diagnosis was similar between H/L and NHW patients (42 vs. 43 years; *p* = 0.085), indicating comparable age distributions at treatment initiation. Measures of global tumor complexity, including mutation count, TMB, and fraction of genome altered (FGA), were also broadly comparable between ancestries in this treatment group. However, gene-level differences within the MAPK pathway were evident. PDGFRB mutations were significantly more frequent in FOLFOX-treated H/L EOCRC tumors compared with NHW counterparts (5.5% vs. 0.8%; *p* = 0.015), suggesting ancestry-associated differences in receptor tyrosine kinase signaling among treated early-onset cases.

In contrast, more pronounced ancestry-related divergence was observed among EOCRC patients not treated with FOLFOX. H/L patients in this group were diagnosed at a significantly younger median age than NHW patients (40 vs. 44 years; *p* = 6.0 × 10^−4^), highlighting a substantial age disparity within untreated early-onset disease. Despite similar overall mutation counts, specific MAPK pathway alterations were markedly enriched in H/L tumors. *MAPK3* mutations were significantly more common in H/L EOCRC not exposed to FOLFOX compared with NHW tumors (5.8% vs. 1.0%; *p* = 0.043). Likewise, NF1 alterations were strongly enriched in H/L patients relative to NHW patients (19.2% vs. 6.0%; *p* = 0.0027), underscoring ancestry-associated differences in negative regulation of MAPK signaling. *RPS6KA4* mutations, a downstream MAPK effector, were also more frequent in H/L EOCRC not treated with FOLFOX (9.6% vs. 2.6%; *p* = 0.039).

These results demonstrate that ancestry-related differences in MAPK pathway alterations are most pronounced in EOCRC patients not exposed to FOLFOX, whereas treated EOCRC shows more limited divergence. The enrichment of *MAPK3, NF1, RPS6KA4*, and *PDGFRB* alterations in H/L patients suggests distinct MAPK signaling dependencies in early-onset disease that may be masked or modified by chemotherapy exposure. These results further support the need for ancestry- and treatment-aware molecular stratification in EOCRC precision oncology.

### 3.3. Distribution of MAPK Pathway Alterations Across Age, Ancestry, and Chemotherapy Context

To determine whether MAPK pathway alteration prevalence varies by age of onset, ancestry, or FOLFOX exposure, we compared pathway-level alteration frequencies across stratified colorectal cancer subgroups (Table 3a–d). In contrast to the gene-specific heterogeneity observed in earlier analyses, MAPK pathway alterations were highly prevalent across nearly all clinical strata, with only modest variation by treatment or ancestry.

#### 3.3.1. Age- and Treatment-Specific Patterns Within Ancestry Groups

Within the H/L cohort, MAPK pathway alterations were detected in the vast majority of tumors regardless of age or treatment status (Table 3a). Among early-onset cases, alterations were present in 95.9% of FOLFOX-treated tumors and all of the tumors not exposed to FOLFOX, with no statistically significant difference between groups. A similar pattern was observed in late-onset H/L disease, where MAPK alterations were identified in 97.8% of FOLFOX-treated tumors and all of the non-FOLFOX-treated tumors. These findings indicate that MAPK pathway involvement is nearly ubiquitous in H/L CRC, independent of age at diagnosis or chemotherapy exposure.

Comparable results were observed in the NHW cohort (Table 3b). MAPK alterations were present in approximately 96% of tumors across all NHW subgroups, including early- and late-onset disease and regardless of FOLFOX treatment status. No significant differences in pathway alteration prevalence were detected between treated and untreated patients within either age category, suggesting that MAPK pathway engagement is a common feature of NHW CRC tumors across the disease spectrum.

#### 3.3.2. Ancestry-Based Comparisons Within Age and Treatment Strata

Direct comparisons between H/L and NHW patients further underscored the consistency of MAPK pathway alteration prevalence across populations. Among early-onset CRC patients treated with FOLFOX, MAPK alterations were observed at nearly identical frequencies in H/L and NHW tumors (95.9% vs. 96.3%, respectively; Table 3c). Similarly, in early-onset patients not treated with FOLFOX, pathway alterations were detected in all of the H/L tumors and 96.4% of NHW tumors, without a statistically significant difference.

Ancestry-based comparisons in late-onset CRC followed the same trend (Table 3d). MAPK alterations were present in 97.8% of FOLFOX-treated H/L tumors and 96.3% of treated NHW tumors, while non-FOLFOX-treated late-onset tumors showed alteration frequencies of 100% in H/L patients and 96.3% in NHW patients. Across all comparisons, differences in MAPK pathway alteration prevalence by ancestry did not reach statistical significance.

#### 3.3.3. Interpretation and Integration with Gene-Level Findings

Taken together, these results demonstrate that MAPK pathway alterations are nearly universal in colorectal cancer, irrespective of age at onset, ancestry, or FOLFOX exposure. While pathway-level alteration prevalence does not discriminate between clinical subgroups, this apparent uniformity contrasts with the substantial gene-level heterogeneity identified earlier, where specific MAPK components (e.g., *NF1, PDGFRB, MAPK3, RPS6KA4, JUN*) showed strong age-, ancestry-, and treatment-dependent enrichment. These findings suggest that, although MAPK signaling is broadly implicated across CRC, the specific molecular mechanisms driving pathway dysregulation differ by clinical context, reinforcing the importance of gene-level and subgroup-aware analyses in precision oncology.

### 3.4. Gene-Level Landscape of MAPK Pathway Alterations Across Age, Ancestry, and FOLFOX Exposure

Gene-level interrogation of the MAPK pathway (Tables S1–S12) showed that, despite near-universal pathway-level alteration positivity across strata, most individual genes occurred at low-to-moderate frequencies, with a limited set exhibiting statistically significant enrichment by age, ancestry, and/or treatment status.

Within early-onset H/L tumors (Table S1), most MAPK genes were infrequently mutated and did not differ by FOLFOX exposure. However, three loci showed treatment-associated enrichment in the non-FOLFOX subgroup: *FGFR2* (7.7% in non-FOLFOX vs. 0% in FOLFOX; *p* = 0.02793), *NF1* (19.2% vs. 4.1%; *p* = 0.01437), and *RPS6KA4* (9.6% vs. 0%; *p* = 0.01108). Core drivers remained common in both groups, including *KRAS* (41.1% FOLFOX vs. 34.6% non-FOLFOX) and *TP53* (~78–81%). In late-onset H/L tumors (Table S2), treatment-associated differences were generally absent, with the exception of increased *NTRK2* (6.0% vs. 0%; *p* = 0.04286) and *PDGFRB* (6.0% vs. 0%; *p* = 0.04286) in the non-FOLFOX group; *BRAF* was notably higher in late-onset H/L overall (16.0–16.5%) than in early-onset H/L (4.1–7.7%), independent of therapy.

Among early-onset NHW tumors (Table S3), the MAPK gene spectrum was similarly stable across FOLFOX strata, but significant differences emerged for select genes: *AKT3* mutations were more frequent in non-FOLFOX cases (3.0% vs. 0.8%; *p* = 0.04069), *FGF4* was detected only in non-FOLFOX tumors (1.7% vs. 0%; *p* = 0.01734), *JUN* was enriched in FOLFOX-treated tumors (3.2% vs. 0.7%; *p* = 0.02726), and *RRAS2* occurred exclusively in non-FOLFOX tumors (1.3% vs. 0%; *p* = 0.03916). In late-onset NHW tumors (Table S4), multiple genes differed by treatment status, with higher prevalence in non-FOLFOX cases for *CRKL* (1.2% vs. 0.1%; *p* = 0.004928), *DUSP4* (0.9% vs. 0.1%; *p* = 0.02292), *JUN* (3.2% vs. 1.4%; *p* = 0.02486), *MAPK1* (0.9% vs. 0.1%; *p* = 0.02292), *RAF1* (2.9% vs. 0.9%; *p* = 0.004111), *RPS6KA4* (5.2% vs. 1.8%; *p* = 0.0003744), *RRAS* (1.2% vs. 0.2%; *p* = 0.02024), and *SOS1* (2.3% vs. 0.9%; *p* = 0.03501). Additionally, *TP53* was modestly higher in FOLFOX-treated late-onset NHW tumors (73.4% vs. 68.1%; *p* = 0.02559), and *TGFBR2* was higher in non-FOLFOX cases (7.0% vs. 4.1%; *p* = 0.01578).

Age-stratified analyses reinforced age-associated biology within treatment strata. Among FOLFOX-treated H/L cases (Table S5), *BRAF* was significantly higher in late-onset compared with early-onset tumors (16.5% vs. 4.1%; *p* = 0.01229), and *PDGFRB* was observed only in early-onset tumors (5.5% vs. 0%; *p* = 0.03747). Among non-FOLFOX H/L cases (Table S6), NF1 remained enriched in early-onset disease (19.2% vs. 4.0%; *p* = 0.02834), consistent with a stronger *NF1* signal in younger untreated H/L tumors. In the NHW cohort, age comparisons in both treated (Table S7) and untreated (Table S8) strata also supported increased *BRAF* burden with later onset: *BRAF* was higher in late-onset versus early-onset non-FOLFOX NHW tumors (14.2% vs. 7.9%; *p* = 0.007983), whereas gene-level differences were otherwise limited.

Direct ancestry comparisons within EOCRC showed that most MAPK genes were broadly similar between H/L and NHW within each treatment group (Tables S9–S11). Nonetheless, ancestry-specific signals were evident in non-FOLFOX EOCRC, where H/L tumors showed higher frequencies of *MAPK3* (5.8% vs. 1.0%; *p* = 0.04335), *NF1* (19.2% vs. 6.0%; *p* = 0.002728), and *RPS6KA4* (9.6% vs. 2.6%; *p* = 0.03866) (Table S10). In contrast, in FOLFOX-treated EOCRC, *PDGFRB* was higher in H/L than NHW tumors (5.5% vs. 0.8%; *p* = 0.01547) (Table S9). In late-onset non-FOLFOX comparisons (Table S12), *NTRK2* was more frequent in H/L than NHW (6.0% vs. 1.4%; *p* = 0.0472), and *RRAS2* was enriched in H/L (4.0% vs. 0.5%; *p* = 0.04318), while most other loci did not differ by ancestry.

These stratified gene-level results indicate that MAPK pathway disruption in CRC is ubiquitous at the pathway level, but specific nodes, particularly *NF1, FGFR2, RPS6KA4, BRAF*, and select RTK/adaptor genes (*PDGFRB, NTRK2, RAF1, SOS1, CRKL*), show context-dependent enrichment by age, ancestry, and chemotherapy exposure.

### 3.5. MAPK Pathway Mutation Profile

#### 3.5.1. Early-Onset Hispanic/Latino Colorectal Cancer

**Figure 1:**
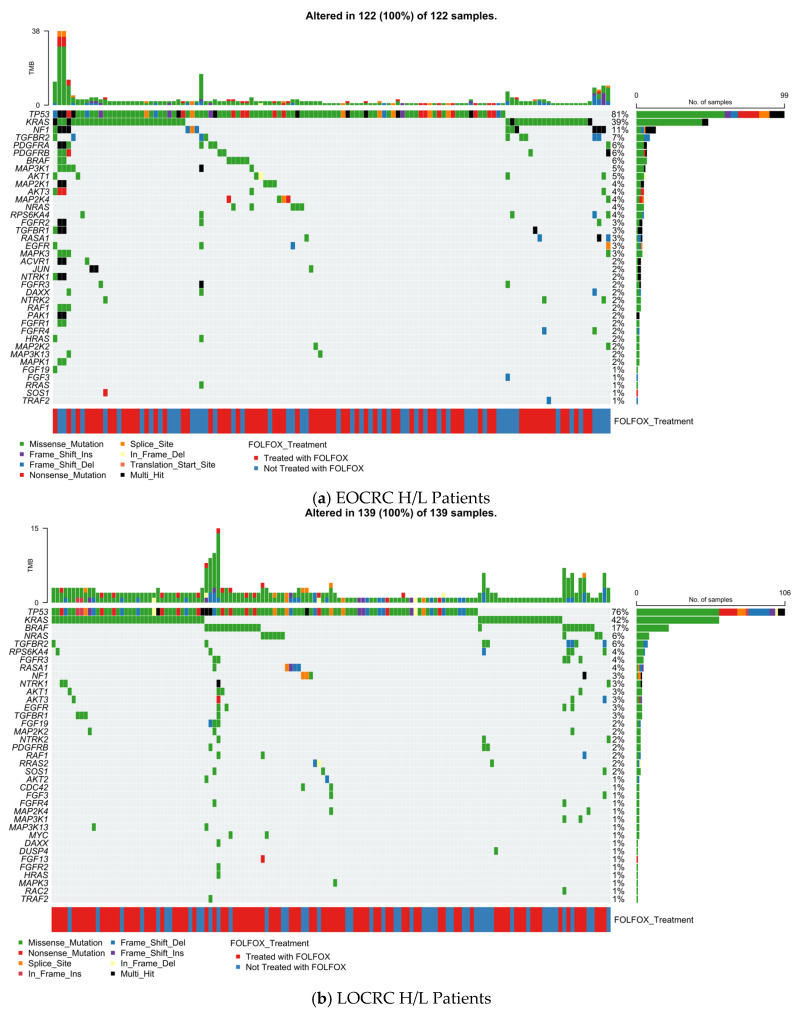

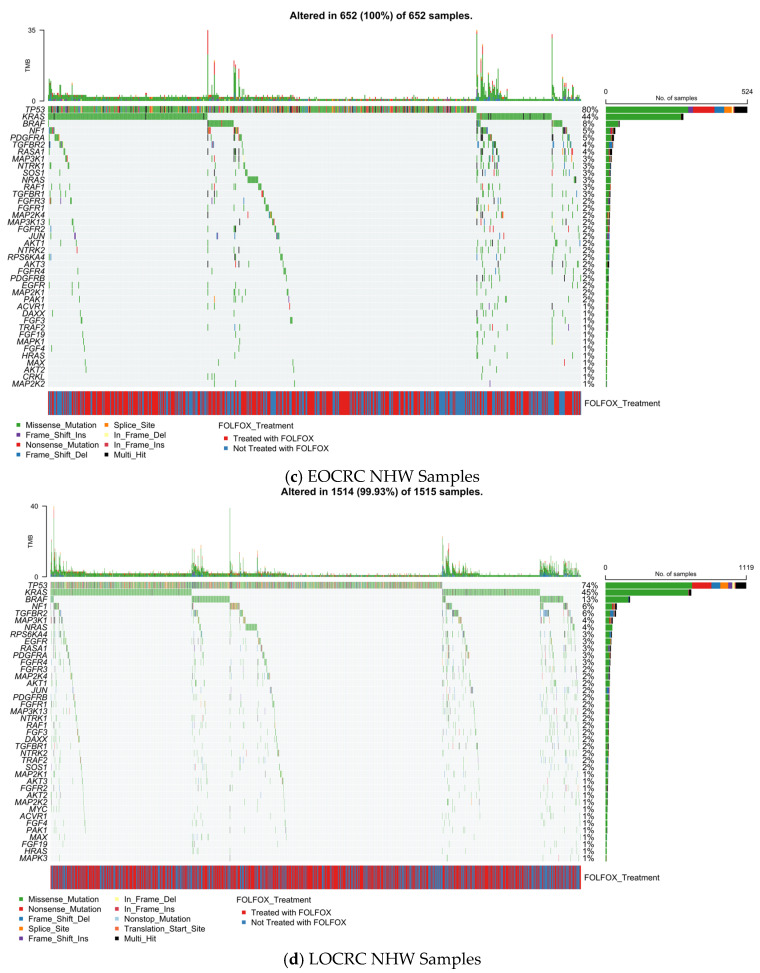
MAPK pathway mutational landscapes across colorectal cancer subgroups defined by age of onset and ancestry. This figure displays oncoplot visualizations summarizing somatic mutations in genes involved in MAPK signaling across colorectal cancer (CRC) cohorts stratified by diagnostic age and ancestral background. Separate panels illustrate mutation patterns for early-onset and late-onset CRC within Hispanic/Latino (H/L) and non-Hispanic White (NHW) populations. Each oncoplot integrates variant class, TMB, and FOLFOX chemotherapy exposure, enabling direct comparison of MAPK pathway disruption across clinically and demographically distinct groups. Panels correspond to: (**a**) early-onset H/L CRC, (**b**) late-onset H/L CRC, (**c**) early-onset NHW CRC, and (**d**) late-onset NHW CRC.

Figure 1a presents an integrated overview of somatic alterations affecting MAPK pathway genes in EOCRC H/L patients (EOCRC; n = 122), combining mutation type, TMB, and FOLFOX treatment annotation. Unlike signaling pathways that are altered only in a subset of tumors, the MAPK pathway involvement was universal in this cohort, with every tumor (122/122; 100%) harboring at least one alteration within the curated MAPK gene set.

The mutational landscape was anchored by well-established colorectal cancer drivers. TP53 was altered most frequently (81%), followed by KRAS (39%). Additional recurrent events involved regulators and modulators of MAPK signaling, including *NF1* and *TGFBR2* (each 11%). A broader distribution of less frequent alterations spanned both upstream receptor tyrosine kinases and downstream signaling intermediates, such as *PDGFRA* (~7%) and a group of genes altered in approximately 5–6% of tumors (*PDGFRB, BRAF, MAP3K1, AKT1, MAP2K1*). Numerous other pathway components, including *NRAS, MAP2K4, RPS6KA4, FGFR2, TGFBR1, RASA1, EGFR*, and *MAPK3*, were affected at lower frequencies (typically 2–4%), reflecting substantial inter-tumor heterogeneity within a broadly perturbed pathway.

At the variant level, missense substitutions constituted the predominant mutation class, with fewer loss-of-function events such as frame-shift insertions or deletions, nonsense mutations, and sporadic splice-site variants. Multi-hit alterations were rare and restricted to individual cases. TMB values spanned a wide range, including a subset of tumors with elevated mutational burden; however, these hypermutated samples did not show preferential association with any specific MAPK gene, indicating that increased TMB reflects global genomic instability rather than selective MAPK pathway hypermutation (Table S13).

Assessment of chemotherapy exposure revealed no overt segregation of MAPK alterations by FOLFOX treatment status. Treated and untreated tumors were evenly distributed across the mutational spectrum, suggesting that, in EOCRC H/L patients, MAPK pathway disruption is widespread, diverse, and not visually driven by chemotherapy exposure at the pathway-wide level.

#### 3.5.2. Late-Onset Hispanic/Latino CRC

Figure 1b depicts the MAPK pathway mutational profile of LOCRC H/L tumors (n = 139), integrating somatic variant classes, TMB, and FOLFOX treatment status. Consistent with findings in early-onset disease, MAPK pathway alterations were observed in all tumors (139/139; 100%), underscoring the pervasive involvement of MAPK signaling in H/L CRC across age groups.

The mutational spectrum in late-onset H/L CRC was dominated by canonical CRC drivers and core MAPK-associated genes. *TP53* was the most frequently altered gene (76%), followed by *KRAS* (42%) and *BRAF* (17%), reflecting a higher prevalence of *BRAF* alterations compared with early-onset H/L tumors. Additional recurrent alterations were observed in *NRAS* (~7%) and *TGFBR2* (~6%), with a broader distribution of lower-frequency mutations across multiple pathway regulators and effectors, including *RPS6KA4, FGFR2, RASA1, NF1, NTRK1, AKT3, EGFR, TGFBR1, MAP2K2,* and *NTRK2* (generally ~2–4% each). This pattern illustrates a more diversified MAPK mutational landscape in late-onset disease, characterized by contributions from both upstream receptor tyrosine kinases and downstream signaling intermediates.

As in early-onset tumors, missense mutations (green) constituted the majority of events, with fewer truncating alterations such as frame-shift insertions or deletions (blue shades) and nonsense mutations (red). Multi-hit events were uncommon and did not cluster within specific genes. TMB values were generally modest across the cohort, with a subset of tumors exhibiting elevated mutational burden; however, these higher-TMB cases were not preferentially associated with any single MAPK gene, suggesting that increased TMB reflects global genomic instability rather than focal MAPK hypermutation (Table S13).

The FOLFOX treatment annotation (blue = treated; red = not treated) was distributed across the cohort without an apparent segregation of MAPK alterations by chemotherapy exposure. Both treated and untreated tumors displayed comparable patterns of pathway involvement, indicating that, at the pathway-wide level, MAPK alterations in late-onset H/L CRC are age-associated but not visually driven by FOLFOX status.

The LOCRC H/L MAPK mutational landscape reveals a broader and more BRAF-enriched profile compared with early-onset disease, reinforcing age-related differences in MAPK pathway architecture while highlighting the ubiquitous role of MAPK signaling in colorectal tumorigenesis within H/L populations.

#### 3.5.3. Early-Onset Non-Hispanic White CRC

Figure 1c illustrates the MAPK pathway mutational landscape in EOCRC NHW tumors (n = 652), integrating gene-level alterations, TMB, and FOLFOX treatment status. In line with pathway-level analyses, MAPK pathway alterations were present in all tumors (652/652; 100%), confirming widespread disruption of MAPK signaling in early-onset disease within the NHW population.

The mutational profile was dominated by well-established CRC driver genes. TP53 alterations were observed in approximately 80% of tumors, followed by *KRAS* (48%) and *BRAF* (8%), reflecting a lower *BRAF* frequency compared with late-onset NHW disease and late-onset H/L tumors. Additional recurrent alterations were distributed across multiple MAPK-associated genes, including *NF1* (~5%), *PDGFRA* (~5%), *RASGRF1, RASA1, MAP3K1, NTRK1, NRAS, SOS1*, and *TGFBR2* (each ~3–4%). A broad tail of lower-frequency mutations (<3%) affected both upstream receptor tyrosine kinases (e.g., *EGFR, FGFR1/2*) and downstream signaling components (e.g., *MAP2K1, MAP3K3, AKT3, RPS6KA4*), underscoring substantial inter-tumor heterogeneity.

Across the cohort, missense mutations (green) represented the predominant alteration type, with fewer nonsense mutations, frame-shift insertions/deletions, and occasional splice-site events. Multi-hit alterations were infrequent and did not cluster within specific genes. TMB values spanned a wide range, including a subset of hypermutated tumors characterized by prominent TMB peaks; however, these high-TMB cases did not preferentially harbor mutations in any single MAPK gene, suggesting that elevated mutational load reflects generalized genomic instability rather than focal MAPK pathway hypermutation (Table S13).

The FOLFOX treatment annotation track (blue = treated; red = not treated) was evenly interspersed across samples, with no clear segregation of MAPK alteration patterns by chemotherapy exposure at the pathway-wide level. Both treated and untreated tumors exhibited similar distributions of driver and non-driver MAPK alterations, indicating that early-onset NHW MAPK genomic architecture is largely independent of FOLFOX status when assessed globally.

Early-onset NHW CRC demonstrates a KRAS- and TP53-dominated MAPK mutational profile, with lower BRAF involvement and extensive low-frequency alterations across the pathway. When considered alongside ancestry-comparative analyses, these findings highlight both shared MAPK pathway dependence across populations and subtle differences in the relative contribution of specific MAPK nodes to EOCRC tumorigenesis.

#### 3.5.4. Late-Onset Non-Hispanic White CRC

Figure 1d depicts the MAPK pathway mutational architecture of LOCRC NHW tumors (n = 1515), integrating somatic variant types, TMB, and FOLFOX treatment annotation. Consistent with pathway-level analyses, MAPK pathway alterations were detected in nearly all tumors (1514/1515; 99.9%), reinforcing the central role of MAPK signaling in late-onset CRC within the NHW population.

The mutational spectrum was dominated by canonical CRC drivers. *TP53* alterations were the most prevalent (~74%), followed by *KRAS* (~43%) and *BRAF* (~15%), reflecting a marked enrichment of *BRAF* mutations relative to early-onset NHW disease. Additional recurrent MAPK-associated alterations were observed in *NF1* (~6%), *TGFBR2* (~5%), *MAP3K1* (~4%), *NRAS* (~4%), and *RPS6KA4* (~3%), with a wide distribution of lower-frequency events affecting receptor tyrosine kinases and signaling intermediates such as *PDGFRA, FGFR4, MAP2K1, MAPK3, AKT1, JUN, PDGFRB, EGFR, MAP3K3,* and *NTRK1* (generally ~1–3%). This pattern indicates a broad and heterogeneous disruption of MAPK signaling nodes in late-onset NHW tumors.

As observed in other cohorts, missense mutations (green) accounted for the majority of alterations, accompanied by less frequent truncating events, including frame-shift insertions/deletions, nonsense mutations, and occasional splice-site variants. Multi-hit alterations were rare and did not cluster within specific MAPK genes. TMB values ranged from low to moderately elevated, with a subset of hypermutated tumors visible as prominent TMB peaks. However, these high-TMB samples were not preferentially associated with mutations in any single MAPK pathway component, suggesting that increased mutational load reflects global genomic instability rather than selective MAPK hypermutation (Table S13).

The FOLFOX treatment annotation track (blue = treated; red = not treated) was evenly interspersed across the cohort, with no clear segregation of MAPK alteration patterns by chemotherapy exposure at the pathway-wide level. Both treated and untreated tumors demonstrated comparable distributions of high-frequency drivers and lower-frequency MAPK alterations.

LOCRC NHW exhibits a TP53-KRAS-BRAF-enriched MAPK mutational profile with extensive low-frequency pathway perturbations. Compared with early-onset NHW disease, late-onset tumors show a higher contribution of *BRAF* and *NF1* alterations, consistent with age-associated shifts in MAPK pathway biology, while maintaining broad pathway involvement independent of FOLFOX treatment status.

### 3.6. Survival Associations of MAPK Pathway Alterations

To evaluate the prognostic relevance of MAPK pathway alterations in colorectal cancer, we conducted Kaplan–Meier analyses stratified by age of onset, ancestry, and FOLFOX treatment exposure.

#### 3.6.1. Early-Onset Hispanic/Latino Patients Receiving FOLFOX

**Figure 2:**
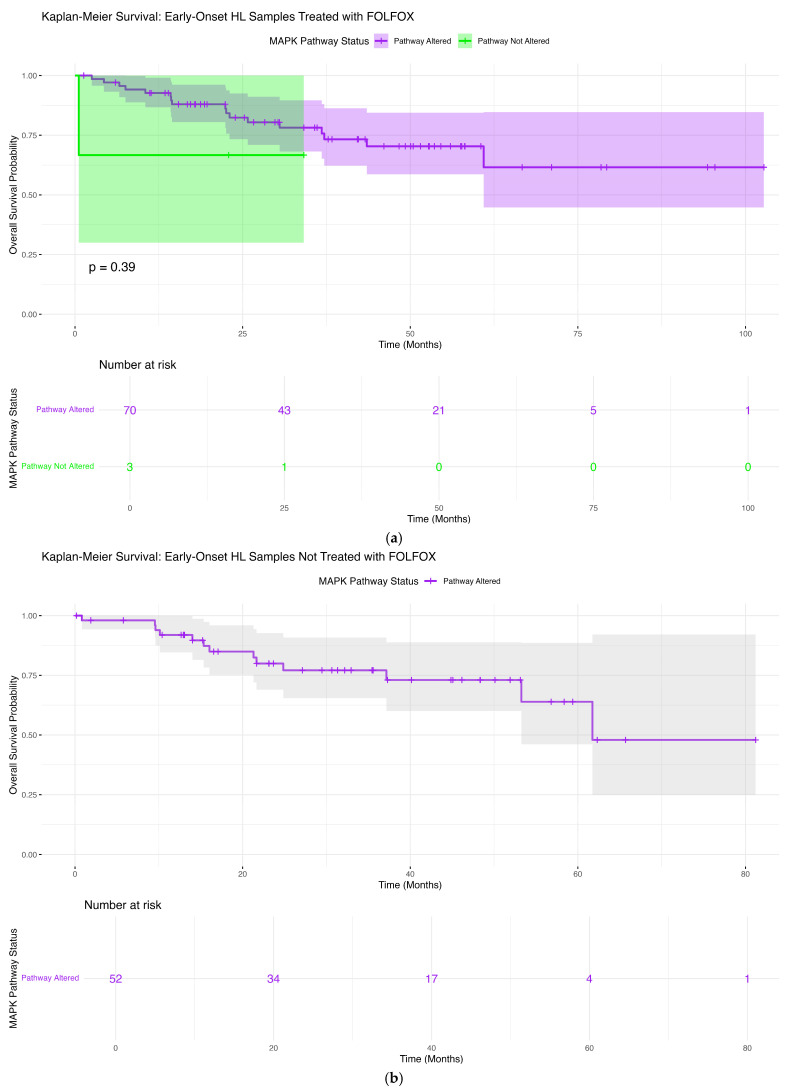

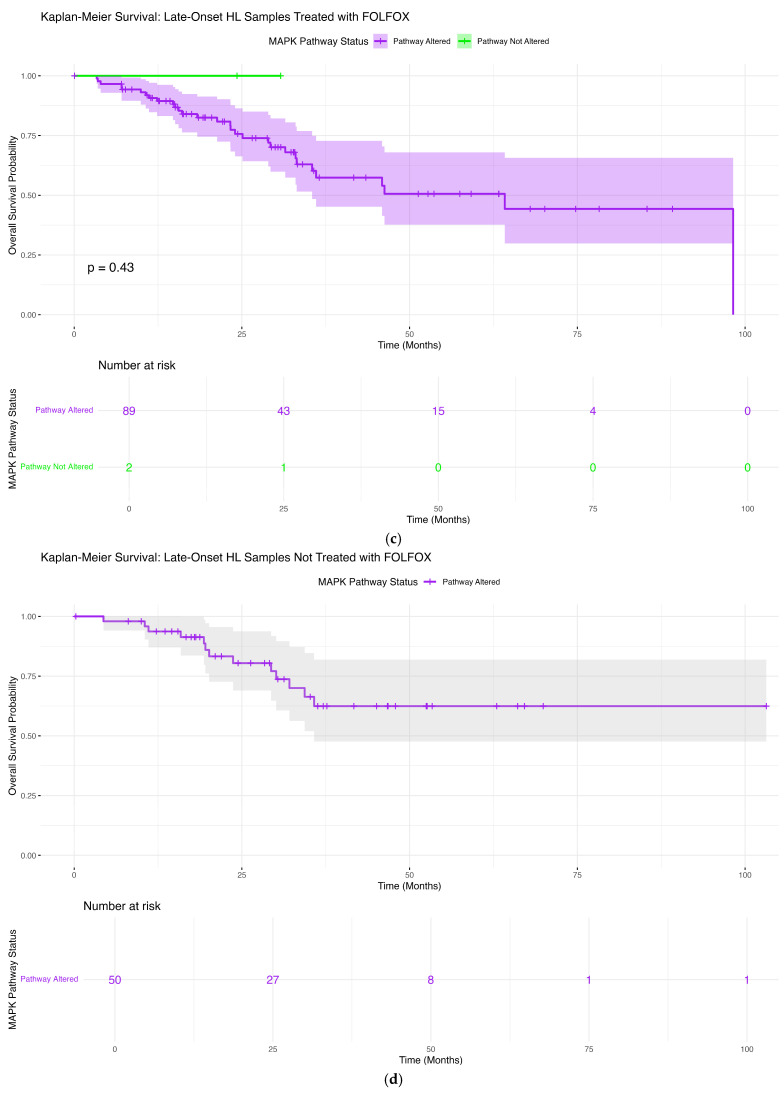

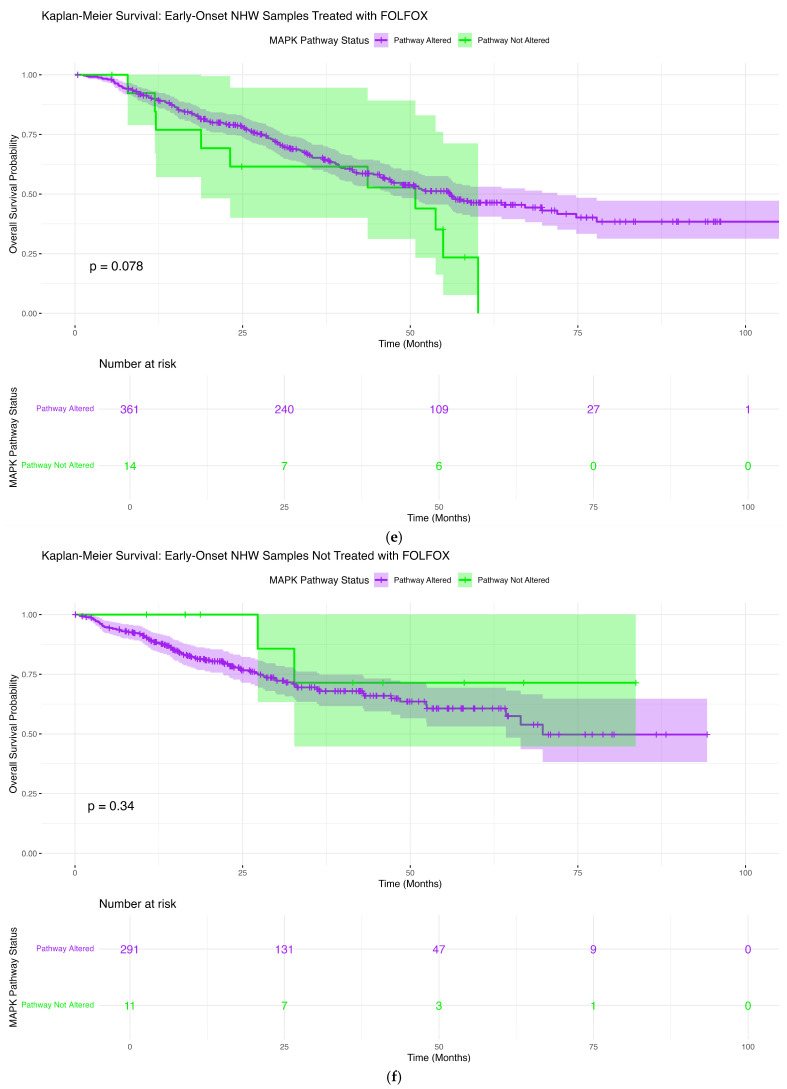

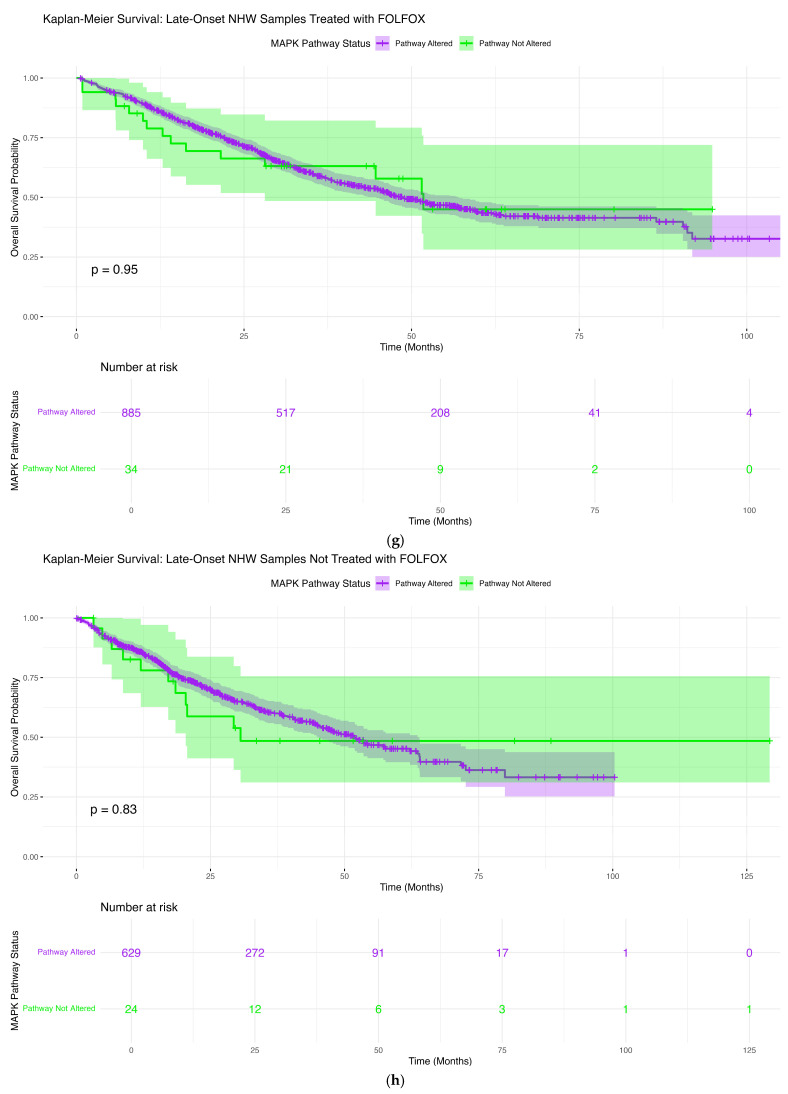
Overall survival by MAPK pathway alteration status across colorectal cancer subgroups defined by age, ancestry, and chemotherapy exposure. This figure presents Kaplan–Meier survival analyses comparing overall survival between colorectal cancer (CRC) patients with and without MAPK pathway alterations, stratified by age at diagnosis, ancestral background, and FOLFOX treatment status. Survival curves are shown for the following subgroups: (**a**) early-onset Hispanic/Latino patients receiving FOLFOX; (**b**) early-onset Hispanic/Latino patients not exposed to FOLFOX; (**c**) late-onset Hispanic/Latino patients treated with FOLFOX; (**d**) late-onset Hispanic/Latino patients not treated with FOLFOX; (**e**) early-onset non-Hispanic White patients receiving FOLFOX; (**f**) early-onset non-Hispanic White patients not receiving FOLFOX; (**g**) late-onset non-Hispanic White patients treated with FOLFOX; and (**h**) late-onset non-Hispanic White patients not treated with FOLFOX. Within each panel, outcomes for tumors harboring MAPK pathway mutations are contrasted with those lacking detectable alterations. Shaded regions denote 95% confidence intervals, and tabulated counts beneath each plot indicate the number of individuals at risk over time, enabling assessment of survival dynamics within each clinically and demographically defined subgroup.

In EOCRC H/L patients treated with FOLFOX (Figure 2a), overall survival did not differ significantly according to MAPK pathway mutation status (*p* = 0.39). Survival trajectories for tumors harboring MAPK alterations and those without detectable pathway mutations followed largely overlapping courses across the duration of follow-up, indicating no clear separation between groups.

Patients with MAPK-altered tumors demonstrated broader confidence intervals, reflecting the smaller size of the non-altered comparator group and increased uncertainty at later time points. Despite this variability, long-term survival probabilities remained comparable between cohorts, with no consistent trend toward improved or worsened outcomes associated with MAPK pathway disruption. The non-altered group showed a modest decline in survival beyond approximately 25–30 months, though overall survival remained high.

These results suggest that, among early-onset H/L patients receiving FOLFOX, MAPK pathway alteration status does not confer a statistically significant prognostic effect on overall survival. These findings should be interpreted in the context of limited sample sizes in the non-altered subgroup but indicate that, within this treatment-defined population, MAPK pathway involvement alone is insufficient to stratify survival risk.

#### 3.6.2. Early-Onset Hispanic/Latino Patients Without FOLFOX Exposure

Among EOCRC H/L patients who did not undergo FOLFOX treatment (Figure 2b), overall survival patterns were predominantly uniform across MAPK pathway subgroups. All tumors within this cohort harbored MAPK pathway alterations, preventing pathway-based stratification and precluding a direct comparison between altered and non-altered outcomes.

The survival curve for the MAPK-altered cohort showed a gradual decline over time, with the majority of patients maintaining high survival probability during the initial follow-up period. Confidence intervals widened notably at later time points, reflecting attrition in the number of individuals remaining under observation. While survival probability decreased after approximately 50–60 months, no distinct inflection point or abrupt change in trajectory was observed.

Although these findings reinforce the pervasive presence of MAPK alterations in early-onset H/L CRC, they also indicate that, within this untreated subgroup, pathway alteration status does not meaningfully differentiate survival risk. The absence of non-altered comparators, combined with broad confidence intervals and declining sample size over time, limits definitive interpretation but supports the conclusion that MAPK disruption, at a pathway-wide level, is not a primary determinant of survival outcomes in early-onset H/L tumors lacking FOLFOX exposure.

#### 3.6.3. Late-Onset Hispanic/Latino Patients Receiving FOLFOX

Among LOCRC H/L patients treated with FOLFOX (Figure 2c), survival outcomes appeared broadly similar regardless of MAPK pathway alteration status. No statistically significant difference in overall survival was observed between MAPK-altered and non-altered groups (*p* = 0.43), indicating an absence of clear prognostic discrimination within this subgroup.

The survival curve for MAPK-altered tumors demonstrated a steady decline across the follow-up period, while the curve for non-altered tumors, represented by a small comparator subset, remained relatively stable early on before converging toward the altered trajectory at later time points. Wide confidence intervals, particularly for the non-altered samples, highlighted the small number of individuals without MAPK pathway alterations and contributed to uncertainty in the survival estimates.

Despite visible variability in curve shape, the overlapping confidence bands and similar long-term survival probabilities suggest that MAPK mutation status does not meaningfully influence prognosis among late-onset H/L patients undergoing FOLFOX therapy. As in other subgroups, sample size imbalance limits the precision of comparative interpretation; however, the overall pattern supports the conclusion that MAPK pathway disruption, at the pathway level, is not a dominant survival determinant in this clinical context.

#### 3.6.4. Late-Onset Hispanic/Latino Patients Without FOLFOX Treatment

In the subgroup of LOCRC H/L patients who did not receive FOLFOX (Figure 2d), overall survival outcomes were largely uniform, with no apparent variation attributable to MAPK pathway mutation status. Because all tumors in this subgroup carried MAPK pathway alterations, a non-altered comparator group was not present, preventing pathway-based stratification within the cohort.

The survival curve displayed a gradual early decline followed by stabilization across later follow-up intervals. Confidence intervals widened over time, reflecting fewer individuals remaining under observation at later survival points, but overall survival probabilities stayed relatively consistent throughout the duration assessed.

These findings highlight the widespread presence of MAPK alterations in LOCRC H/L patients and indicate that, in the absence of FOLFOX chemotherapy, MAPK pathway involvement does not visibly distinguish survival trajectories within this subgroup. However, the absence of a non-altered comparison cohort limits the ability to draw conclusions regarding the prognostic value of these alterations in untreated late-onset H/L tumors.

#### 3.6.5. Early-Onset Non-Hispanic White Patients Treated with FOLFOX

Among EOCRC NHW patients receiving FOLFOX chemotherapy (Figure 2e), overall survival patterns showed modest divergence based on MAPK pathway status. Although the comparison did not reach statistical significance (*p* = 0.078), visual inspection of the Kaplan–Meier curves revealed trends worth noting.

Patients with MAPK-altered tumors demonstrated a gradual but steady decline in survival over the full follow-up period, maintaining intermediate survival probabilities throughout. In contrast, the non-altered group, although considerably smaller in size, displayed a steeper drop in survival within the first 40 months, followed by stabilization at lower survival levels. By later follow-up points, confidence intervals widened substantially for both curves, particularly for the non-altered subgroup, reflecting diminishing sample size and reduced precision of estimates.

Despite the absence of a statistically significant result, the widening separation between curves over time suggests a potential biological distinction between altered and non-altered MAPK groups in this setting. However, given the small number of MAPK-non-altered cases and wide variability at later time points, these observations should be interpreted cautiously. Taken together, the data indicate that while MAPK mutation status does not definitively stratify survival outcomes in FOLFOX-treated EOCRC NHW patients, pathway differences may contribute to subtle survival variation that warrants further investigation in larger cohorts.

#### 3.6.6. Early-Onset NHW Patients Not Treated with FOLFOX

For EOCRC NHW patients who did not receive FOLFOX chemotherapy (Figure 2f), overall survival outcomes did not show a statistically meaningful association with MAPK pathway alteration status (*p* = 0.34). Both MAPK-altered and non-altered groups began with similarly high survival probabilities and followed closely aligned trajectories across the observation period.

A slight divergence appeared midway through follow-up, during which the non-altered subgroup showed somewhat higher survival probabilities than the altered cohort. However, this separation remained modest and transient, and confidence intervals surrounding both curves were wide and largely overlapping, particularly for the smaller non-altered group, underscoring notable variability and limited statistical power.

By late follow-up, survival estimates converged again between groups, further supporting the absence of a strong survival distinction driven by MAPK pathway status in this clinical context. Overall, these findings suggest that, among EOCRC NHW patients who did not receive FOLFOX, MAPK alterations do not exert a clear or measurable influence on overall survival, though small subgroup sizes warrant cautious interpretation.

#### 3.6.7. Late-Onset NHW Patients Treated with FOLFOX

Among LOCRC NHW patients treated with FOLFOX chemotherapy (Figure 2g), overall survival showed no meaningful distinction based on MAPK pathway mutation status. Survival curves for MAPK-altered and non-altered tumors were nearly superimposed throughout the observation window, with both groups demonstrating a steady decline over time and comparable long-term outcomes (*p* = 0.95).

Confidence intervals for the two trajectories overlapped extensively, reinforcing the lack of separation between groups. Although minor variations were visible in early and mid-follow-up, these fluctuations did not persist and did not suggest any consistent advantage or disadvantage tied to pathway alteration.

These findings indicate that, within this large LOCRC NHW cohort receiving FOLFOX, MAPK pathway mutation status does not appear to influence overall survival, highlighting a largely uniform response pattern to chemotherapy regardless of underlying MAPK alteration profile.

#### 3.6.8. Late-Onset NHW Patients Not Treated with FOLFOX

In LOCRC NHW patients who did not receive FOLFOX chemotherapy (Figure 2h), overall survival outcomes were broadly similar between tumors with and without MAPK pathway alterations (*p* = 0.83). Both trajectories began with near-complete survival and exhibited a steady decline over time, with no durable separation emerging between altered and non-altered groups.

Although the MAPK-altered cohort showed a slightly steeper drop in survival during mid-follow-up, confidence intervals for both groups overlapped extensively across the entire observation period, indicating substantial uncertainty and no reliable evidence of prognostic divergence. The non-altered subgroup, though smaller in size, demonstrated wide confidence shading, reflecting reduced precision in later time points but still aligning closely with the altered group’s survival estimate.

These results suggest that MAPK pathway mutation status does not impart a measurable survival difference in untreated LOCRC NHW patients, reinforcing the broader observation that MAPK alterations, while frequent, may not independently predict outcome in the absence of FOLFOX exposure.

### 3.7. Conversational Artificial Intelligence

#### 3.7.1. AI-Enabled Exploratory Cohort Stratification and Survival Assessment

To generate preliminary clinical insight prior to pathway-specific modeling, we used the conversational AI framework to rapidly construct ancestry-matched survival cohorts and perform exploratory outcome comparisons. This approach allowed real-time interrogation of integrated clinical-genomic CRC data while preserving strict user-defined eligibility rules.

Figure S1 provides one such example, focusing on early-onset CRC patients treated with FOLFOX chemotherapy. The AI platform automatically identified: 73 early-onset H/L FOLFOX-treated cases (panel a), representing 2.9% of available samples in this subgroup, and 375 early-onset NHW FOLFOX-treated controls (panel b), accounting for 14.9% of records within the corresponding ancestry group. Kaplan–Meier analysis performed through the platform revealed a clear separation in overall survival trajectories between the two ancestry groups (panel c), with significantly improved outcomes observed in the H/L cohort (log-rank *p* = 0.0310). The widening confidence bands over longer follow-up reflect decreasing patient numbers at risk but do not obscure the durable survival advantage.

This exploratory result demonstrates how real-time AI-directed cohort refinement can uncover clinically meaningful ancestry-based differences within treatment-defined strata. These findings provided the rationale for subsequent MAPK-focused analyses and highlight the platform’s utility in rapidly identifying outcome patterns that warrant deeper mechanistic and statistical investigation.

#### 3.7.2. AI-Assisted Comparison of MSI Stability in Early-Onset Hispanic/Latino CRC Stratified by Treatment Exposure

To further evaluate molecular differences within ancestry-defined subgroups, we applied the conversational AI framework to examine whether microsatellite stability patterns varied by chemotherapy exposure among early-onset H/L colorectal cancer patients. Using natural language-directed cohort construction, the system identified two treatment-defined populations: those who received FOLFOX (n = 73) and those who did not (n = 52).

Panels (a) and (b) in Figure S2 illustrate the proportions of cases that met the inclusion criteria relative to all available samples, confirming that both subgroups represented a small and comparable fraction of the dataset.

A contingency comparison was then conducted to assess whether MSI-stable tumor status differed between treated and untreated groups. As shown in panel (c), MSI-stable tumors were distributed similarly across cohorts, and Fisher’s exact testing demonstrated no statistically significant association between MSI category and treatment status (*p* = 0.785). Odds ratio outputs likewise indicated no directional trend favoring either subgroup.

These findings suggest that, within early-onset H/L CRC, MSI stability is not influenced by FOLFOX exposure, reinforcing the interpretation that treatment alone does not drive detectable variation in MSI-related tumor biology in this population. This analysis also highlights the practical value of the AI-guided workflow in rapidly generating cohort-specific molecular comparisons and validating null relationships within highly granular clinical strata.

#### 3.7.3. AI-Assisted Exploratory Comparison of AKT3 Mutation Frequency in Early-Onset NHW CRC by Treatment Exposure

As part of our exploratory computational workflow, we applied the conversational AI framework to interrogate whether *AKT3* mutations varied according to chemotherapy exposure within early-onset NHW colorectal cancer patients. Using AI-directed cohort filtering, the system automatically identified two treatment-defined groups: individuals who received FOLFOX (n = 375) and those who did not (n = 302) (Figure S3).

Panels (a) and (b) illustrate the proportion of samples in each cohort that met the AKT3 mutation criterion, confirming that only a small minority of tumors were classified as AKT3-altered. Notably, mutation prevalence was slightly lower in the FOLFOX-treated cohort (14.9%) compared with untreated patients (12.0%), although both groups showed similar overall distributions.

A stacked bar chart (panel c) summarizes results from an odds ratio comparison using Fisher’s exact test. Statistical evaluation revealed no significant association between treatment exposure and AKT3 alteration status (*p* = 0.065), indicating that AKT3 mutation patterns remain largely stable regardless of chemotherapy history. While the numerical signal suggested marginally reduced alteration rates in treated cases, confidence interval width and *p*-value trends supported the interpretation that differences were not meaningful.

Taken together, this AI-mediated analysis demonstrates that AKT3 mutation frequency does not appear to differ substantially between treated and untreated early-onset NHW CRC patients, and further highlights the utility of rapid, query-driven cohort generation for validating molecular equivalence or divergence across clinically relevant patient subgroups.

#### 3.7.4. AI-Guided Exploratory Comparison of PDGFRB Alterations in Early-Onset FOLFOX-Treated Patients Across Ancestry Groups

As part of the exploratory AI interrogation, we evaluated whether PDGFRB mutation prevalence differed between early-onset H/L and NHW colorectal cancer patients treated with FOLFOX (Figure S4). Using conversational filtering, the AI platform automatically assembled a case cohort of H/L patients (n = 73) and a control cohort of NHW patients (n = 375) meeting identical clinical conditions, ensuring that treatment status and age of onset were matched across ancestry groups. Visual inspection of the pie charts in panels (a) and (b) confirmed accurate extraction of the relevant comparison subsets, with only a small fraction of individuals in either cohort exhibiting *PDGFRB* alterations.

Subsequent odds ratio analysis is summarized in panel (c), demonstrating that *PDGFRB* mutations were uncommon in both ancestry-defined cohorts. Mutation frequency was numerically lower among H/L patients, but Fisher’s exact testing indicated no statistically significant difference between groups (*p* = 0.502), and the odds ratio suggested a minimal effect size consistent with rare-event variability.

Taken together, these exploratory results indicate that *PDGFRB* mutation patterns appear largely equivalent across ancestry in early-onset FOLFOX-treated CRC, reinforcing that this gene is unlikely to contribute meaningfully to ancestry-specific molecular divergence within this clinical setting. Importantly, this analysis illustrates the capability of the conversational AI system to rapidly generate ancestry-matched cohorts and validate mutation frequency comparisons, even in the context of sparsely mutated genes.

#### 3.7.5. AI-Driven Identification of Mutation-Level Disparities Within MAPK-Altered Early-Onset Tumors Across Ancestry Groups

To further characterize molecular variation linked to MAPK pathway dysregulation, we used the conversational AI system to compare mutation attributes between MAPK-altered early-onset H/L and NHW colorectal tumors (Figure S5). The platform autonomously generated ancestry-stratified cohorts based on integrated clinical-genomic criteria, resulting in 122 MAPK-altered H/L tumors and 652 MAPK-altered NHW tumors. Panels (a) and (b) illustrate cohort distribution patterns relative to the full data space, demonstrating a markedly larger representation of MAPK-altered cases among NHW patients.

The AI then performed exploratory, attribute-level prioritization to identify statistically significant differences between groups (panel c). The mutation profile comparison revealed broad ancestry-associated heterogeneity, with several canonical colorectal cancer drivers enriched at different rates across the two populations. Highly significant differences (*p* < 0.001) were observed for *TP53, APC, KRAS, TCF7L2*, and *PIK3CA*, followed by additional signals involving *SMAD4, BRAF, PIK3R1*, and *NF1*. Many of these genes intersect biologically with MAPK signaling or represent upstream regulators of pathway activation, underscoring their mechanistic relevance.

These exploratory findings suggest that MAPK-altered early-onset CRC is not molecularly uniform across ancestry groups, but instead reflects distinct co-mutation landscapes. The rapid attribute extraction made possible by conversational AI enabled the identification of multiple genomic features with potential relevance to ancestry-specific CRC biology. These results informed subsequent focused analyses and highlight the utility of AI-assisted interrogation for uncovering context-dependent molecular variation within MAPK-driven disease.

## 4. Discussion

This study represents, to our knowledge, one of the first conversational AI-enabled, pathway-focused analyses of the MAPK signaling axis in colorectal cancer systematically stratified by age at diagnosis, ancestry, and FOLFOX exposure, with an explicit emphasis on H/L populations. Using the AI-HOPE framework and its pathway-specific module AI-HOPE-MAPK, we harmonized multi-cohort clinical and genomic data from 2515 CRC cases and interrogated MAPK alterations through natural language-driven analytics combined with conventional biostatistical testing. This integrative approach allowed us to move beyond single-gene biomarkers and to examine how MAPK pathway disruption and co-mutation patterns vary across clinically and demographically relevant contexts.

### 4.1. Summary of Key Findings

First, MAPK pathway involvement was nearly universal across CRC, independent of ancestry, age of onset, or FOLFOX treatment. Pathway-level alterations were detected in ~96–100% of tumors in every subgroup examined, including EOCRC and LOCRC, H/L and NHW patients, and both FOLFOX-treated and non-FOLFOX-treated cohorts. This ubiquity underscores the central role of MAPK signaling in colorectal tumorigenesis but also indicates that a simple “MAPK-altered vs. not altered” status is not a useful discriminator for patient stratification.

Second, substantial heterogeneity emerged at the gene level, revealing context-dependent enrichment of specific MAPK components. In EO H/L tumors not exposed to FOLFOX, *NF1*, *FGFR2*, and the downstream effector *RPS6KA4* were significantly enriched compared with treated EO H/L cases. LO H/L tumors not treated with FOLFOX showed higher frequencies of *NTRK2* and *PDGFRB* mutations than their treated counterparts. Within the NHW population, multiple MAPK-related genes, including *AKT3, FGF4, JUN, RRAS2* in EOCRC and *CRKL, DUSP4, MAPK1, RAF1, RPS6KA4, RRAS,* and *SOS1* in LOCRC, displayed higher prevalence in non-FOLFOX-treated tumors, while JUN showed the opposite pattern in some strata. Age-stratified comparisons further highlighted *BRAF* enrichment in late-onset disease across ancestries and reinforced *NF1* enrichment in early-onset tumors, particularly in untreated H/L cases.

Third, ancestry-specific differences were most pronounced in early-onset, chemo-naïve disease. Among EOCRC patients not treated with FOLFOX, H/L tumors showed higher frequencies of *MAPK3, NF1*, and *RPS6KA4* mutations than NHW tumors, whereas among FOLFOX-treated EOCRC cases, *PDGFRB* alterations were more common in H/L than NHW tumors. These findings indicate that, although MAPK pathway disruption is pervasive in both populations, the specific nodes through which the pathway is perturbed differ by ancestry and treatment context.

Fourth, pathway-level MAPK mutation status was generally not prognostic for overall survival. Across eight Kaplan–Meier analyses stratified by ancestry, age, and FOLFOX exposure, we observed no statistically significant survival differences between MAPK-altered and non-altered groups. Small non-altered subgroups and broad confidence intervals limited power for some comparisons, and EOCRC NHW patients treated with FOLFOX exhibited a modest, non-significant trend toward worse survival in the MAPK-non-altered group. Overall, however, MAPK alteration status did not emerge as a robust survival biomarker in any stratum.

Finally, the conversational AI framework proved valuable as an exploratory engine. AI-guided analyses rapidly identified an ancestry-based survival advantage for FOLFOX-treated EO H/L patients compared with ancestry-matched NHW controls, confirmed that MSI stability and *AKT3* mutation frequency did not differ meaningfully by treatment in select subgroups, and surfaced mutation-level disparities within MAPK-altered early-onset tumors (e.g., differential *TP53, APC, KRAS, TCF7L2, PIK3CA*, and *NF1* frequencies between H/L and NHW cases). These AI-derived signals shaped our subsequent hypothesis-driven analyses.

### 4.2. Biological Implications of MAPK Pathway Alterations

The near-universal MAPK pathway alteration burden across CRC reinforces the concept that MAPK signaling represents a core dependency of colorectal tumorigenesis, spanning both canonical oncogenic drivers (*KRAS, BRAF, NRAS*) and a network of upstream receptors, adaptors, negative regulators, and effector kinases. However, the non-uniform distribution of individual gene alterations suggests that MAPK dysregulation is achieved through distinct molecular routes in different clinical contexts.

The enrichment of NF1 mutations in EO H/L tumors, especially in chemo-naïve cases, highlights the potential importance of disrupted negative regulation of *RAS* signaling in early-onset disease within this population. *NF1* loss is known to augment *RAS* activity, and its co-occurrence with other MAPK drivers could amplify pathway signaling or modify sensitivity to targeted agents. Similarly, *FGFR2, NTRK2, PDGFRB*, and *CRKL/SOS1* alterations implicate diverse receptor tyrosine kinases and adaptor proteins as alternative entry points into MAPK activation, particularly in older or untreated patients.

The age-associated increase in BRAF mutations, most prominent in LOCRC across ancestries, aligns with prior observations that *BRAF*-mutant CRC often presents at older ages and carries a distinct clinical phenotype. In our data, *BRAF* enrichment with age occurred against a background of stable *KRAS* and *TP53* frequencies, suggesting that *BRAF*-driven MAPK activation represents one of several age-linked evolutionary trajectories rather than a universal feature of late-onset disease.

The context-specific enrichment of *RPS6KA4* and *MAPK3*, both downstream effectors of ERK signaling, points toward variation not only in initiating events but also in terminal pathway branches. These genes may modulate transcriptional programs related to proliferation, stress responses, and chemoresistance. In EO H/L non-FOLFOX-treated tumors, co-occurrence of *NF1, RPS6KA4*, and *MAPK3* alterations suggests a unique MAPK architecture characterized by dysregulated negative feedback and enhanced effector signaling.

These patterns support a model in which MAPK activation is a shared requirement across CRC, but the wiring diagram of the pathway, where and how it is perturbed, varies with age, ancestry, and treatment history. Such variation may have implications for response to targeted inhibitors and for combination strategies that seek to exploit specific vulnerabilities within MAPK signaling networks.

Importantly, our findings intersect with emerging therapeutic insights into adaptive resistance mechanisms downstream of MAPK signaling, particularly involving the Hippo/YAP–TEAD axis. Recent genome-wide CRISPR screening studies have demonstrated that resistance to *KRAS*^G12C inhibitors is associated with adaptive vulnerabilities in *YAP/TAZ/TEAD* signaling, identifying *TEAD* inhibition as a promising therapeutic strategy [41]. Complementing this, growing evidence in colorectal cancer highlights the context-dependent oncogenic and tumor-suppressive roles of Hippo/*YAP* signaling, including its crosstalk with MAPK and WNT pathways, epigenetic regulation, immune modulation, and therapy resistance [42]. Within this framework, MAPK alterations identified in our study, particularly those involving regulatory and feedback nodes such as *NF1* and *RPS6KA* family members, may reflect broader network-level adaptations rather than isolated driver events, reinforcing the need for systems-level, pathway-integrated biomarker strategies.

### 4.3. Ancestry-Specific Genomic Patterns and Treatment Context

Our analyses underscore the importance of considering ancestry and treatment context together when interrogating MAPK biology. The most striking ancestry-associated differences emerged in early-onset, chemo-naïve disease, where H/L tumors showed higher frequencies of *NF1, MAPK3*, and *RPS6KA4* alterations than NHW tumors despite similar global mutation burdens. These findings suggest that early tumorigenic events in H/L individuals may preferentially involve deregulation of MAPK negative regulators and distal effectors, potentially reflecting differences in germline backgrounds, environmental exposures, or microenvironmental pressures.

In contrast, ancestry differences were attenuated among FOLFOX-treated EOCRC, where only *PDGFRB* exhibited significantly higher prevalence in H/L tumors. This pattern may indicate that chemotherapy exposure homogenizes observable MAPK architectures by applying selective pressure across ancestries or, alternatively, that treatment decisions introduce clinical selection biases that obscure underlying biological differences.

Among LOCRC, treatment context appeared to play a larger role than ancestry itself. Numerous MAPK-related genes showed higher mutation frequencies in non-FOLFOX-treated NHW tumors compared with treated counterparts, suggesting that either tumors with more complex MAPK architectures are less likely to receive FOLFOX or that such tumors arise in clinical contexts (e.g., comorbidities, performance status) that preclude intensive chemotherapy. H/L LOCRC tumors also exhibited treatment-linked enrichment of *NTRK2* and *PDGFRB* in non-FOLFOX cases, consistent with a broader theme that chemo-naïve tumors carry a richer diversity of MAPK entry points and modulators.

The translational relevance of this work is strengthened by its potential to inform therapeutic stratification and biomarker development in colorectal cancer, particularly in the context of treatment resistance. The context-dependent enrichment of specific MAPK-associated genes across ancestry, age, and FOLFOX exposure suggests that pathway composition, rather than pathway presence alone, may be critical for guiding therapeutic decision-making. These findings support the development of composite, context-aware biomarkers that integrate MAPK alterations with clinical variables and co-activated signaling pathways to better predict treatment response. Moreover, emerging evidence of adaptive resistance mechanisms, such as crosstalk between MAPK signaling and the Hippo/*YAP–TEAD* axis, highlights opportunities for rational combination strategies that may overcome resistance to targeted therapies. By prioritizing specific molecular contexts and patient subgroups, this study provides a framework to guide future translational efforts, including the design of ancestry-inclusive clinical trials and the evaluation of novel therapeutic combinations in precision oncology.

Taken together, these results support the view that ancestry-specific MAPK patterns are most apparent when examined within narrowly defined clinical strata, particularly early-onset, untreated disease. They also emphasize that real-world treatment patterns and selection effects must be carefully considered when interpreting apparent genomic differences between populations.

### 4.4. Implications for FOLFOX Response and Prognostic Stratification

Contrary to expectations based on preclinical data linking MAPK activation to chemoresistance and poor outcomes, we found no consistent association between MAPK pathway mutation status and overall survival in any age-, ancestry-, or treatment-defined subgroup. The lack of prognostic impact at the pathway level likely reflects several factors.

First, because MAPK pathway alterations are almost universal, a simple binary classification (altered vs. not altered) compresses the diversity of MAPK architectures into a coarse metric with limited discriminatory power. Second, the functional impact of individual mutations varies widely: many alterations may be passenger events or may even attenuate signaling, while others drive strong activation. Third, survival outcomes are shaped by interactions between MAPK and other oncogenic pathways (*WNT, PI3K, TGF-β*), tumor microenvironment features, and treatment regimens beyond FOLFOX, none of which are fully captured by a single pathway-level variable.

The modest, non-significant trend toward poorer outcomes in MAPK-non-altered EOCRC NHW patients treated with FOLFOX hints that tumors lacking canonical MAPK alterations may rely on alternative resistance mechanisms, but our data are underpowered to draw firm conclusions. In aggregate, these findings suggest that MAPK mutation status alone is unlikely to serve as a stand-alone prognostic marker for FOLFOX response. Instead, future work should explore multi-gene or co-mutation signatures, potentially integrating *NF1, BRAF, PDGFRB, RPS6KA4*, and other nodes with MSI, TMB, and clinical features to construct more nuanced models of chemotherapy sensitivity.

Several biologically plausible hypotheses may explain why specific MAPK-associated alterations are enriched within distinct demographic and treatment contexts. Differences by ancestry may reflect variation in tumor evolutionary pressures shaped by germline background, environmental exposures, and social determinants of health, which together influence somatic selection during tumor development. Age of onset may further stratify tumors by underlying biology, with early-onset colorectal cancers potentially favoring alterations in regulatory or feedback components of the MAPK pathway, such as *NF1* or *RPS6KA* family members, rather than canonical upstream drivers. In addition, exposure to cytotoxic chemotherapy, including FOLFOX, may impose selective pressures that promote adaptive signaling rewiring, enriching for alterations in receptor-level or regulatory nodes such as *PDGFRB*, consistent with compensatory pathway activation rather than primary oncogenic signaling. These interpretations remain speculative and hypothesis-generating; however, they provide a biologically grounded framework to guide future functional and longitudinal studies aimed at dissecting the mechanisms underlying context-dependent MAPK alterations in colorectal cancer.

Importantly, our conversational AI analyses also showed that ancestry itself, independent of MAPK status, can be associated with survival differences in specific contexts, as illustrated by the better outcomes observed in EO H/L vs. EO NHW patients treated with FOLFOX. These findings reinforce that pathway-focused biomarkers must ultimately be interpreted alongside social, environmental, and systemic determinants of health to avoid oversimplified conclusions about biology-driven disparities.

### 4.5. AI-HOPE-MAPK as an Enabling Technology

The AI-HOPE and AI-HOPE-MAPK platforms were central to this study. By embedding harmonized clinical, genomic, ancestry, and treatment data within a conversational analytic environment, the system allowed investigators to pose complex questions, such as “compare MAPK-altered early-onset H/L tumors not treated with FOLFOX to MAPK-altered NHW counterparts and identify genes that differ significantly”, using natural language rather than bespoke code.

This capability yielded several advantages: (a) Rapid hypothesis generation: AI-guided queries quickly surfaced context-specific signals (e.g., *NF1* and *RPS6KA4* enrichment in EO H/L non-FOLFOX tumors, *BRAF* enrichment in LO disease, ancestry-linked co-mutation differences in MAPK-altered EOCRC) that would have been laborious to identify manually. (b) Efficient validation of null results: The platform confirmed that MSI stability, *AKT3* mutation frequency, and *PDGFRB* mutation prevalence did not differ meaningfully between select treatment or ancestry strata, allowing us to de-prioritize these hypotheses early. (c) Transparent cohort construction: AI-generated cohort definitions and 2 × 2 tables were cross-checked against conventional R scripts and statistical tests, reducing manual data wrangling and minimizing transcription errors. (d) Equity-focused analytics: By design, the system facilitated ancestry-aware and age-aware stratification, ensuring that H/L populations, often underrepresented in genomic datasets, were interrogated with the same granularity as NHW populations.

In this study, conversational AI served as an enabling infrastructure rather than an inferential engine, accelerating complex, multi-parameter analyses that would otherwise be time- and resource-intensive. By orchestrating cohort construction and exploratory querying across ancestry, age, treatment, and pathway-level genomic features, the AI framework facilitated rapid hypothesis generation while preserving analytical rigor. Critically, all findings were grounded in conventional statistical validation frameworks, ensuring transparency, reproducibility, and interpretability. This hybrid approach highlights how conversational AI can augment precision oncology research by lowering analytical barriers and increasing scalability, while maintaining strict reliance on established statistical methodologies for inference and reporting.

Crucially, we treated AI-HOPE-MAPK as an analytic accelerator rather than a replacement for traditional statistics. All AI-suggested findings were validated using standard methods, and several AI-flagged trends did not withstand formal testing, highlighting the importance of coupling exploratory AI with rigorous biostatistical oversight.

### 4.6. Limitations and Future Directions

This study has several limitations. Despite the large overall cohort, many clinically important subgroups, especially H/L patients lacking FOLFOX exposure or carrying specific MAPK alterations, remained relatively small, leading to wide confidence intervals and limited power for survival and gene-frequency comparisons. Our analyses were restricted to coding single-nucleotide variants and small indels; copy-number alterations, structural variants, gene fusions (particularly relevant for *NTRK* genes), and expression or phosphoproteomic readouts of MAPK activation were not systematically incorporated.

Treatment annotation, while sufficient to classify FOLFOX exposure, lacked granular detail on dose intensity, treatment duration, combination partners, and subsequent lines of therapy, all of which can modulate outcomes and interact with MAPK biology. The retrospective, multi-cohort design introduces potential confounding from unmeasured variables such as comorbidities, performance status, socioeconomic factors, and center-specific treatment practices, factors that are particularly salient when interpreting ancestry-linked differences.

From a methodological standpoint, ancestry assignment relied on a combination of self-reported ethnicity and surname-based inference, which may misclassify some individuals and does not capture intra-group diversity (e.g., Caribbean vs. Mexican heritage). Likewise, AI-HOPE-MAPK depends on the quality and completeness of source annotations; systematic biases or errors in the underlying datasets can propagate through AI-assisted analyses. Future iterations of the platform should incorporate data provenance tracking, automated uncertainty flagging, and fairness auditing to ensure that AI workflows do not inadvertently perpetuate existing inequities.

This study should be interpreted within the context of its exploratory and hypothesis-generating design. While MAPK pathway alterations were highly prevalent across all colorectal cancer subgroups analyzed, the central contribution of this work lies not in overall pathway frequency, but in the context-dependent distribution of specific MAPK-associated gene alterations across ancestry, age of onset, and FOLFOX treatment exposure. Leveraging one of the few harmonized datasets with integrated clinical, genomic, treatment, and ancestry information, particularly inclusive of Hispanic/Latino patients, enabled a precision oncology- and cancer disparities-focused analysis that would not otherwise be feasible. The broad MAPK gene set used in this study was intentionally selected to maximize sensitivity for discovery in underrepresented populations; however, this approach may limit immediate clinical actionability and attenuate effect sizes. Accordingly, our findings should be viewed as generating testable hypotheses rather than definitive biomarkers. Future prospective studies and validation efforts using more restricted, clinically actionable MAPK gene panels, as well as functional and treatment-response analyses, will be essential to refine these observations and translate them into clinically meaningful precision oncology strategies.

Several limitations of this study warrant consideration. First, this analysis is entirely computational and retrospective, relying on harmonized public datasets rather than experimental model systems or prospective clinical cohorts. As such, no functional validation was performed to directly test drug sensitivity or mechanistic consequences of prioritized MAPK-associated alterations, including *NF1*, *RPS6KA4*, and *PDGFRB*. However, this design reflects both the current limitations of available experimental models and the scarcity of prospective datasets that adequately represent historically underrepresented populations, particularly Hispanic/Latino patients, within precision oncology research. A primary strength and motivation of this work is its focus on addressing this gap by systematically characterizing context-dependent molecular alterations in a large, ancestry-annotated cohort. Accordingly, the findings should be interpreted as hypothesis-generating, serving to prioritize candidate genes and clinical contexts for future validation. Prospective studies incorporating cell lines, patient-derived organoids, and treatment-response assays—ideally informed by ancestry and treatment exposure—will be essential to establish causality, define underlying mechanisms, and translate these observations into clinically actionable precision oncology strategies.

The prognostic analyses presented in this study should be interpreted within the context of the available data and the exploratory goals of the investigation. Although MAPK pathway status did not consistently demonstrate statistically significant associations with overall survival across all subgroups, this finding likely reflects limitations of the underlying data source. Specifically, our analyses rely on cBioPortal, one of the few publicly available platforms that integrates genomic and clinical data at scale for historically underrepresented populations, including Hispanic/Latino patients; however, the depth of clinical annotation is limited, constraining the development and validation of more complex, multivariable prognostic models. Importantly, the primary objective of this work was to characterize context-dependent patterns of MAPK pathway alterations across ancestry, age, and treatment exposure, rather than to establish definitive prognostic biomarkers. Accordingly, the survival analyses should be viewed as exploratory and hypothesis-generating, and future studies incorporating richer clinical data and prospective designs will be essential to develop and validate composite biomarkers that integrate MAPK alterations with clinical features to support clinically actionable precision oncology strategies.

The survival analyses in this study should be interpreted within the context of the available data and the exploratory goals of the investigation. Using cBioPortal, one of the few publicly accessible resources that integrates genomic and clinical information for historically underrepresented populations, including Hispanic/Latino patients, inevitably imposes limitations related to the depth and completeness of clinical annotation. While MAPK pathway status alone did not consistently demonstrate strong prognostic value across all subgroups, this does not negate its biological or clinical relevance. Rather, it highlights the complexity of MAPK signaling and the likelihood that single-pathway or single-gene metrics are insufficient to capture outcome-relevant heterogeneity in colorectal cancer. The absence of robust survival associations underscores the need for composite biomarker strategies that integrate multiple MAPK-related alterations with key clinical variables, treatment context, and additional molecular features. Accordingly, our findings should be viewed as hypothesis-generating and as a foundation for future studies leveraging richer clinical data and prospective cohorts to develop clinically actionable, context-aware precision oncology biomarkers.

The clinical relevance of this study lies in refining how MAPK pathway information is interpreted and contextualized, rather than in proposing immediate changes to treatment strategies based on pathway status alone. Although MAPK alterations did not consistently predict survival outcomes, our findings demonstrate that MAPK signaling is not a uniform entity in colorectal cancer, with the specific genes involved varying substantially by ancestry, age of onset, and FOLFOX exposure. This has important implications for clinical practice, as molecular results derived predominantly from non-Hispanic White or treatment-naïve populations may not be directly generalizable to diverse patient groups, particularly Hispanic/Latino patients. By highlighting context-dependent molecular heterogeneity, this work supports a shift toward ancestry-aware and treatment-informed interpretation of genomic reports, prioritizes specific MAPK-associated genes and subgroups for future validation, and underscores the need for composite biomarkers that integrate molecular alterations with clinical features. Accordingly, these results should be viewed as hypothesis-generating and as a framework to guide future prospective and translational studies aimed at developing clinically actionable precision oncology strategies.

Given the retrospective and computational nature of this study, it is important to distinguish clearly between correlative associations and causal mechanisms. The analyses presented here identify context-dependent patterns of MAPK pathway alterations across ancestry, age, and treatment exposure, but they do not establish direct mechanistic links to tumor progression or therapy response. Where possible, our interpretations are supported by prior functional evidence from the literature describing the roles of MAPK-associated genes in colorectal cancer biology; however, the findings should be viewed as associative and hypothesis-generating rather than causal. By explicitly acknowledging this distinction, we aim to avoid overinterpretation while highlighting how these results prioritize biologically plausible genes and clinical contexts for future investigation. Prospective studies incorporating functional assays, model systems, and treatment-response experiments will be essential to validate causality and translate these observations into actionable precision oncology strategies.

An additional limitation of this study relates to the definition of FOLFOX exposure. Due to the structure of available clinical annotations in large public datasets, patients were classified as FOLFOX-treated based on documented receipt of folinic acid, fluorouracil, and oxaliplatin within the same line of therapy, without confirmation that these agents were administered concurrently as a standardized FOLFOX regimen. This may introduce treatment heterogeneity related to sequencing, dosing, or schedule variation. While this approach is consistent with prior large-scale retrospective analyses and reflects real-world treatment patterns, it underscores the exploratory nature of our findings. Accordingly, the results should be interpreted cautiously, and future studies with more granular treatment data will be essential to validate these observations and refine treatment-specific biomarker associations.

Ancestry assignment represents an important consideration in the interpretation of this study. Hispanic/Latino classification was based on the ancestry annotations available in the contributing datasets, which primarily rely on self-reported ethnicity and, when unavailable, surname-based inference. While surname-based approaches have been widely used in epidemiologic and cancer disparities research and demonstrate reasonable concordance at the population level, they are an imperfect proxy and do not capture the complexity of genetic ancestry in admixed populations. Direct validation using germline ancestry-informative markers was not feasible given the retrospective nature of the data. Accordingly, ancestry in this study should be interpreted as a sociodemographic construct rather than a measure of genetic ancestry, and the findings are intended to highlight population-level patterns rather than individual-level inference. Future studies incorporating germline genotyping and more granular ancestry measures will be essential to refine these observations and to disentangle biological ancestry from social and structural determinants of cancer outcomes.

Future research should pursue several directions. First, prospective, ancestrally diverse cohorts with standardized treatment and biospecimen collection will be crucial to validate the gene-level MAPK signals identified here, particularly *NF1, PDGFRB, MAPK3, RPS6KA4, NTRK2*, and *RAF1/SOS1/CRKL*. Second, integrating multi-omic measures of pathway activity, including RNA expression signatures, phospho-proteomics, and spatial profiling, with mutational data may yield more predictive MAPK activation scores than genomic status alone. Third, mechanistic studies using in vitro and in vivo models representative of H/L and NHW genetic backgrounds are needed to elucidate how specific MAPK alterations interact with chemotherapy and with other oncogenic pathways. Finally, extension of AI-HOPE-MAPK to model crosstalk between MAPK and WNT, PI3K, TGF-β, and immune pathways could uncover composite biomarkers that better capture the complexity of CRC biology.

## 5. Conclusions

In this ancestry-, age-, and treatment-stratified colorectal cancer cohort, we demonstrate that while MAPK pathway alterations are highly prevalent, the specific genes and regulatory components involved differ in a context-dependent manner rather than uniformly across all patients. Within this defined population, early-onset, chemo-naïve Hispanic/Latino tumors were enriched for alterations in *NF1, MAPK3*, and *RPS6KA4*, whereas late-onset and non-FOLFOX-treated non-Hispanic White tumors exhibited distinct constellations of MAPK modulators. In addition, BRAF mutation burden increased with age across both ancestry groups. These findings underscore that MAPK signaling heterogeneity in colorectal cancer is strongly shaped by ancestry, age of onset, and treatment exposure within the studied cohort.

Despite this gene-level heterogeneity, MAPK pathway alteration status alone did not consistently predict overall survival across the analyzed subgroups. This observation highlights the limitations of binary pathway-based metrics in retrospective datasets and emphasizes the need for composite, context-aware biomarkers that integrate molecular features with clinical and treatment variables, particularly in underrepresented populations.

Beyond the biological findings, this study illustrates how conversational AI frameworks, such as AI-HOPE-MAPK, can support precision oncology research by harmonizing complex clinical-genomic data, accelerating hypothesis generation, and enabling ancestry-aware analyses. Importantly, conversational AI functioned as an analytic accelerator and orchestration layer, while all reported findings were validated using standard statistical methods.

Taken together, these results should be interpreted as hypothesis-generating within the specific colorectal cancer cohort studied. Future efforts incorporating mechanistic validation, prospective clinical annotation, and more granular treatment data in diverse populations will be essential to translate these MAPK-focused insights into clinically actionable precision oncology strategies and to ensure equitable advancement of cancer genomics research.

## Figures and Tables

**Table 1:** Baseline Demographic, Clinical, and Molecular Characteristics of the Study Cohort.

Category	Variable	Hispanic/Latino (H/L) n = 266	Non-Hispanic White (NHW) n = 2249
Age at Diagnosis & FOLFOX Exposure	EOCRC, FOLFOX-treated	73 (27.4%)	375 (16.7%)
	EOCRC, not FOLFOX-treated	52 (19.5%)	302 (13.4%)
	LOCRC, FOLFOX-treated	91 (34.2%)	919 (40.9%)
	LOCRC, not FOLFOX-treated	50 (18.8%)	653 (29.0%)
Tumor Site	Colon adenocarcinoma	164 (61.7%)	1328 (59.0%)
	Rectal adenocarcinoma	64 (24.1%)	646 (28.7%)
	Colorectal adenocarcinoma (NOS)	38 (14.3%)	275 (12.2%)
Sex	Male	158 (59.4%)	1267 (56.3%)
	Female	108 (40.6%)	982 (43.7%)
Tumor Sample Source	Primary tumor	266 (100%)	2249 (100%)
Clinical Stage at Diagnosis	Stage I–III	156 (58.6%)	1236 (55.0%)
	Stage IV	108 (40.6%)	1005 (44.7%)
	Missing	2 (0.8%)	8 (0.4%)
Microsatellite Status	MSS	200 (75.2%)	1940 (86.3%)
	MSI-H	21 (7.9%)	209 (9.3%)
	Indeterminate	10 (3.8%)	57 (2.5%)
	Missing	35 (13.2%)	43 (1.9%)
Ethnicity Annotation	Spanish/Hispanic NOS	230 (86.5%)	—
	Mexican/Chicano	30 (11.3%)	—
	Other Hispanic categories *	6 (2.3%)	—
	Non-Hispanic White	—	2249 (100%)

* Includes “Hispanic or Latino,” “Other Spanish/Hispanic,” and surname-inferred Hispanic ancestry.

**Table 2:** Age-, Ancestry-, and Treatment-Stratified Clinical and MAPK Genomic Features in Colorectal Cancer. This table summarizes key clinical parameters and MAPK pathway-related genomic characteristics across colorectal cancer subgroups defined by age at diagnosis, ancestry, and FOLFOX treatment exposure. Analyses are presented across four complementary comparisons: (**a**) early-onset versus late-onset disease within Hispanic/Latino (H/L) patients; (**b**) early-onset versus late-onset disease within non-Hispanic White (NHW) patients; and (**c**) ancestry-based comparisons among early-onset colorectal cancer (EOCRC) cases. Reported variables include median age at diagnosis, overall mutation burden, TMB, fraction of genome altered, and the frequency of selected MAPK signaling pathway gene alterations.

(**a**)						
**Clinical Feature**	**Early-Onset Hispanic/Latino** **Treated with FOLFOX** **n (%)**	**Early-Onset Hispanic/Latino** **Not Treated with FOLFOX** **n (%)**	***p*** **-Value**	**Late-Onset Hispanic/Latino** **Treated with FOLFOX** **n (%)**	**Late-Onset Hispanic/Latino** **Not Treated with FOLFOX** **n (%)**	***p*** **-Value**
Median Diagnosis Age (IQR)	42 (36–47)	40 (34–43)	0.05411	59 (54–66)	62 (56–70)	0.04865
Median Mutation Count	7 (5–8)	7 (5–20)	0.09735	8 (6–9) [NA = 1]	7 (5.25–9)	0.6507
Median TMB (IQR)	6.3 (4.5–7.8) [NA = 15]	5.5 (3.4–8.3) [NA = 2]	0.1719	6.1 (4.9–7.8) [NA = 10]	6.9 (5.6–9.0) [NA = 2]	0.04389
Median FGA	0.18 (0.03–0.27) [NA = 6]	0.19 (0.03–0.29)	0.7661	0.15 (0.06–0.25) [NA = 7]	0.21 (0.04–0.3) [NA = 2]	0.5464
FGFR2 Mutation						
Present	0 (0.0%)	4 (7.7%)	0.02793	0 (0.0%)	1 (2.0%)	0.3546
Absent	73 (100.0%)	48 (92.3%)		91 (100.0%)	49 (98.0%)	
NF1 Mutation						
Present	3 (4.1%)	10 (19.2%)	0.01437	2 (2.2%)	2 (4.0%)	0.615
Absent	70 (95.9%)	42 (80.8%)		89 (97.8%)	48 (96.0%)	
NTRK2 Mutation						
Present	1 (1.4%)	2 (3.8%)	0.5699	0 (0.0%)	3 (6.0%)	0.04286
Absent	72 (98.6%)	50 (96.2%)		91 (100.0%)	47 (94.0%)	
PDGFRB Mutation						
Present	4 (5.5%)	3 (5.8%)	1	0 (0.0%)	3 (6.0%)	0.04286
Absent	69 (94.5%)	49 (94.2%)		91 (100.0%)	47 (94.0%)	
RPS6KA4 Mutation						
Present	0 (0.0%)	5 (9.6%)	0.01108	0 (0.0%)	3 (6.0%)	0.6659
Absent	73 (100.0%)	47 (90.4%)		91 (100.0%)	47 (94.0%)	
(**b**)						
**Clinical Feature**	**Early-Onset NHW** **Treated with FOLFOX** **n (%)**	**Early-Onset NHW** **Not Treated with FOLFOX** **n (%)**	***p*** **-Value**	**Late-Onset NHW** **Treated with FOLFOX** **n (%)**	**Late-Onset NHW** **Not Treated with FOLFOX** **n (%)**	***p*** **-Value**
Median Diagnosis Age (IQR)	43 (37–48)	44 (38–47)	0.5646	63 (57–69)	66 (57–74)	4.146 × 10^−7^
Median Mutation Count	6 (5–8) [NA = 4]	7 (5–9) [NA = 2]	0.1258	7 (5–9) [NA = 10]	8 (6–12) [NA = 3]	1.22 × 10^−5^
Median TMB (IQR)	5.7 (4.1–6.9)	5.7 (4.1–7.8)	0.4214	6.1 (4.3–8.2)	6.6 (4.9–10.4)	0.0002854
Median FGA	0.14 (0.04–0.24) [NA = 4]	0.15 (0.04–0.23) [NA = 2]	0.5589	0.16 (0.06–0.28) [NA = 6]	0.15 (0.05–0.27) [NA = 5]	0.1929
AKT3 Mutation						
Present	3 (0.8%)	9 (3.0%)	0.04069	9 (1.0%)	12 (1.8%)	0.2158
Absent	372 (99.2%)	293 (97.0%)		910 (99.0%)	641 (98.2%)	
CRKL Mutation						
Present	1 (0.3%)	3 (1.0%)	0.3291	1 (0.1%)	8 (1.2%)	0.004928
Absent	374 (99.7%)	299 (99.0%)		918 (99.9%)	645 (98.8%)	
DUSP4 Mutation						
Present	1 (0.3%)	1 (0.3%)	1	1 (0.1%)	6 (0.9%)	0.02292
Absent	374 (99.7%)	301 (99.7%)		918 (99.9%)	647 (99.1%)	
FGF4 Mutation						
Present	0 (0.0%)	5 (1.7%)	0.01734	1 (0.1%)	4 (0.6%)	0.167
Absent	375 (100.0%)	297 (98.3%)		918 (99.9%)	649 (99.4%)	
JUN Mutation						
Present	12 (3.2%)	2 (0.7%)	0.02726	13 (1.4%)	21 (3.2%)	0.02486
Absent	363 (96.8%)	300 (99.3%)		906 (98.6%)	632 (96.8%)	
MAPK1 Mutation						
Present	3 (0.8%)	3 (1.0%)	1	1 (0.1%)	6 (0.9%)	0.02292
Absent	372 (99.2%)	299 (99.0%)		918 (99.9%)	647 (99.1%)	
RRAS Mutation						
Present	4 (1.1%)	0 (0.0%)	0.1326	2 (0.2%)	8 (1.2%)	0.02024
Absent	371 (98.9%)	302 (100.0%)		917 (99.8%)	645 (98.8%)	
RRAS2 Mutation						
Present	0 (0.0%)	4 (1.3%)	0.03916	5 (0.5%)	3 (0.5%)	1
Absent	375 (100.0%)	298 (98.7%)		914 (99.5%)	650 (99.5%)	
SOS1 Mutation						
Present	7 (1.9%)	11 (3.6%)	0.2351	8 (0.9%)	15 (2.3%)	0.03501
Absent	368 (98.1%)	291 (96.4%)		911 (99.1%)	638 (97.7%)	
(**c**)						
**Clinical Feature**	**Early-Onset Hispanic/Latino** **Treated with FOLFOX** **n (%)**	**Early-Onset NHW** **Treated with FOLFOX** **n (%)**	***p*** **-Value**	**Early-Onset Hispanic/Latino** **Not Treated with FOLFOX** **n (%)**	**Early-Onset NHW** **Not Treated with FOLFOX** **n (%)**	***p*** **-Value**
Median Diagnosis Age (IQR)	42 (36–47)	43 (37–48)	0.08467	40 (34–43)	44 (38–47)	0.0006016
Median Mutation Count	7 (5–8)	6 (5–8) [NA = 4]	0.942	7 (5–20)	7 (5–9) [NA = 2]	0.2601
Median TMB (IQR)	6.3 (4.5–7.8) [NA = 15]	5.7 (4.1–6.9)	0.05806	5.5 (3.4–8.3) [NA = 2]	5.7 (4.1–7.8)	0.5732
Median FGA	0.18 (0.03–0.27) [NA = 6]	0.14 (0.04–0.24) [NA = 4]	0.5556	0.19 (0.03–0.29)	0.15 (0.04–0.23) [NA = 2]	0.3612
MAPK3 Mutation						
Present	1 (1.4%)	1 (0.3%)	0.2996	3 (5.8%)	3 (1.0%)	0.04335
Absent	72 (98.6%)	374 (99.7%)		49 (94.2%)	299 (99.0%)	
NF1 Mutation						
Present	3 (4.1%)	17 (4.5%)	1	10 (19.2%)	18 (6.0%)	0.002728
Absent	70 (95.9%)	358 (95.5%)		42 (80.8%)	284 (94.0%)	
PDGFRB Mutation						
Present	4 (5.5%)	3 (0.8%)	0.01547	3 (5.8%)	7 (2.3%)	0.1693
Absent	69 (94.5%)	372 (99.2%)		49 (94.2%)	295 (97.7%)	
RPS6KA4 Mutation						
Present	0 (0.0%)	5 (1.3%)	1	5 (9.6%)	8 (2.6%)	0.03866
Absent	73 (100.0%)	370 (98.7%)		47 (90.4%)	294 (97.4%)	

**Table 3:** Prevalence of MAPK Pathway Alterations Across Age-, Ancestry-, and Treatment-Defined Colorectal Cancer Subgroups. This table summarizes the frequency of MAPK pathway alterations across colorectal cancer cohorts stratified by age at diagnosis (early-onset vs. late-onset), ancestry (Hispanic/Latino vs. non-Hispanic White), and FOLFOX chemotherapy exposure. Data are organized into four analytical panels: (**a**) within-ancestry comparisons of early- and late-onset disease among Hispanic/Latino patients by treatment status; (**b**) parallel age- and treatment-based comparisons within the non-Hispanic White cohort; (**c**) ancestry-based comparisons restricted to early-onset colorectal cancer, stratified by FOLFOX exposure; and (**d**) ancestry-based comparisons among late-onset colorectal cancer cases, also stratified by treatment status.

(**a**)						
**Pathway Alterations**	**Early-Onset Hispanic/Latino** **Treated with FOLFOX** **n (%)**	**Early-Onset Hispanic/Latino** **Not Treated with FOLFOX** **n (%)**	***p*** **-Value**	**Late-Onset Hispanic/Latino** **Treated with FOLFOX** **n (%)**	**Late-Onset Hispanic/Latino** **Not Treated with FOLFOX** **n (%)**	***p*** **-Value**
MAPK Alterations Present	70 (95.9%)	52 (100.0%)	0.2653	89 (97.8%)	50 (100.0%)	0.539
MAPK Alterations Absent	3 (4.1%)	0 (0.0%)		2 (2.2%)	0 (0.0%)	
(**b**)						
**Pathway Alterations**	**Early-Onset NHW** **Treated with FOLFOX** **n (%)**	**Early-Onset NHW** **Not Treated with FOLFOX** **n (%)**	***p*** **-Value**	**Late-Onset NHW** **Treated with FOLFOX** **n (%)**	**Late-Onset NHW** **Not Treated with FOLFOX** **n (%)**	***p*** **-Value**
MAPK Alterations Present	361 (96.3%)	291 (96.4%)	1	885 (96.3%)	629 (96.3%)	1
MAPK Alterations Absent	14 (3.7%)	11 (3.6%)		34 (3.7%)	24 (3.7%)	
(**c**)						
**Pathway Alterations**	**Early-Onset Hispanic/Latino** **Treated with FOLFOX** **n (%)**	**Early-Onset NHW** **Treated with FOLFOX** **n (%)**	***p*** **-Value**	**Early-Onset Hispanic/Latino** **Not Treated with FOLFOX** **n (%)**	**Early-Onset NHW** **Not Treated with FOLFOX** **n (%)**	***p*** **-Value**
MAPK Alterations Present	70 (95.9%)	361 (96.3%)	0.7469	52 (100.0%)	291 (96.4%)	0.379
MAPK Alterations Absent	3 (4.1%)	14 (3.7%)		0 (0.0%)	11 (3.6%)	
(**d**)						
**Pathway Alterations**	**Late-Onset Hispanic/Latino** **Treated with FOLFOX** **n (%)**	**Late-Onset NHW** **Treated with FOLFOX** **n (%)**	***p*** **-Value**	**Late-Onset Hispanic/Latino** **Not Treated with FOLFOX** **n (%)**	**Late-Onset NHW** **Not Treated with FOLFOX** **n (%)**	***p*** **-Value**
MAPK Alterations Present	89 (97.8%)	885 (96.3%)	0.7646	50 (100.0%)	629 (96.3%)	0.4049
MAPK Alterations Absent	2 (2.2%)	34 (3.7%)		0 (0.0%)	24 (3.7%)	

## Data Availability

All datasets used in this study are publicly accessible via cBioPortal at https://www.cbioportal.org and https://genie.cbioportal.org (both accessed on 11 October 2025). Analytical code and resources to support reproducibility are available at https://github.com/Velazquez-Villarreal-Lab/AI-MPK (accessed on 11 October 2025). Additional materials may be obtained from the authors upon reasonable request.

## Supplementary Materials

The following supporting information can be downloaded at: https://www.mdpi.com/article/10.3390/cancers18020293/s1, Figure S1: AI-directed cohort selection and survival comparison for early-onset treated His-panic/Latino (H/L) and Non-Hispanic White (NHW) colorectal cancer (CRC) patients; Figure S2: AI-driven analysis of MSI stability patterns among early-onset Hispanic/Latino col-orectal cancer patients stratified by FOLFOX treatment exposure; Figure S3: AI-guided assessment of AKT3 mutation frequency among early-onset Non-Hispanic White (NHW) colorectal cancer patients stratified by FOLFOX exposure; Figure S4: AI-supported evaluation of PDGFRB mutation prevalence in early-onset colorectal cancer patients treated with FOLFOX across Hispanic/Latino (H/L) and Non-Hispanic White (NHW) populations; Figure S5: AI-enabled comparison of MAPK-altered early-onset colorectal cancer tumors re-veals ancestry-linked molecular differences; Table S1: Comparison of Early-Onset Hispanic/Latino (H/L) Patients Treated with FOLFOX versus Not Treated with FOLFOX; Table S2: Comparison of Late-Onset Hispanic/Latino (H/L) Patients Treated with FOLFOX versus Not Treated with FOLFOX; Table S3: Comparison of Early-Onset Non-Hispanic White (NHW) Patients Treated with FOLFOX versus Not Treated with FOLFOX; Table S4: Comparison of Late-Onset Non-Hispanic White (NHW) Patients Treated with FOLFOX versus Not Treated with FOLFOX; Table S5: Comparison of Early-Onset versus Late-Onset Hispanic/Latino (H/L) Patients Treated with FOLFOX; Table S6: Comparison of Early-Onset versus Late-Onset Hispanic/Latino (H/L) Patients Not Treated with FOLFOX; Table S7: Comparison of Early-Onset versus Late-Onset Non-Hispanic White (NHW) Patients Treated with FOLFOX; Table S8: Comparison of Early-Onset Hispanic/Latino (H/L) versus Early-Onset Non-Hispanic White (NHW) Patients Treated with FOLFOX; Table S9: Comparison of Early-Onset Hispanic/Latino (H/L) versus Early-Onset Non-Hispanic White (NHW) Patients Not Treated with FOLFOX; Table S10: Comparison of Late-Onset Hispanic/Latino (H/L) versus Late-Onset Non-Hispanic White (NHW) Patients Treated with FOLFOX; Table S11: Comparison of Late-Onset Hispanic/Latino (H/L) versus Late-Onset Non-Hispanic White (NHW) Patients Not Treated with FOLFOX; Table S12: Comparison of Late-Onset Hispanic/Latino (H/L) versus Late-Onset Non-Hispanic White (NHW) Patients Not Treated with FOLFOX; Table S13: Distribution of MAPK Pathway Mutation Classes by Ancestry, Age at Diagnosis, and FOLFOX Exposure in Colorectal Cancer.
