# Supplementary material for: Conversational AI-Enabled Precision Oncology Reveals Context-Dependent MAPK Pathway Alterations in Hispanic/Latino and Non-Hispanic White Colorectal Cancer Stratified by Age and FOLFOX Exposure
Source: Cancers (Basel). 2026 Jan 17;18(2):293. doi: 10.3390/cancers18020293 (PMC12839066; doi:10.3390/cancers18020293)
Supplement: Supplementary file 1 [file cancers-18-00293-s001.zip › cancers-4081678-supplementary.pdf]

cancers

MDPI

Supplementary Materials: Conversational AI-Enabled Precision Oncology Reveals Context-Dependent MAPK Pathway Alterations in Hispanic/Latino and Non-Hispanic White Colorectal Cancer Stratified by Age and FOLFOX Exposure

Fernando C. Diaz [1], BrigetteWaldrup [2], Francisco G. Carranza [2], Sophia Manjarrez [2] and Enrique Velazquez-Villarreal [2,3,*]

[1] Lineberger Comprehensive Cancer Center, University of North Carolina, Chapel Hill, NC 27514, USA
[2] Department of Integrative Translational Sciences, Beckman Research Institute, City of Hope, Duarte, CA 91010, USA
[3] City of Hope Comprehensive Cancer Center, Duarte, CA 91010, USA
* Correspondence: evelazquezvilla@coh.org

Table S1. - Comparison of Early-Onset Hispanic/Latino (H/L) Patients Treated with FOLFOX versus Not Treated with FOLFOX.

MAPK Pathway			
Gene	Early-Onset Hispanic/Latino Treated with FOLFOX n (%)	Early-Onset Hispanic/Latino Not Treated with FOLFOX n (%)	p-value
**ACVR1 Mutation**			
Present	1 (1.4%)	2 (3.8%)	0.5699
Absent	72 (98.6%)	50 (96.2%)	
**AKT1 Mutation**			
Present	4 (5.5%)	2 (3.8%)	1
Absent	69 (94.5%)	50 (96.2%)	
**AKT2 Mutation**			
Present	0 (0.0%)	0 (0.0%)	1
Absent	73 (100.0%)	52 (100.0%)	
**AKT3 Mutation**			
Present	2 (2.7%)	3 (5.8%)	0.6484
Absent	71 (97.3%)	49 (94.2%)	
**BRAF Mutation**			
Present	3 (4.1%)	4 (7.7%)	0.4486
Absent	70 (95.9%)	48 (92.3%)	
**CACNA1H Mutation**			
Present	0 (0.0%)	0 (0.0%)	1
Absent	73 (100.0%)	52 (100.0%)	
**CACNA2D1 Mutation**			
Present	0 (0.0%)	0 (0.0%)	1
Absent	73 (100.0%)	52 (100.0%)	
**CACNB1 Mutation**			
Present	0 (0.0%)	0 (0.0%)	1
Absent	73 (100.0%)	52 (100.0%)	
**CDC42 Mutation**			
Present	0 (0.0%)	0 (0.0%)	1
Absent	73 (100.0%)	52 (100.0%)	

**CRKL Mutation**			
Present	0 (0.0%)	0 (0.0%)	1
Absent	73 (100.0%)	52 (100.0%)	
**DAXX Mutation**			
Present	1 (1.4%)	2 (3.8%)	0.5699
Absent	72 (98.6%)	50 (96.2%)	
**DUSP4 Mutation**			
Present	0 (0.0%)	0 (0.0%)	1
Absent	73 (100.0%)	52 (100.0%)	
**EGFR Mutation**			
Present	1 (1.4%)	3 (5.8%)	0.3067
Absent	72 (98.6%)	49 (94.2%)	
**FGF13 Mutation**			
Present	0 (0.0%)	0 (0.0%)	1
Absent	73 (100.0%)	52 (100.0%)	
**FGF19 Mutation**			
Present	1 (1.4%)	0 (0.0%)	1
Absent	72 (98.6%)	52 (100.0%)	
**FGF3 Mutation**			
Present	0 (0.0%)	1 (1.9%)	0.416
Absent	73 (100.0%)	51 (98.1%)	
**FGF4 Mutation**			
Present	0 (0.0%)	0 (0.0%)	1
Absent	73 (100.0%)	52 (100.0%)	
**FGFR1 Mutation**			
Present	0 (0.0%)	2 (3.8%)	0.1711
Absent	73 (100.0%)	50 (96.2%)	
**FGFR2 Mutation**			
Present	0 (0.0%)	4 (7.7%)	**0.02793**
Absent	73 (100.0%)	48 (92.3%)	
**FGFR3 Mutation**			
Present	1 (1.4%)	2 (3.8%)	0.5699
Absent	72 (98.6%)	50 (96.2%)	
**FGFR4 Mutation**			
Present	1 (1.4%)	1 (1.9%)	1
Absent	72 (98.6%)	51 (98.1%)	
**HRAS Mutation**			
Present	1 (1.4%)	1 (1.9%)	1
Absent	72 (98.6%)	51 (98.1%)	
**JUN Mutation**			
Present	3 (4.1%)	0 (0.0%)	0.2653
Absent	70 (95.9%)	52 (100.0%)	
**KRAS Mutation**			
Present	30 (41.1%)	18 (34.6%)	0.5839
Absent	43 (58.9%)	34 (65.4%)	
**MAP2K1 Mutation**			
Present	2 (2.7%)	3 (5.8%)	0.6484
Absent	71 (97.3%)	49 (94.2%)	
**MAP2K2 Mutation**			
Present	1 (1.4%)	1 (1.9%)	1
Absent	72 (98.6%)	51 (98.1%)	

**MAP2K4 Mutation**			
Present	3 (4.1%)	2 (3.8%)	1
Absent	70 (95.9%)	50 (96.2%)	
**MAP3K1 Mutation**			
Present	2 (2.7%)	4 (7.7%)	0.2328
Absent	71 (97.3%)	48 (92.3%)	
**MAP3K13 Mutation**			
Present	2 (2.7%)	0 (0.0%)	0.5102
Absent	71 (97.3%)	52 (100.0%)	
**MAP3K14 Mutation**			
Present	0 (0.0%)	0 (0.0%)	1
Absent	73 (100.0%)	52 (100.0%)	
**MAPK1 Mutation**			
Present	0 (0.0%)	2 (3.8%)	0.1711
Absent	73 (100.0%)	50 (96.2%)	
**MAPK3 Mutation**			
Present	1 (1.4%)	3 (5.8%)	0.3067
Absent	72 (98.6%)	49 (94.2%)	
**MAX Mutation**			
Present	0 (0.0%)	0 (0.0%)	1
Absent	73 (100.0%)	52 (100.0%)	
**MYC Mutation**			
Present	0 (0.0%)	0 (0.0%)	1
Absent	73 (100.0%)	52 (100.0%)	
**NF1 Mutation**			
Present	3 (4.1%)	10 (19.2%)	**0.01437**
Absent	70 (95.9%)	42 (80.8%)	
**NFATC2 Mutation**			
Present	0 (0.0%)	0 (0.0%)	1
Absent	73 (100.0%)	52 (100.0%)	
**NRAS Mutation**			
Present	2 (2.7%)	3 (5.8%)	0.6484
Absent	71 (97.3%)	49 (94.2%)	
**NTRK1 Mutation**			
Present	1 (1.4%)	2 (3.8%)	0.5699
Absent	72 (98.6%)	50 (96.2%)	
**NTRK2 Mutation**			
Present	1 (1.4%)	2 (3.8%)	0.5699
Absent	72 (98.6%)	50 (96.2%)	
**PAK1 Mutation**			
Present	0 (0.0%)	2 (3.8%)	0.1711
Absent	73 (100.0%)	50 (96.2%)	
**PDGFRA Mutation**			
Present	2 (2.7%)	5 (9.6%)	0.1264
Absent	71 (97.3%)	47 (90.4%)	
**PDGFRB Mutation**			
Present	4 (5.5%)	3 (5.8%)	1
Absent	69 (94.5%)	49 (94.2%)	
**PLA2G3 Mutation**			
Present	0 (0.0%)	0 (0.0%)	1
Absent	73 (100.0%)	52 (100.0%)	

**RAC1 Mutation**			
Present	0 (0.0%)	0 (0.0%)	1
Absent	73 (100.0%)	52 (100.0%)	
**RAC2 Mutation**			
Present	0 (0.0%)	0 (0.0%)	1
Absent	73 (100.0%)	52 (100.0%)	
**RAF1 Mutation**			
Present	1 (1.4%)	2 (3.8%)	0.5699
Absent	72 (98.6%)	50 (96.2%)	
**RASA1 Mutation**			
Present	1 (1.4%)	3 (5.8%)	0.3067
Absent	72 (98.6%)	49 (94.2%)	
**RPS6KA4 Mutation**			
Present	0 (0.0%)	5 (9.6%)	**0.01108**
Absent	73 (100.0%)	47 (90.4%)	
**RPS6KA6 Mutation**			
Present	0 (0.0%)	0 (0.0%)	1
Absent	73 (100.0%)	52 (100.0%)	
**RRAS Mutation**			
Present	0 (0.0%)	1 (1.9%)	0.416
Absent	73 (100.0%)	51 (98.1%)	
**RRAS2 Mutation**			
Present	0 (0.0%)	0 (0.0%)	1
Absent	73 (100.0%)	52 (100.0%)	
**SOS1 Mutation**			
Present	0 (0.0%)	1 (1.9%)	0.416
Absent	73 (100.0%)	51 (98.1%)	
**TGFB2 Mutation**			
Present	0 (0.0%)	0 (0.0%)	1
Absent	73 (100.0%)	52 (100.0%)	
**TGFBR1 Mutation**			
Present	2 (2.7%)	2 (3.8%)	1
Absent	71 (97.3%)	50 (96.2%)	
**TGFBR2 Mutation**			
Present	3 (4.1%)	6 (11.5%)	0.1617
Absent	70 (95.9%)	46 (88.5%)	
**TP53 Mutation**			
Present	57 (78.1%)	42 (80.8%)	0.8876
Absent	16 (21.9%)	10 (19.2%)	
**TRAF2 Mutation**			
Present	1 (1.4%)	0 (0.0%)	1
Absent	72 (98.6%)	52 (100.0%)	

Table S2. - Comparison of Late-Onset Hispanic/Latino (H/L) Patients Treated with FOLFOX versus Not Treated with FOLFOX.

**MAPK Pathway**			
**Gene**	**Late-Onset Hispanic/Latino Treated with FOLFOX n (%)**	**Late-Onset Hispanic/Latino Not Treated with FOLFOX n (%)**	**p-value**
**ACVR1 Mutation**			
Present	0 (0.0%)	0 (0.0%)	1

Absent	91 (100.0%)	50 (100.0%)	
**AKT1 Mutation**			
Present	2 (2.2%)	2 (4.0%)	0.615
Absent	89 (97.8%)	48 (96.0%)	
**AKT2 Mutation**			
Present	2 (2.2%)	0 (0.0%)	0.539
Absent	89 (97.8%)	50 (100.0%)	
**AKT3 Mutation**			
Present	2 (2.2%)	2 (4.0%)	0.615
Absent	89 (97.8%)	48 (96.0%)	
**BRAF Mutation**			
Present	15 (16.5%)	8 (16.0%)	1
Absent	76 (83.5%)	42 (84.0%)	
**CACNA1H Mutation**			
Present	0 (0.0%)	0 (0.0%)	1
Absent	91 (100.0%)	50 (100.0%)	
**CACNA2D1 Mutation**			
Present	0 (0.0%)	0 (0.0%)	1
Absent	91 (100.0%)	50 (100.0%)	
**CACNB1 Mutation**			
Present	0 (0.0%)	0 (0.0%)	1
Absent	91 (100.0%)	50 (100.0%)	
**CDC42 Mutation**			
Present	1 (1.1%)	1 (2.0%)	1
Absent	90 (98.9%)	49 (98.0%)	
**CRKL Mutation**			
Present	0 (0.0%)	0 (0.0%)	1
Absent	91 (100.0%)	50 (100.0%)	
**DAXX Mutation**			
Present	0 (0.0%)	1 (2.0%)	0.3546
Absent	91 (100.0%)	49 (98.0%)	
**DUSP4 Mutation**			
Present	1 (1.1%)	0 (0.0%)	1
Absent	90 (98.9%)	50 (100.0%)	
**EGFR Mutation**			
Present	1 (1.1%)	3 (6.0%)	0.1276
Absent	90 (98.9%)	47 (94.0%)	
**FGF13 Mutation**			
Present	1 (1.1%)	0 (0.0%)	1
Absent	90 (98.9%)	50 (100.0%)	
**FGF19 Mutation**			
Present	1 (1.1%)	2 (4.0%)	0.2866
Absent	90 (98.9%)	48 (96.0%)	
**FGF3 Mutation**			
Present	2 (2.2%)	0 (0.0%)	0.539
Absent	89 (97.8%)	50 (100.0%)	
**FGF4 Mutation**			
Present	0 (0.0%)	0 (0.0%)	1
Absent	91 (100.0%)	50 (100.0%)	
**FGFR1 Mutation**			
Present	0 (0.0%)	0 (0.0%)	1
Absent	91 (100.0%)	50 (100.0%)	

**FGFR2 Mutation**			
Present	0 (0.0%)	1 (2.0%)	0.3546
Absent	91 (100.0%)	49 (98.0%)	
**FGFR3 Mutation**			
Present	3 (3.3%)	2 (4.0%)	1
Absent	88 (96.7%)	48 (96.0%)	
**FGFR4 Mutation**			
Present	1 (1.1%)	1 (2.0%)	1
Absent	90 (98.9%)	49 (98.0%)	
**HRAS Mutation**			
Present	0 (0.0%)	1 (2.0%)	0.3546
Absent	91 (100.0%)	49 (98.0%)	
**JUN Mutation**			
Present	0 (0.0%)	0 (0.0%)	1
Absent	91 (100.0%)	50 (100.0%)	
**KRAS Mutation**			
Present	39 (42.9%)	20 (40.0%)	0.8803
Absent	52 (57.1%)	30 (60.0%)	
**MAP2K1 Mutation**			
Present	0 (0.0%)	0 (0.0%)	1
Absent	91 (100.0%)	50 (100.0%)	
**MAP2K2 Mutation**			
Present	2 (2.2%)	1 (2.0%)	1
Absent	89 (97.8%)	49 (98.0%)	
**MAP2K4 Mutation**			
Present	1 (1.1%)	1 (2.0%)	1
Absent	90 (98.9%)	49 (98.0%)	
**MAP3K1 Mutation**			
Present	1 (1.1%)	1 (2.0%)	1
Absent	90 (98.9%)	49 (98.0%)	
**MAP3K13 Mutation**			
Present	2 (2.2%)	0 (0.0%)	0.539
Absent	89 (97.8%)	50 (100.0%)	
**MAP3K14 Mutation**			
Present	0 (0.0%)	0 (0.0%)	1
Absent	91 (100.0%)	50 (100.0%)	
**MAPK1 Mutation**			
Present	0 (0.0%)	0 (0.0%)	1
Absent	91 (100.0%)	50 (100.0%)	
**MAPK3 Mutation**			
Present	1 (1.1%)	0 (0.0%)	1
Absent	90 (98.9%)	50 (100.0%)	
**MAX Mutation**			
Present	0 (0.0%)	0 (0.0%)	1
Absent	91 (100.0%)	50 (100.0%)	
**MYC Mutation**			
Present	0 (0.0%)	2 (4.0%)	0.1241
Absent	91 (100.0%)	48 (96.0%)	
**NF1 Mutation**			
Present	2 (2.2%)	2 (4.0%)	0.615
Absent	89 (97.8%)	48 (96.0%)	

**NFATC2 Mutation**			
Present	0 (0.0%)	0 (0.0%)	1
Absent	91 (100.0%)	50 (100.0%)	
**NRAS Mutation**			
Present	6 (6.6%)	3 (6.0%)	1
Absent	85 (93.4%)	47 (94.0%)	
**NTRK1 Mutation**			
Present	2 (2.2%)	2 (4.0%)	0.615
Absent	89 (97.8%)	48 (96.0%)	
**NTRK2 Mutation**			
Present	0 (0.0%)	3 (6.0%)	**0.04286**
Absent	91 (100.0%)	47 (94.0%)	
**PAK1 Mutation**			
Present	0 (0.0%)	0 (0.0%)	1
Absent	91 (100.0%)	50 (100.0%)	
**PDGFRA Mutation**			
Present	0 (0.0%)	0 (0.0%)	1
Absent	91 (100.0%)	50 (100.0%)	
**PDGFRB Mutation**			
Present	0 (0.0%)	3 (6.0%)	**0.04286**
Absent	91 (100.0%)	47 (94.0%)	
**PLA2G3 Mutation**			
Present	0 (0.0%)	0 (0.0%)	1
Absent	91 (100.0%)	50 (100.0%)	
**RAC1 Mutation**			
Present	0 (0.0%)	0 (0.0%)	1
Absent	91 (100.0%)	50 (100.0%)	
**RAC2 Mutation**			
Present	0 (0.0%)	1 (2.0%)	0.3546
Absent	91 (100.0%)	49 (98.0%)	
**RAF1 Mutation**			
Present	2 (2.2%)	1 (2.0%)	1
Absent	89 (97.8%)	49 (98.0%)	
**RASA1 Mutation**			
Present	4 (4.4%)	1 (2.0%)	0.6557
Absent	87 (95.6%)	49 (98.0%)	
**RPS6KA4 Mutation**			
Present	3 (3.3%)	3 (6.0%)	0.6659
Absent	88 (96.7%)	47 (94.0%)	
**RPS6KA6 Mutation**			
Present	0 (0.0%)	0 (0.0%)	1
Absent	91 (100.0%)	50 (100.0%)	
**RRAS Mutation**			
Present	0 (0.0%)	0 (0.0%)	1
Absent	91 (100.0%)	50 (100.0%)	
**RRAS2 Mutation**			
Present	1 (1.1%)	2 (4.0%)	0.2866
Absent	90 (98.9%)	48 (96.0%)	
**SOS1 Mutation**			
Present	3 (3.3%)	0 (0.0%)	0.5523
Absent	88 (96.7%)	50 (100.0%)	

**TGFB2 Mutation**			
Present	0 (0.0%)	0 (0.0%)	1
Absent	91 (100.0%)	50 (100.0%)	
**TGFBR1 Mutation**			
Present	3 (3.3%)	1 (2.0%)	1
Absent	88 (96.7%)	49 (98.0%)	
**TGFBR2 Mutation**			
Present	5 (5.5%)	3 (6.0%)	1
Absent	86 (94.5%)	47 (94.0%)	
**TP53 Mutation**			
Present	71 (78.0%)	36 (72.0%)	0.5526
Absent	20 (22.0%)	14 (28.0%)	
**TRAF2 Mutation**			
Present	0 (0.0%)	1 (2.0%)	0.3546
Absent	91 (100.0%)	49 (98.0%)	

Table S3. - Comparison of Early-Onset Non-Hispanic White (NHW) Patients Treated with FOLFOX versus Not Treated with FOLFOX**.**

**MAPK Pathway**			
**Gene**	**Early-Onset NHW Treated with FOLFOX n (%)**	**Early-Onset NHW Not Treated with FOLFOX n (%)**	**p-value**
**ACVR1 Mutation**			
Present	4 (1.1%)	5 (1.7%)	0.522
Absent	371 (98.9%)	297 (98.3%)	
**AKT1 Mutation**			
Present	9 (2.4%)	4 (1.3%)	0.4034
Absent	366 (97.6%)	298 (98.7%)	
**AKT2 Mutation**			
Present	2 (0.5%)	2 (0.7%)	1
Absent	373 (99.5%)	300 (99.3%)	
**AKT3 Mutation**			
Present	3 (0.8%)	9 (3.0%)	**0.04069**
Absent	372 (99.2%)	293 (97.0%)	
**BRAF Mutation**			
Present	27 (7.2%)	24 (7.9%)	0.8262
Absent	348 (92.8%)	278 (92.1%)	
**CACNA1H Mutation**			
Present	0 (0.0%)	0 (0.0%)	1
Absent	375 (100.0%)	302 (100.0%)	
**CACNA2D1 Mutation**			
Present	0 (0.0%)	0 (0.0%)	1
Absent	375 (100.0%)	302 (100.0%)	
**CACNB1 Mutation**			
Present	0 (0.0%)	0 (0.0%)	1
Absent	375 (100.0%)	302 (100.0%)	
**CDC42 Mutation**			
Present	0 (0.0%)	2 (0.7%)	0.1986
Absent	375 (100.0%)	300 (99.3%)	
**CRKL Mutation**			
Present	1 (0.3%)	3 (1.0%)	0.3291

Absent	374 (99.7%)	299 (99.0%)	
**DAXX Mutation**			
Present	3 (0.8%)	6 (2.0%)	0.1977
Absent	372 (99.2%)	296 (98.0%)	
**DUSP4 Mutation**			
Present	1 (0.3%)	1 (0.3%)	1
Absent	374 (99.7%)	301 (99.7%)	
**EGFR Mutation**			
Present	5 (1.3%)	5 (1.7%)	0.98
Absent	370 (98.7%)	297 (98.3%)	
**FGF13 Mutation**			
Present	0 (0.0%)	0 (0.0%)	1
Absent	375 (100.0%)	302 (100.0%)	
**FGF19 Mutation**			
Present	3 (0.8%)	4 (1.3%)	0.706
Absent	372 (99.2%)	298 (98.7%)	
**FGF3 Mutation**			
Present	4 (1.1%)	5 (1.7%)	0.522
Absent	371 (98.9%)	297 (98.3%)	
**FGF4 Mutation**			
Present	0 (0.0%)	5 (1.7%)	**0.01734**
Absent	375 (100.0%)	297 (98.3%)	
**FGFR1 Mutation**			
Present	10 (2.7%)	6 (2.0%)	0.7456
Absent	365 (97.3%)	296 (98.0%)	
**FGFR2 Mutation**			
Present	5 (1.3%)	9 (3.0%)	0.2206
Absent	370 (98.7%)	293 (97.0%)	
**FGFR3 Mutation**			
Present	5 (1.3%)	11 (3.6%)	0.08699
Absent	370 (98.7%)	291 (96.4%)	
**FGFR4 Mutation**			
Present	6 (1.6%)	5 (1.7%)	1
Absent	369 (98.4%)	297 (98.3%)	
**HRAS Mutation**			
Present	3 (0.8%)	2 (0.7%)	1
Absent	372 (99.2%)	300 (99.3%)	
**JUN Mutation**			
Present	12 (3.2%)	2 (0.7%)	**0.02726**
Absent	363 (96.8%)	300 (99.3%)	
**KRAS Mutation**			
Present	163 (43.5%)	124 (41.1%)	0.5811
Absent	212 (56.5%)	178 (58.9%)	
**MAP2K1 Mutation**			
Present	6 (1.6%)	4 (1.3%)	1
Absent	369 (98.4%)	298 (98.7%)	
**MAP2K2 Mutation**			
Present	3 (0.8%)	1 (0.3%)	0.6326
Absent	372 (99.2%)	301 (99.7%)	
**MAP2K4 Mutation**			
Present	6 (1.6%)	9 (3.0%)	0.3421
Absent	369 (98.4%)	293 (97.0%)	

**MAP3K1 Mutation**			
Present	10 (2.7%)	12 (4.0%)	0.4622
Absent	365 (97.3%)	290 (96.0%)	
**MAP3K13 Mutation**			
Present	5 (1.3%)	9 (3.0%)	0.2206
Absent	370 (98.7%)	293 (97.0%)	
**MAP3K14 Mutation**			
Present	1 (0.3%)	0 (0.0%)	1
Absent	374 (99.7%)	302 (100.0%)	
**MAPK1 Mutation**			
Present	3 (0.8%)	3 (1.0%)	1
Absent	372 (99.2%)	299 (99.0%)	
**MAPK3 Mutation**			
Present	1 (0.3%)	3 (1.0%)	0.3291
Absent	374 (99.7%)	299 (99.0%)	
**MAX Mutation**			
Present	4 (1.1%)	1 (0.3%)	0.3877
Absent	371 (98.9%)	301 (99.7%)	
**MYC Mutation**			
Present	2 (0.5%)	1 (0.3%)	1
Absent	373 (99.5%)	301 (99.7%)	
**NF1 Mutation**			
Present	17 (4.5%)	18 (6.0%)	0.5099
Absent	358 (95.5%)	284 (94.0%)	
**NFATC2 Mutation**			
Present	0 (0.0%)	0 (0.0%)	1
Absent	375 (100.0%)	302 (100.0%)	
**NRAS Mutation**			
Present	12 (3.2%)	6 (2.0%)	0.358
Absent	363 (96.8%)	296 (98.0%)	
**NTRK1 Mutation**			
Present	10 (2.7%)	9 (3.0%)	0.9909
Absent	365 (97.3%)	293 (97.0%)	
**NTRK2 Mutation**			
Present	5 (1.3%)	8 (2.6%)	0.3379
Absent	370 (98.7%)	294 (97.4%)	
**PAK1 Mutation**			
Present	7 (1.9%)	3 (1.0%)	0.5245
Absent	368 (98.1%)	299 (99.0%)	
**PDGFRA Mutation**			
Present	14 (3.7%)	16 (5.3%)	0.4263
Absent	361 (96.3%)	286 (94.7%)	
**PDGFRB Mutation**			
Present	3 (0.8%)	7 (2.3%)	0.1197
Absent	372 (99.2%)	295 (97.7%)	
**PLA2G3 Mutation**			
Present	0 (0.0%)	0 (0.0%)	1
Absent	375 (100.0%)	302 (100.0%)	
**RAC1 Mutation**			
Present	1 (0.3%)	3 (1.0%)	0.3291
Absent	374 (99.7%)	299 (99.0%)	

**RAC2 Mutation**			
Present	2 (0.5%)	2 (0.7%)	1
Absent	373 (99.5%)	300 (99.3%)	
**RAF1 Mutation**			
Present	9 (2.4%)	9 (3.0%)	0.8211
Absent	366 (97.6%)	293 (97.0%)	
**RASA1 Mutation**			
Present	9 (2.4%)	14 (4.6%)	0.1667
Absent	366 (97.6%)	288 (95.4%)	
**RPS6KA4 Mutation**			
Present	5 (1.3%)	8 (2.6%)	0.3379
Absent	370 (98.7%)	294 (97.4%)	
**RPS6KA6 Mutation**			
Present	0 (0.0%)	1 (0.3%)	0.4461
Absent	375 (100.0%)	301 (99.7%)	
**RRAS Mutation**			
Present	4 (1.1%)	0 (0.0%)	0.1326
Absent	371 (98.9%)	302 (100.0%)	
**RRAS2 Mutation**			
Present	0 (0.0%)	4 (1.3%)	**0.03916**
Absent	375 (100.0%)	298 (98.7%)	
**SOS1 Mutation**			
Present	7 (1.9%)	11 (3.6%)	0.2351
Absent	368 (98.1%)	291 (96.4%)	
**TGFB2 Mutation**			
Present	0 (0.0%)	0 (0.0%)	1
Absent	375 (100.0%)	302 (100.0%)	
**TGFBR1 Mutation**			
Present	8 (2.1%)	9 (3.0%)	0.6506
Absent	367 (97.9%)	293 (97.0%)	
**TGFBR2 Mutation**			
Present	11 (2.9%)	16 (5.3%)	0.1721
Absent	364 (97.1%)	286 (94.7%)	
**TP53 Mutation**			
Present	296 (78.9%)	228 (75.5%)	0.3319
Absent	79 (21.1%)	74 (24.5%)	
**TRAF2 Mutation**			
Present	5 (1.3%)	4 (1.3%)	1
Absent	370 (98.7%)	298 (98.7%)	

Table S4. - Comparison of Late-Onset Non-Hispanic White (NHW) Patients Treated with FOLFOX versus Not Treated with FOLFOX.

**MAPK Pathway**			
**Gene**	**Late-Onset NHW Treated with FOLFOX n (%)**	**Late-Onset NHW Not Treated with FOLFOX n (%)**	**p-value**
**ACVR1 Mutation**			
Present	7 (0.8%)	8 (1.2%)	0.504
Absent	912 (99.2%)	645 (98.8%)	
**AKT1 Mutation**			
Present	16 (1.7%)	19 (2.9%)	0.1694

Absent	903 (98.3%)	634 (97.1%)	
**AKT2 Mutation**			
Present	6 (0.7%)	12 (1.8%)	0.05296
Absent	913 (99.3%)	641 (98.2%)	
**AKT3 Mutation**			
Present	9 (1.0%)	12 (1.8%)	0.2158
Absent	910 (99.0%)	641 (98.2%)	
**BRAF Mutation**			
Present	102 (11.1%)	93 (14.2%)	0.07422
Absent	817 (88.9%)	560 (85.8%)	
**CACNA1H Mutation**			
Present	0 (0.0%)	1 (0.2%)	0.4154
Absent	919 (100.0%)	652 (99.8%)	
**CACNA2D1 Mutation**			
Present	1 (0.1%)	0 (0.0%)	1
Absent	918 (99.9%)	653 (100.0%)	
**CACNB1 Mutation**			
Present	1 (0.1%)	0 (0.0%)	1
Absent	918 (99.9%)	653 (100.0%)	
**CDC42 Mutation**			
Present	2 (0.2%)	2 (0.3%)	1
Absent	917 (99.8%)	651 (99.7%)	
**CRKL Mutation**			
Present	1 (0.1%)	8 (1.2%)	**0.004928**
Absent	918 (99.9%)	645 (98.8%)	
**DAXX Mutation**			
Present	14 (1.5%)	13 (2.0%)	0.5559
Absent	905 (98.5%)	640 (98.0%)	
**DUSP4 Mutation**			
Present	1 (0.1%)	6 (0.9%)	**0.02292**
Absent	918 (99.9%)	647 (99.1%)	
**EGFR Mutation**			
Present	22 (2.4%)	24 (3.7%)	0.1823
Absent	897 (97.6%)	629 (96.3%)	
**FGF13 Mutation**			
Present	0 (0.0%)	0 (0.0%)	1
Absent	919 (100.0%)	653 (100.0%)	
**FGF19 Mutation**			
Present	5 (0.5%)	5 (0.8%)	0.8237
Absent	914 (99.5%)	648 (99.2%)	
**FGF3 Mutation**			
Present	13 (1.4%)	14 (2.1%)	0.3682
Absent	906 (98.6%)	639 (97.9%)	
**FGF4 Mutation**			
Present	1 (0.1%)	4 (0.6%)	0.167
Absent	918 (99.9%)	649 (99.4%)	
**FGFR1 Mutation**			
Present	15 (1.6%)	16 (2.5%)	0.3343
Absent	904 (98.4%)	637 (97.5%)	
**FGFR2 Mutation**			
Present	8 (0.9%)	11 (1.7%)	0.222
Absent	911 (99.1%)	642 (98.3%)	

**FGFR3 Mutation**			
Present	16 (1.7%)	19 (2.9%)	0.1694
Absent	903 (98.3%)	634 (97.1%)	
**FGFR4 Mutation**			
Present	17 (1.8%)	22 (3.4%)	0.08119
Absent	902 (98.2%)	631 (96.6%)	
**HRAS Mutation**			
Present	4 (0.4%)	6 (0.9%)	0.3353
Absent	915 (99.6%)	647 (99.1%)	
**JUN Mutation**			
Present	13 (1.4%)	21 (3.2%)	**0.02486**
Absent	906 (98.6%)	632 (96.8%)	
**KRAS Mutation**			
Present	403 (43.9%)	279 (42.7%)	0.6948
Absent	516 (56.1%)	374 (57.3%)	
**MAP2K1 Mutation**			
Present	12 (1.3%)	10 (1.5%)	0.8749
Absent	907 (98.7%)	643 (98.5%)	
**MAP2K2 Mutation**			
Present	7 (0.8%)	10 (1.5%)	0.2276
Absent	912 (99.2%)	643 (98.5%)	
**MAP2K4 Mutation**			
Present	18 (2.0%)	17 (2.6%)	0.4963
Absent	901 (98.0%)	636 (97.4%)	
**MAP3K1 Mutation**			
Present	27 (2.9%)	31 (4.7%)	0.08193
Absent	892 (97.1%)	622 (95.3%)	
**MAP3K13 Mutation**			
Present	16 (1.7%)	15 (2.3%)	0.5503
Absent	903 (98.3%)	638 (97.7%)	
**MAP3K14 Mutation**			
Present	0 (0.0%)	0 (0.0%)	1
Absent	919 (100.0%)	653 (100.0%)	
**MAPK1 Mutation**			
Present	1 (0.1%)	6 (0.9%)	**0.02292**
Absent	918 (99.9%)	647 (99.1%)	
**MAPK3 Mutation**			
Present	4 (0.4%)	5 (0.8%)	0.5023
Absent	915 (99.6%)	648 (99.2%)	
**MAX Mutation**			
Present	7 (0.8%)	5 (0.8%)	1
Absent	912 (99.2%)	648 (99.2%)	
**MYC Mutation**			
Present	7 (0.8%)	9 (1.4%)	0.3445
Absent	912 (99.2%)	644 (98.6%)	
**NF1 Mutation**			
Present	46 (5.0%)	44 (6.7%)	0.178
Absent	873 (95.0%)	609 (93.3%)	
**NFATC2 Mutation**			
Present	1 (0.1%)	0 (0.0%)	1
Absent	918 (99.9%)	653 (100.0%)	

**NRAS Mutation**			
Present	36 (3.9%)	19 (2.9%)	0.3512
Absent	883 (96.1%)	634 (97.1%)	
**NTRK1 Mutation**			
Present	14 (1.5%)	14 (2.1%)	0.4696
Absent	905 (98.5%)	639 (97.9%)	
**NTRK2 Mutation**			
Present	16 (1.7%)	9 (1.4%)	0.7173
Absent	903 (98.3%)	644 (98.6%)	
**PAK1 Mutation**			
Present	8 (0.9%)	6 (0.9%)	1
Absent	911 (99.1%)	647 (99.1%)	
**PDGFRA Mutation**			
Present	18 (2.0%)	24 (3.7%)	0.05469
Absent	901 (98.0%)	629 (96.3%)	
**PDGFRB Mutation**			
Present	16 (1.7%)	16 (2.5%)	0.4237
Absent	903 (98.3%)	637 (97.5%)	
**PLA2G3 Mutation**			
Present	0 (0.0%)	1 (0.2%)	0.4154
Absent	919 (100.0%)	652 (99.8%)	
**RAC1 Mutation**			
Present	0 (0.0%)	3 (0.5%)	0.07148
Absent	919 (100.0%)	650 (99.5%)	
**RAC2 Mutation**			
Present	0 (0.0%)	3 (0.5%)	0.07148
Absent	919 (100.0%)	650 (99.5%)	
**RAF1 Mutation**			
Present	8 (0.9%)	19 (2.9%)	0.004111
Absent	911 (99.1%)	634 (97.1%)	
**RASA1 Mutation**			
Present	19 (2.1%)	24 (3.7%)	0.07688
Absent	900 (97.9%)	629 (96.3%)	
**RPS6KA4 Mutation**			
Present	17 (1.8%)	34 (5.2%)	0.0003744
Absent	902 (98.2%)	619 (94.8%)	
**RPS6KA6 Mutation**			
Present	0 (0.0%)	0 (0.0%)	1
Absent	919 (100.0%)	653 (100.0%)	
**RRAS Mutation**			
Present	2 (0.2%)	8 (1.2%)	**0.02024**
Absent	917 (99.8%)	645 (98.8%)	
**RRAS2 Mutation**			
Present	5 (0.5%)	3 (0.5%)	1
Absent	914 (99.5%)	650 (99.5%)	
**SOS1 Mutation**			
Present	8 (0.9%)	15 (2.3%)	**0.03501**
Absent	911 (99.1%)	638 (97.7%)	
**TGFB2 Mutation**			
Present	1 (0.1%)	0 (0.0%)	1
Absent	918 (99.9%)	653 (100.0%)	

**TGFBR1 Mutation**			
Present	13 (1.4%)	12 (1.8%)	0.6482
Absent	906 (98.6%)	641 (98.2%)	
**TGFBR2 Mutation**			
Present	38 (4.1%)	46 (7.0%)	0.01578
Absent	881 (95.9%)	607 (93.0%)	
**TP53 Mutation**			
Present	675 (73.4%)	445 (68.1%)	0.02559
Absent	244 (26.6%)	208 (31.9%)	
**TRAF2 Mutation**			
Present	10 (1.1%)	15 (2.3%)	0.09226
Absent	909 (98.9%)	638 (97.7%)	

Table S5. - Comparison of Early-Onset versus Late-Onset Hispanic/Latino (H/L) Patients Treated with FOLFOX.

**MAPK Pathway**			
**Gene**	**Early-Onset Hispanic/Latino Treated with FOLFOX n (%)**	**Late-Onset Hispanic/Latino Treated with FOLFOX n (%)**	**p-value**
**ACVR1 Mutation**			
Present	1 (1.4%)	0 (0.0%)	0.4451
Absent	72 (98.6%)	91 (100.0%)	
**AKT1 Mutation**			
Present	4 (5.5%)	2 (2.2%)	0.4082
Absent	69 (94.5%)	89 (97.8%)	
**AKT2 Mutation**			
Present	0 (0.0%)	2 (2.2%)	0.503
Absent	73 (100.0%)	89 (97.8%)	
**AKT3 Mutation**			
Present	2 (2.7%)	2 (2.2%)	1
Absent	71 (97.3%)	89 (97.8%)	
**BRAF Mutation**			
Present	3 (4.1%)	15 (16.5%)	**0.01229**
Absent	70 (95.9%)	76 (83.5%)	
**CACNA1H Mutation**			
Present	0 (0.0%)	0 (0.0%)	1
Absent	73 (100.0%)	91 (100.0%)	
**CACNA2D1 Mutation**			
Present	0 (0.0%)	0 (0.0%)	1
Absent	73 (100.0%)	91 (100.0%)	
**CACNB1 Mutation**			
Present	0 (0.0%)	0 (0.0%)	1
Absent	73 (100.0%)	91 (100.0%)	
**CDC42 Mutation**			
Present	0 (0.0%)	1 (1.1%)	1
Absent	73 (100.0%)	90 (98.9%)	
**CRKL Mutation**			
Present	0 (0.0%)	0 (0.0%)	1
Absent	73 (100.0%)	91 (100.0%)	
**DAXX Mutation**			
Present	1 (1.4%)	0 (0.0%)	0.4451

Absent	72 (98.6%)	91 (100.0%)	
**DUSP4 Mutation**			
Present	0 (0.0%)	1 (1.1%)	1
Absent	73 (100.0%)	90 (98.9%)	
**EGFR Mutation**			
Present	1 (1.4%)	1 (1.1%)	1
Absent	72 (98.6%)	90 (98.9%)	
**FGF13 Mutation**			
Present	0 (0.0%)	1 (1.1%)	1
Absent	73 (100.0%)	90 (98.9%)	
**FGF19 Mutation**			
Present	1 (1.4%)	1 (1.1%)	1
Absent	72 (98.6%)	90 (98.9%)	
**FGF3 Mutation**			
Present	0 (0.0%)	2 (2.2%)	0.503
Absent	73 (100.0%)	89 (97.8%)	
**FGF4 Mutation**			
Present	0 (0.0%)	0 (0.0%)	1
Absent	73 (100.0%)	91 (100.0%)	
**FGFR1 Mutation**			
Present	0 (0.0%)	0 (0.0%)	1
Absent	73 (100.0%)	91 (100.0%)	
**FGFR2 Mutation**			
Present	0 (0.0%)	0 (0.0%)	1
Absent	73 (100.0%)	91 (100.0%)	
**FGFR3 Mutation**			
Present	1 (1.4%)	3 (3.3%)	0.6296
Absent	72 (98.6%)	88 (96.7%)	
**FGFR4 Mutation**			
Present	1 (1.4%)	1 (1.1%)	1
Absent	72 (98.6%)	90 (98.9%)	
**HRAS Mutation**			
Present	1 (1.4%)	0 (0.0%)	0.4451
Absent	72 (98.6%)	91 (100.0%)	
**JUN Mutation**			
Present	3 (4.1%)	0 (0.0%)	0.08617
Absent	70 (95.9%)	91 (100.0%)	
**KRAS Mutation**			
Present	30 (41.1%)	39 (42.9%)	0.8742
Absent	43 (58.9%)	52 (57.1%)	
**MAP2K1 Mutation**			
Present	2 (2.7%)	0 (0.0%)	0.1966
Absent	71 (97.3%)	91 (100.0%)	
**MAP2K2 Mutation**			
Present	1 (1.4%)	2 (2.2%)	1
Absent	72 (98.6%)	89 (97.8%)	
**MAP2K4 Mutation**			
Present	3 (4.1%)	1 (1.1%)	0.3243
Absent	70 (95.9%)	90 (98.9%)	
**MAP3K1 Mutation**			
Present	2 (2.7%)	1 (1.1%)	0.5858
Absent	71 (97.3%)	90 (98.9%)	

**MAP3K13 Mutation**			
Present	2 (2.7%)	2 (2.2%)	1
Absent	71 (97.3%)	89 (97.8%)	
**MAP3K14 Mutation**			
Present	0 (0.0%)	0 (0.0%)	1
Absent	73 (100.0%)	91 (100.0%)	
**MAPK1 Mutation**			
Present	0 (0.0%)	0 (0.0%)	1
Absent	73 (100.0%)	91 (100.0%)	
**MAPK3 Mutation**			
Present	1 (1.4%)	1 (1.1%)	1
Absent	72 (98.6%)	90 (98.9%)	
**MAX Mutation**			
Present	0 (0.0%)	0 (0.0%)	1
Absent	73 (100.0%)	91 (100.0%)	
**MYC Mutation**			
Present	0 (0.0%)	0 (0.0%)	1
Absent	73 (100.0%)	91 (100.0%)	
**NF1 Mutation**			
Present	3 (4.1%)	2 (2.2%)	0.6566
Absent	70 (95.9%)	89 (97.8%)	
**NFATC2 Mutation**			
Present	0 (0.0%)	0 (0.0%)	1
Absent	73 (100.0%)	91 (100.0%)	
**NRAS Mutation**			
Present	2 (2.7%)	6 (6.6%)	0.3012
Absent	71 (97.3%)	85 (93.4%)	
**NTRK1 Mutation**			
Present	1 (1.4%)	2 (2.2%)	1
Absent	72 (98.6%)	89 (97.8%)	
**NTRK2 Mutation**			
Present	1 (1.4%)	0 (0.0%)	0.4451
Absent	72 (98.6%)	91 (100.0%)	
**PAK1 Mutation**			
Present	0 (0.0%)	0 (0.0%)	1
Absent	73 (100.0%)	91 (100.0%)	
**PDGFRA Mutation**			
Present	2 (2.7%)	0 (0.0%)	0.1966
Absent	71 (97.3%)	91 (100.0%)	
**PDGFRB Mutation**			
Present	4 (5.5%)	0 (0.0%)	**0.03747**
Absent	69 (94.5%)	91 (100.0%)	
**PLA2G3 Mutation**			
Present	0 (0.0%)	0 (0.0%)	1
Absent	73 (100.0%)	91 (100.0%)	
**RAC1 Mutation**			
Present	0 (0.0%)	0 (0.0%)	1
Absent	73 (100.0%)	91 (100.0%)	
**RAC2 Mutation**			
Present	0 (0.0%)	0 (0.0%)	1
Absent	73 (100.0%)	91 (100.0%)	

**RAF1 Mutation**			
Present	1 (1.4%)	2 (2.2%)	1
Absent	72 (98.6%)	89 (97.8%)	
**RASA1 Mutation**			
Present	1 (1.4%)	4 (4.4%)	0.3826
Absent	72 (98.6%)	87 (95.6%)	
**RPS6KA4 Mutation**			
Present	0 (0.0%)	3 (3.3%)	0.2545
Absent	73 (100.0%)	88 (96.7%)	
**RPS6KA6 Mutation**			
Present	0 (0.0%)	0 (0.0%)	1
Absent	73 (100.0%)	91 (100.0%)	
**RRAS Mutation**			
Present	0 (0.0%)	0 (0.0%)	1
Absent	73 (100.0%)	91 (100.0%)	
**RRAS2 Mutation**			
Present	0 (0.0%)	1 (1.1%)	1
Absent	73 (100.0%)	90 (98.9%)	
**SOS1 Mutation**			
Present	0 (0.0%)	3 (3.3%)	0.2545
Absent	73 (100.0%)	88 (96.7%)	
**TGFB2 Mutation**			
Present	0 (0.0%)	0 (0.0%)	1
Absent	73 (100.0%)	91 (100.0%)	
**TGFBR1 Mutation**			
Present	2 (2.7%)	3 (3.3%)	1
Absent	71 (97.3%)	88 (96.7%)	
**TGFBR2 Mutation**			
Present	3 (4.1%)	5 (5.5%)	0.7334
Absent	70 (95.9%)	86 (94.5%)	
**TP53 Mutation**			
Present	57 (78.1%)	71 (78.0%)	1
Absent	16 (21.9%)	20 (22.0%)	
**TRAF2 Mutation**			
Present	1 (1.4%)	0 (0.0%)	1
Absent	72 (98.6%)	91 (100.0%)	

Table S6. - Comparison of Early-Onset versus Late-Onset Hispanic/Latino (H/L) Patients Not Treated with FOLFOX.

**MAPK Pathway**			
**Gene**	**Early-Onset Hispanic/Latino Not Treated with FOLFOX n (%)**	**Late-Onset Hispanic/Latino Not Treated with FOLFOX n (%)**	**p-value**
**ACVR1 Mutation**			
Present	2 (3.8%)	0 (0.0%)	0.4952
Absent	50 (96.2%)	50 (100.0%)	
**AKT1 Mutation**			
Present	2 (3.8%)	2 (4.0%)	1
Absent	50 (96.2%)	48 (96.0%)	
**AKT2 Mutation**			
Present	0 (0.0%)	0 (0.0%)	1

Absent	52 (100.0%)	50 (100.0%)	
**AKT3 Mutation**			
Present	3 (5.8%)	2 (4.0%)	1
Absent	49 (94.2%)	48 (96.0%)	
**BRAF Mutation**			
Present	4 (7.7%)	8 (16.0%)	0.2303
Absent	48 (92.3%)	42 (84.0%)	
**CACNA1H Mutation**			
Present	0 (0.0%)	0 (0.0%)	1
Absent	52 (100.0%)	50 (100.0%)	
**CACNA2D1 Mutation**			
Present	0 (0.0%)	0 (0.0%)	1
Absent	52 (100.0%)	50 (100.0%)	
**CACNB1 Mutation**			
Present	0 (0.0%)	0 (0.0%)	1
Absent	52 (100.0%)	50 (100.0%)	
**CDC42 Mutation**			
Present	0 (0.0%)	1 (2.0%)	0.4902
Absent	52 (100.0%)	49 (98.0%)	
**CRKL Mutation**			
Present	0 (0.0%)	0 (0.0%)	1
Absent	52 (100.0%)	50 (100.0%)	
**DAXX Mutation**			
Present	2 (3.8%)	1 (2.0%)	1
Absent	50 (96.2%)	49 (98.0%)	
**DUSP4 Mutation**			
Present	0 (0.0%)	0 (0.0%)	1
Absent	52 (100.0%)	50 (100.0%)	
**EGFR Mutation**			
Present	3 (5.8%)	3 (6.0%)	1
Absent	49 (94.2%)	47 (94.0%)	
**FGF13 Mutation**			
Present	0 (0.0%)	0 (0.0%)	1
Absent	52 (100.0%)	50 (100.0%)	
**FGF19 Mutation**			
Present	0 (0.0%)	2 (4.0%)	0.2378
Absent	52 (100.0%)	48 (96.0%)	
**FGF3 Mutation**			
Present	1 (1.9%)	0 (0.0%)	1
Absent	51 (98.1%)	50 (100.0%)	
**FGF4 Mutation**			
Present	0 (0.0%)	0 (0.0%)	1
Absent	52 (100.0%)	50 (100.0%)	
**FGFR1 Mutation**			
Present	2 (3.8%)	0 (0.0%)	0.4952
Absent	50 (96.2%)	50 (100.0%)	
**FGFR2 Mutation**			
Present	4 (7.7%)	1 (2.0%)	0.3629
Absent	48 (92.3%)	49 (98.0%)	
**FGFR3 Mutation**			
Present	2 (3.8%)	2 (4.0%)	1
Absent	50 (96.2%)	48 (96.0%)	

**FGFR4 Mutation**			
Present	1 (1.9%)	1 (2.0%)	1
Absent	51 (98.1%)	49 (98.0%)	
**HRAS Mutation**			
Present	1 (1.9%)	1 (2.0%)	1
Absent	51 (98.1%)	49 (98.0%)	
**JUN Mutation**			
Present	0 (0.0%)	0 (0.0%)	1
Absent	52 (100.0%)	50 (100.0%)	
**KRAS Mutation**			
Present	18 (34.6%)	20 (40.0%)	0.7208
Absent	34 (65.4%)	30 (60.0%)	
**MAP2K1 Mutation**			
Present	3 (5.8%)	0 (0.0%)	0.2429
Absent	49 (94.2%)	50 (100.0%)	
**MAP2K2 Mutation**			
Present	1 (1.9%)	1 (2.0%)	1
Absent	51 (98.1%)	49 (98.0%)	
**MAP2K4 Mutation**			
Present	2 (3.8%)	1 (2.0%)	1
Absent	50 (96.2%)	49 (98.0%)	
**MAP3K1 Mutation**			
Present	4 (7.7%)	1 (2.0%)	0.3629
Absent	48 (92.3%)	49 (98.0%)	
**MAP3K13 Mutation**			
Present	0 (0.0%)	0 (0.0%)	1
Absent	52 (100.0%)	50 (100.0%)	
**MAP3K14 Mutation**			
Present	0 (0.0%)	0 (0.0%)	1
Absent	52 (100.0%)	50 (100.0%)	
**MAPK1 Mutation**			
Present	2 (3.8%)	0 (0.0%)	0.4952
Absent	50 (96.2%)	50 (100.0%)	
**MAPK3 Mutation**			
Present	3 (5.8%)	0 (0.0%)	0.2429
Absent	49 (94.2%)	50 (100.0%)	
**MAX Mutation**			
Present	0 (0.0%)	0 (0.0%)	1
Absent	52 (100.0%)	50 (100.0%)	
**MYC Mutation**			
Present	0 (0.0%)	2 (4.0%)	0.2378
Absent	52 (100.0%)	48 (96.0%)	
**NF1 Mutation**			
Present	10 (19.2%)	2 (4.0%)	**0.02834**
Absent	42 (80.8%)	48 (96.0%)	
**NFATC2 Mutation**			
Present	0 (0.0%)	0 (0.0%)	1
Absent	52 (100.0%)	50 (100.0%)	
**NRAS Mutation**			
Present	3 (5.8%)	3 (6.0%)	1
Absent	49 (94.2%)	47 (94.0%)	

**NTRK1 Mutation**			
Present	2 (3.8%)	2 (4.0%)	1
Absent	50 (96.2%)	48 (96.0%)	
**NTRK2 Mutation**			
Present	2 (3.8%)	3 (6.0%)	0.675
Absent	50 (96.2%)	47 (94.0%)	
**PAK1 Mutation**			
Present	2 (3.8%)	0 (0.0%)	0.4952
Absent	50 (96.2%)	50 (100.0%)	
**PDGFRA Mutation**			
Present	5 (9.6%)	0 (0.0%)	0.05664
Absent	47 (90.4%)	50 (100.0%)	
**PDGFRB Mutation**			
Present	3 (5.8%)	3 (6.0%)	1
Absent	49 (94.2%)	47 (94.0%)	
**PLA2G3 Mutation**			
Present	0 (0.0%)	0 (0.0%)	1
Absent	52 (100.0%)	50 (100.0%)	
**RAC1 Mutation**			
Present	0 (0.0%)	0 (0.0%)	1
Absent	52 (100.0%)	50 (100.0%)	
**RAC2 Mutation**			
Present	0 (0.0%)	1 (2.0%)	0.4902
Absent	52 (100.0%)	49 (98.0%)	
**RAF1 Mutation**			
Present	2 (3.8%)	1 (2.0%)	1
Absent	50 (96.2%)	49 (98.0%)	
**RASA1 Mutation**			
Present	3 (5.8%)	1 (2.0%)	0.6178
Absent	49 (94.2%)	49 (98.0%)	
**RPS6KA4 Mutation**			
Present	5 (9.6%)	3 (6.0%)	0.7157
Absent	47 (90.4%)	47 (94.0%)	
**RPS6KA6 Mutation**			
Present	0 (0.0%)	0 (0.0%)	1
Absent	52 (100.0%)	50 (100.0%)	
**RRAS Mutation**			
Present	1 (1.9%)	0 (0.0%)	1
Absent	51 (98.1%)	50 (100.0%)	
**RRAS2 Mutation**			
Present	0 (0.0%)	2 (4.0%)	0.2378
Absent	52 (100.0%)	48 (96.0%)	
**SOS1 Mutation**			
Present	1 (1.9%)	0 (0.0%)	1
Absent	51 (98.1%)	50 (100.0%)	
**TGFB2 Mutation**			
Present	0 (0.0%)	0 (0.0%)	1
Absent	52 (100.0%)	50 (100.0%)	
**TGFBR1 Mutation**			
Present	2 (3.8%)	1 (2.0%)	1
Absent	50 (96.2%)	49 (98.0%)	

	**TGFBR2 Mutation**		
Present	6 (11.5%)	3 (6.0%)	0.4882
Absent	46 (88.5%)	47 (94.0%)	
	**TP53 Mutation**		
Present	42 (80.8%)	36 (72.0%)	0.4178
Absent	10 (19.2%)	14 (28.0%)	
	**TRAF2 Mutation**		
Present	0 (0.0%)	1 (2.0%)	0.4902
Absent	52 (100.0%)	49 (98.0%)	

Table S7. - Comparison of Early-Onset versus Late-Onset Non-Hispanic White (NHW) Patients Treated with FOLFOX.

**MAPK Pathway**			
**Gene**	**Early-Onset NHW Treated with FOLFOX n (%)**	**Late-Onset NHW Treated with FOLFOX n (%)**	**p-value**
	**ACVR1 Mutation**		
Present	4 (1.1%)	7 (0.8%)	0.739
Absent	371 (98.9%)	912 (99.2%)	
**AKT1 Mutation**			
Present	9 (2.4%)	16 (1.7%)	
Absent	366 (97.6%)	903 (98.3%)	
**AKT2 Mutation**			
Present	2 (0.5%)	6 (0.7%)	1
Absent	373 (99.5%)	913 (99.3%)	
	**AKT3 Mutation**		
Present	3 (0.8%)	9 (1.0%)	1
Absent	372 (99.2%)	910 (99.0%)	
	**BRAF Mutation**		
Present	27 (7.2%)	102 (11.1%)	
Absent	348 (92.8%)	817 (88.9%)	
	**CACNA1H Mutation**		
Present	0 (0.0%)	0 (0.0%)	1
Absent	375 (100.0%)	919 (100.0%)	
	**CACNA2D1 Mutation**		
Present	0 (0.0%)	1 (0.1%)	1
Absent	375 (100.0%)	918 (99.9%)	
	**CACNB1 Mutation**		
Present	0 (0.0%)	1 (0.1%)	1
Absent	375 (100.0%)	918 (99.9%)	
	**CDC42 Mutation**		
Present	0 (0.0%)	2 (0.2%)	1
Absent	375 (100.0%)	917 (99.8%)	
	**CRKL Mutation**		
Present	1 (0.3%)	1 (0.1%)	0.4958
Absent	374 (99.7%)	918 (99.9%)	
	**DAXX Mutation**		
Present	3 (0.8%)	14 (1.5%)	0.4224
Absent	372 (99.2%)	905 (98.5%)	
	**DUSP4 Mutation**		
Present	1 (0.3%)	1 (0.1%)	0.4958

Absent	374 (99.7%)	918 (99.9%)	
**EGFR Mutation**			
Present	5 (1.3%)	22 (2.4%)	0.319
Absent	370 (98.7%)	897 (97.6%)	
**FGF13 Mutation**			
Present	0 (0.0%)	0 (0.0%)	1
Absent	375 (100.0%)	919 (100.0%)	
**FGF19 Mutation**			
Present	3 (0.8%)	5 (0.5%)	0.6975
Absent	372 (99.2%)	914 (99.5%)	
**FGF3 Mutation**			
Present	4 (1.1%)	13 (1.4%)	0.7903
Absent	371 (98.9%)	906 (98.6%)	
**FGF4 Mutation**			
Present	0 (0.0%)	1 (0.1%)	1
Absent	375 (100.0%)	918 (99.9%)	
**FGFR1 Mutation**			
Present	10 (2.7%)	15 (1.6%)	0.3154
Absent	365 (97.3%)	904 (98.4%)	
**FGFR2 Mutation**			
Present	5 (1.3%)	8 (0.9%)	0.6526
Absent	370 (98.7%)	911 (99.1%)	
**FGFR3 Mutation**			
Present	5 (1.3%)	16 (1.7%)	0.7763
Absent	370 (98.7%)	903 (98.3%)	
**FGFR4 Mutation**			
Present	6 (1.6%)	17 (1.8%)	0.9389
Absent	369 (98.4%)	902 (98.2%)	
**HRAS Mutation**			
Present	3 (0.8%)	4 (0.4%)	0.4202
Absent	372 (99.2%)	915 (99.6%)	
**JUN Mutation**			
Present	12 (3.2%)	13 (1.4%)	0.0582
Absent	363 (96.8%)	906 (98.6%)	
**KRAS Mutation**			
Present	163 (43.5%)	403 (43.9%)	0.9482
Absent	212 (56.5%)	516 (56.1%)	
**MAP2K1 Mutation**			
Present	6 (1.6%)	12 (1.3%)	0.882
Absent	369 (98.4%)	907 (98.7%)	
**MAP2K2 Mutation**			
Present	3 (0.8%)	7 (0.8%)	1
Absent	372 (99.2%)	912 (99.2%)	
**MAP2K4 Mutation**			
Present	6 (1.6%)	18 (2.0%)	0.8362
Absent	369 (98.4%)	901 (98.0%)	
**MAP3K1 Mutation**			
Present	10 (2.7%)	27 (2.9%)	0.9348
Absent	365 (97.3%)	892 (97.1%)	
**MAP3K13 Mutation**			
Present	5 (1.3%)	16 (1.7%)	0.7763
Absent	370 (98.7%)	903 (98.3%)	

**MAP3K14 Mutation**			
Present	1 (0.3%)	0 (0.0%)	0.2898
Absent	374 (99.7%)	919 (100.0%)	
**MAPK1 Mutation**			
Present	3 (0.8%)	1 (0.1%)	0.07588
Absent	372 (99.2%)	918 (99.9%)	
**MAPK3 Mutation**			
Present	1 (0.3%)	4 (0.4%)	1
Absent	374 (99.7%)	915 (99.6%)	
**MAX Mutation**			
Present	4 (1.1%)	7 (0.8%)	0.739
Absent	371 (98.9%)	912 (99.2%)	
**MYC Mutation**			
Present	2 (0.5%)	7 (0.8%)	1
Absent	373 (99.5%)	912 (99.2%)	
**NF1 Mutation**			
Present	17 (4.5%)	46 (5.0%)	0.8293
Absent	358 (95.5%)	873 (95.0%)	
**NFATC2 Mutation**			
Present	0 (0.0%)	1 (0.1%)	1
Absent	375 (100.0%)	918 (99.9%)	
**NRAS Mutation**			
Present	12 (3.2%)	36 (3.9%)	0.6475
Absent	363 (96.8%)	883 (96.1%)	
**NTRK1 Mutation**			
Present	10 (2.7%)	14 (1.5%)	0.2478
Absent	365 (97.3%)	905 (98.5%)	
**NTRK2 Mutation**			
Present	5 (1.3%)	16 (1.7%)	0.7763
Absent	370 (98.7%)	903 (98.3%)	
**PAK1 Mutation**			
Present	7 (1.9%)	8 (0.9%)	0.2148
Absent	368 (98.1%)	911 (99.1%)	
**PDGFRA Mutation**			
Present	14 (3.7%)	18 (2.0%)	0.09539
Absent	361 (96.3%)	901 (98.0%)	
**PDGFRB Mutation**			
Present	3 (0.8%)	16 (1.7%)	0.3074
Absent	372 (99.2%)	903 (98.3%)	
**PLA2G3 Mutation**			
Present	0 (0.0%)	0 (0.0%)	1
Absent	375 (100.0%)	919 (100.0%)	
**RAC1 Mutation**			
Present	1 (0.3%)	0 (0.0%)	0.2898
Absent	374 (99.7%)	919 (100.0%)	
**RAC2 Mutation**			
Present	2 (0.5%)	0 (0.0%)	0.08382
Absent	373 (99.5%)	919 (100.0%)	
**RAF1 Mutation**			
Present	9 (2.4%)	8 (0.9%)	0.05447
Absent	366 (97.6%)	911 (99.1%)	

**RASA1 Mutation**			
Present	9 (2.4%)	19 (2.1%)	0.871
Absent	366 (97.6%)	900 (97.9%)	
**RPS6KA4 Mutation**			
Present	5 (1.3%)	17 (1.8%)	0.6781
Absent	370 (98.7%)	902 (98.2%)	
**RPS6KA6 Mutation**			
Present	0 (0.0%)	0 (0.0%)	1
Absent	375 (100.0%)	919 (100.0%)	
**RRAS Mutation**			
Present	4 (1.1%)	2 (0.2%)	0.06222
Absent	371 (98.9%)	917 (99.8%)	
**RRAS2 Mutation**			
Present	0 (0.0%)	5 (0.5%)	0.3295
Absent	375 (100.0%)	914 (99.5%)	
**SOS1 Mutation**			
Present	7 (1.9%)	8 (0.9%)	0.2178
Absent	368 (98.1%)	911 (99.1%)	
**TGFB2 Mutation**			
Present	0 (0.0%)	1 (0.1%)	1
Absent	375 (100.0%)	918 (99.9%)	
**TGFBR1 Mutation**			
Present	8 (2.1%)	13 (1.4%)	0.4928
Absent	367 (97.9%)	906 (98.6%)	
**TGFBR2 Mutation**			
Present	11 (2.9%)	38 (4.1%)	0.386
Absent	364 (97.1%)	881 (95.9%)	
**TP53 Mutation**			
Present	296 (78.9%)	675 (73.4%)	**0.04582**
Absent	79 (21.1%)	244 (26.6%)	
**TRAF2 Mutation**			
Present	5 (1.3%)	10 (1.1%)	0.9302
Absent	370 (98.7%)	909 (98.9%)	

Table S8. - Comparison of Early-Onset Hispanic/Latino (H/L) versus Early-Onset Non-Hispanic White (NHW) Patients Treated with FOLFOX.

**MAPK Pathway**			
**Gene**	**Early-Onset NHW Not Treated with FOLFOX n (%)**	**Late-Onset NHW Not Treated with FOLFOX n (%)**	**p-value**
**ACVR1 Mutation**			
Present	5 (1.7%)	8 (1.2%)	0.8153
Absent	297 (98.3%)	645 (98.8%)	
**AKT1 Mutation**			
Present	4 (1.3%)	19 (2.9%)	0.1748
Absent	298 (98.7%)	634 (97.1%)	
**AKT2 Mutation**			
Present	2 (0.7%)	12 (1.8%)	0.2466
Absent	300 (99.3%)	641 (98.2%)	
**AKT3 Mutation**			
Present	9 (3.0%)	12 (1.8%)	0.3777

Absent	293 (97.0%)	641 (98.2%)	
**BRAF Mutation**			
Present	24 (7.9%)	93 (14.2%)	**0.007983**
Absent	278 (92.1%)	560 (85.8%)	
**CACNA1H Mutation**			
Present	0 (0.0%)	1 (0.2%)	1
Absent	302 (100.0%)	652 (99.8%)	
**CACNA2D1 Mutation**			
Present	0 (0.0%)	0 (0.0%)	1
Absent	302 (100.0%)	653 (100.0%)	
**CACNB1 Mutation**			
Present	0 (0.0%)	0 (0.0%)	1
Absent	302 (100.0%)	653 (100.0%)	
**CDC42 Mutation**			
Present	2 (0.7%)	2 (0.3%)	0.5949
Absent	300 (99.3%)	651 (99.7%)	
**CRKL Mutation**			
Present	3 (1.0%)	8 (1.2%)	1
Absent	299 (99.0%)	645 (98.8%)	
**DAXX Mutation**			
Present	6 (2.0%)	13 (2.0%)	1
Absent	296 (98.0%)	640 (98.0%)	
**DUSP4 Mutation**			
Present	1 (0.3%)	6 (0.9%)	0.4424
Absent	301 (99.7%)	647 (99.1%)	
**EGFR Mutation**			
Present	5 (1.7%)	24 (3.7%)	0.1366
Absent	297 (98.3%)	629 (96.3%)	
**FGF13 Mutation**			
Present	0 (0.0%)	0 (0.0%)	1
Absent	302 (100.0%)	653 (100.0%)	
**FGF19 Mutation**			
Present	4 (1.3%)	5 (0.8%)	0.4751
Absent	298 (98.7%)	648 (99.2%)	
**FGF3 Mutation**			
Present	5 (1.7%)	14 (2.1%)	0.8
Absent	297 (98.3%)	639 (97.9%)	
**FGF4 Mutation**			
Present	5 (1.7%)	4 (0.6%)	0.151
Absent	297 (98.3%)	649 (99.4%)	
**FGFR1 Mutation**			
Present	6 (2.0%)	16 (2.5%)	0.8321
Absent	296 (98.0%)	637 (97.5%)	
**FGFR2 Mutation**			
Present	9 (3.0%)	11 (1.7%)	0.2904
Absent	293 (97.0%)	642 (98.3%)	
**FGFR3 Mutation**			
Present	11 (3.6%)	19 (2.9%)	0.6861
Absent	291 (96.4%)	634 (97.1%)	
**FGFR4 Mutation**			
Present	5 (1.7%)	22 (3.4%)	0.2021
Absent	297 (98.3%)	631 (96.6%)	

**HRAS Mutation**			
Present	2 (0.7%)	6 (0.9%)	1
Absent	300 (99.3%)	647 (99.1%)	
**JUN Mutation**			
Present	2 (0.7%)	21 (3.2%)	**0.02065**
Absent	300 (99.3%)	632 (96.8%)	
**KRAS Mutation**			
Present	124 (41.1%)	279 (42.7%)	0.6786
Absent	178 (58.9%)	374 (57.3%)	
**MAP2K1 Mutation**			
Present	4 (1.3%)	10 (1.5%)	1
Absent	298 (98.7%)	643 (98.5%)	
**MAP2K2 Mutation**			
Present	1 (0.3%)	10 (1.5%)	0.1885
Absent	301 (99.7%)	643 (98.5%)	
**MAP2K4 Mutation**			
Present	9 (3.0%)	17 (2.6%)	0.9054
Absent	293 (97.0%)	636 (97.4%)	
**MAP3K1 Mutation**			
Present	12 (4.0%)	31 (4.7%)	0.7125
Absent	290 (96.0%)	622 (95.3%)	
**MAP3K13 Mutation**			
Present	9 (3.0%)	15 (2.3%)	0.6856
Absent	293 (97.0%)	638 (97.7%)	
**MAP3K14 Mutation**			
Present	0 (0.0%)	0 (0.0%)	1
Absent	302 (100.0%)	653 (100.0%)	
**MAPK1 Mutation**			
Present	3 (1.0%)	6 (0.9%)	1
Absent	299 (99.0%)	647 (99.1%)	
**MAPK3 Mutation**			
Present	3 (1.0%)	5 (0.8%)	0.713
Absent	299 (99.0%)	648 (99.2%)	
**MAX Mutation**			
Present	1 (0.3%)	5 (0.8%)	0.671
Absent	301 (99.7%)	648 (99.2%)	
**MYC Mutation**			
Present	1 (0.3%)	9 (1.4%)	0.1837
Absent	301 (99.7%)	644 (98.6%)	
**NF1 Mutation**			
Present	18 (6.0%)	44 (6.7%)	0.7547
Absent	284 (94.0%)	609 (93.3%)	
**NFATC2 Mutation**			
Present	0 (0.0%)	0 (0.0%)	1
Absent	302 (100.0%)	653 (100.0%)	
**NRAS Mutation**			
Present	6 (2.0%)	19 (2.9%)	0.5401
Absent	296 (98.0%)	634 (97.1%)	
**NTRK1 Mutation**			
Present	9 (3.0%)	14 (2.1%)	0.5777
Absent	293 (97.0%)	639 (97.9%)	

**NTRK2 Mutation**			
Present	8 (2.6%)	9 (1.4%)	0.2636
Absent	294 (97.4%)	644 (98.6%)	
**PAK1 Mutation**			
Present	3 (1.0%)	6 (0.9%)	1
Absent	299 (99.0%)	647 (99.1%)	
**PDGFRA Mutation**			
Present	16 (5.3%)	24 (3.7%)	0.322
Absent	286 (94.7%)	629 (96.3%)	
**PDGFRB Mutation**			
Present	7 (2.3%)	16 (2.5%)	1
Absent	295 (97.7%)	637 (97.5%)	
**PLA2G3 Mutation**			
Present	0 (0.0%)	1 (0.2%)	1
Absent	302 (100.0%)	652 (99.8%)	
**RAC1 Mutation**			
Present	3 (1.0%)	3 (0.5%)	0.3873
Absent	299 (99.0%)	650 (99.5%)	
**RAC2 Mutation**			
Present	2 (0.7%)	3 (0.5%)	0.6539
Absent	300 (99.3%)	650 (99.5%)	
**RAF1 Mutation**			
Present	9 (3.0%)	19 (2.9%)	1
Absent	293 (97.0%)	634 (97.1%)	
**RASA1 Mutation**			
Present	14 (4.6%)	24 (3.7%)	0.5975
Absent	288 (95.4%)	629 (96.3%)	
**RPS6KA4 Mutation**			
Present	8 (2.6%)	34 (5.2%)	0.1046
Absent	294 (97.4%)	619 (94.8%)	
**RPS6KA6 Mutation**			
Present	1 (0.3%)	0 (0.0%)	0.3162
Absent	301 (99.7%)	653 (100.0%)	
**RRAS Mutation**			
Present	0 (0.0%)	8 (1.2%)	0.0617
Absent	302 (100.0%)	645 (98.8%)	
**RRAS2 Mutation**			
Present	4 (1.3%)	3 (0.5%)	0.2164
Absent	298 (98.7%)	650 (99.5%)	
**SOS1 Mutation**			
Present	11 (3.6%)	15 (2.3%)	0.33
Absent	291 (96.4%)	638 (97.7%)	
**TGFB2 Mutation**			
Present	0 (0.0%)	0 (0.0%)	1
Absent	302 (100.0%)	653 (100.0%)	
**TGFBR1 Mutation**			
Present	9 (3.0%)	12 (1.8%)	0.3777
Absent	293 (97.0%)	641 (98.2%)	
**TGFBR2 Mutation**			
Present	16 (5.3%)	46 (7.0%)	0.3803
Absent	286 (94.7%)	607 (93.0%)	

	**TP53 Mutation**		
Present	228 (75.5%)	445 (68.1%)	**0.02516**
Absent	74 (24.5%)	208 (31.9%)	
	**TRAF2 Mutation**		
Present	4 (1.3%)	15 (2.3%)	0.4556
Absent	298 (98.7%)	638 (97.7%)	

Table S9. - Comparison of Early-Onset Hispanic/Latino (H/L) versus Early-Onset Non-Hispanic White (NHW) Patients Not Treated with FOLFOX.

**MAPK Pathway**			
**Gene**	**Early-Onset Hispanic/Latino Treated with FOLFOX n (%)**	**Early-Onset NHW Treated with FOLFOX n (%)**	**p-value**
	**ACVR1 Mutation**		
Present	1 (1.4%)	4 (1.1%)	0.5909
Absent	72 (98.6%)	371 (98.9%)	
	**AKT1 Mutation**		
Present	4 (5.5%)	9 (2.4%)	0.2414
Absent	69 (94.5%)	366 (97.6%)	
	**AKT2 Mutation**		
Present	0 (0.0%)	2 (0.5%)	1
Absent	73 (100.0%)	373 (99.5%)	
	**AKT3 Mutation**		
Present	2 (2.7%)	3 (0.8%)	0.1883
Absent	71 (97.3%)	372 (99.2%)	
	**BRAF Mutation**		
Present	3 (4.1%)	27 (7.2%)	0.4472
Absent	70 (95.9%)	348 (92.8%)	
	**CACNA1H Mutation**		
Present	0 (0.0%)	0 (0.0%)	1
Absent	73 (100.0%)	375 (100.0%)	
	**CACNA2D1 Mutation**		
Present	0 (0.0%)	0 (0.0%)	1
Absent	73 (100.0%)	375 (100.0%)	
	**CACNB1 Mutation**		
Present	0 (0.0%)	0 (0.0%)	1
Absent	73 (100.0%)	375 (100.0%)	
	**CDC42 Mutation**		
Present	0 (0.0%)	0 (0.0%)	1
Absent	73 (100.0%)	375 (100.0%)	
	**CRKL Mutation**		
Present	0 (0.0%)	1 (0.3%)	1
Absent	73 (100.0%)	374 (99.7%)	
	**DAXX Mutation**		
Present	1 (1.4%)	3 (0.8%)	0.5104
Absent	72 (98.6%)	372 (99.2%)	
	**DUSP4 Mutation**		
Present	0 (0.0%)	1 (0.3%)	1
Absent	73 (100.0%)	374 (99.7%)	
	**EGFR Mutation**		
Present	1 (1.4%)	5 (1.3%)	1

Absent	72 (98.6%)	370 (98.7%)	
**FGF13 Mutation**			
Present	0 (0.0%)	0 (0.0%)	1
Absent	73 (100.0%)	375 (100.0%)	
**FGF19 Mutation**			
Present	1 (1.4%)	3 (0.8%)	0.5104
Absent	72 (98.6%)	372 (99.2%)	
**FGF3 Mutation**			
Present	0 (0.0%)	4 (1.1%)	1
Absent	73 (100.0%)	371 (98.9%)	
**FGF4 Mutation**			
Present	0 (0.0%)	0 (0.0%)	1
Absent	73 (100.0%)	375 (100.0%)	
**FGFR1 Mutation**			
Present	0 (0.0%)	10 (2.7%)	0.3783
Absent	73 (100.0%)	365 (97.3%)	
**FGFR2 Mutation**			
Present	0 (0.0%)	5 (1.3%)	1
Absent	73 (100.0%)	370 (98.7%)	
**FGFR3 Mutation**			
Present	1 (1.4%)	5 (1.3%)	1
Absent	72 (98.6%)	370 (98.7%)	
**FGFR4 Mutation**			
Present	1 (1.4%)	6 (1.6%)	1
Absent	72 (98.6%)	369 (98.4%)	
**HRAS Mutation**			
Present	1 (1.4%)	3 (0.8%)	0.5104
Absent	72 (98.6%)	372 (99.2%)	
**JUN Mutation**			
Present	3 (4.1%)	12 (3.2%)	0.7203
Absent	70 (95.9%)	363 (96.8%)	
**KRAS Mutation**			
Present	30 (41.1%)	163 (43.5%)	0.8064
Absent	43 (58.9%)	212 (56.5%)	
**MAP2K1 Mutation**			
Present	2 (2.7%)	6 (1.6%)	0.6222
Absent	71 (97.3%)	369 (98.4%)	
**MAP2K2 Mutation**			
Present	1 (1.4%)	3 (0.8%)	0.5104
Absent	72 (98.6%)	372 (99.2%)	
**MAP2K4 Mutation**			
Present	3 (4.1%)	6 (1.6%)	0.1678
Absent	70 (95.9%)	369 (98.4%)	
**MAP3K1 Mutation**			
Present	2 (2.7%)	10 (2.7%)	1
Absent	71 (97.3%)	365 (97.3%)	
**MAP3K13 Mutation**			
Present	2 (2.7%)	5 (1.3%)	0.3197
Absent	71 (97.3%)	370 (98.7%)	
**MAP3K14 Mutation**			
Present	0 (0.0%)	1 (0.3%)	1
Absent	73 (100.0%)	374 (99.7%)	

**MAPK1 Mutation**			
Present	0 (0.0%)	3 (0.8%)	1
Absent	73 (100.0%)	372 (99.2%)	
**MAPK3 Mutation**			
Present	1 (1.4%)	1 (0.3%)	0.2996
Absent	72 (98.6%)	374 (99.7%)	
**MAX Mutation**			
Present	0 (0.0%)	4 (1.1%)	1
Absent	73 (100.0%)	371 (98.9%)	
**MYC Mutation**			
Present	0 (0.0%)	2 (0.5%)	1
Absent	73 (100.0%)	373 (99.5%)	
**NF1 Mutation**			
Present	3 (4.1%)	17 (4.5%)	1
Absent	70 (95.9%)	358 (95.5%)	
**NFATC2 Mutation**			
Present	0 (0.0%)	0 (0.0%)	1
Absent	73 (100.0%)	375 (100.0%)	
**NRAS Mutation**			
Present	2 (2.7%)	12 (3.2%)	1
Absent	71 (97.3%)	363 (96.8%)	
**NTRK1 Mutation**			
Present	1 (1.4%)	10 (2.7%)	1
Absent	72 (98.6%)	365 (97.3%)	
**NTRK2 Mutation**			
Present	1 (1.4%)	5 (1.3%)	1
Absent	72 (98.6%)	370 (98.7%)	
**PAK1 Mutation**			
Present	0 (0.0%)	7 (1.9%)	0.6049
Absent	73 (100.0%)	368 (98.1%)	
**PDGFRA Mutation**			
Present	2 (2.7%)	14 (3.7%)	1
Absent	71 (97.3%)	361 (96.3%)	
**PDGFRB Mutation**			
Present	4 (5.5%)	3 (0.8%)	**0.01547**
Absent	69 (94.5%)	372 (99.2%)	
**PLA2G3 Mutation**			
Present	0 (0.0%)	0 (0.0%)	1
Absent	73 (100.0%)	375 (100.0%)	
**RAC1 Mutation**			
Present	0 (0.0%)	1 (0.3%)	1
Absent	73 (100.0%)	374 (99.7%)	
**RAC2 Mutation**			
Present	0 (0.0%)	2 (0.5%)	1
Absent	73 (100.0%)	373 (99.5%)	
**RAF1 Mutation**			
Present	1 (1.4%)	9 (2.4%)	1
Absent	72 (98.6%)	366 (97.6%)	
**RASA1 Mutation**			
Present	1 (1.4%)	9 (2.4%)	1
Absent	72 (98.6%)	366 (97.6%)	

**RPS6KA4 Mutation**			
Present	0 (0.0%)	5 (1.3%)	1
Absent	73 (100.0%)	370 (98.7%)	
**RPS6KA6 Mutation**			
Present	0 (0.0%)	0 (0.0%)	1
Absent	73 (100.0%)	375 (100.0%)	
**RRAS Mutation**			
Present	0 (0.0%)	4 (1.1%)	1
Absent	73 (100.0%)	371 (98.9%)	
**RRAS2 Mutation**			
Present	0 (0.0%)	0 (0.0%)	1
Absent	73 (100.0%)	375 (100.0%)	
**SOS1 Mutation**			
Present	0 (0.0%)	7 (1.9%)	0.6049
Absent	73 (100.0%)	368 (98.1%)	
**TGFB2 Mutation**			
Present	0 (0.0%)	0 (0.0%)	1
Absent	73 (100.0%)	375 (100.0%)	
**TGFBR1 Mutation**			
Present	2 (2.7%)	8 (2.1%)	0.6698
Absent	71 (97.3%)	367 (97.9%)	
**TGFBR2 Mutation**			
Present	3 (4.1%)	11 (2.9%)	0.7102
Absent	70 (95.9%)	364 (97.1%)	
**TP53 Mutation**			
Present	57 (78.1%)	296 (78.9%)	0.995
Absent	16 (21.9%)	79 (21.1%)	
**TRAF2 Mutation**			
Present	1 (1.4%)	5 (1.3%)	1
Absent	72 (98.6%)	370 (98.7%)	

Table S10. - Comparison of Late-Onset Hispanic/Latino (H/L) versus Late-Onset Non-Hispanic White (NHW) Patients Treated with FOLFOX.

**MAPK Pathway**			
**Gene**	**Early-Onset Hispanic/Latino Not Treated with FOLFOX n (%)**	**Early-Onset NHW Not Treated with FOLFOX n (%)**	**p-value**
**ACVR1 Mutation**			
Present	2 (3.8%)	5 (1.7%)	0.2743
Absent	50 (96.2%)	297 (98.3%)	
**AKT1 Mutation**			
Present	2 (3.8%)	4 (1.3%)	0.2153
Absent	50 (96.2%)	298 (98.7%)	
**AKT2 Mutation**			
Present	0 (0.0%)	2 (0.7%)	1
Absent	52 (100.0%)	300 (99.3%)	
**AKT3 Mutation**			
Present	3 (5.8%)	9 (3.0%)	0.3954
Absent	49 (94.2%)	293 (97.0%)	
**BRAF Mutation**			
Present	4 (7.7%)	24 (7.9%)	1

Absent	48 (92.3%)	278 (92.1%)	
**CACNA1H Mutation**			
Present	0 (0.0%)	0 (0.0%)	1
Absent	52 (100.0%)	302 (100.0%)	
**CACNA2D1 Mutation**			
Present	0 (0.0%)	0 (0.0%)	1
Absent	52 (100.0%)	302 (100.0%)	
**CACNB1 Mutation**			
Present	0 (0.0%)	0 (0.0%)	1
Absent	52 (100.0%)	302 (100.0%)	
**CDC42 Mutation**			
Present	0 (0.0%)	2 (0.7%)	1
Absent	52 (100.0%)	300 (99.3%)	
**CRKL Mutation**			
Present	0 (0.0%)	3 (1.0%)	1
Absent	52 (100.0%)	299 (99.0%)	
**DAXX Mutation**			
Present	2 (3.8%)	6 (2.0%)	0.3331
Absent	50 (96.2%)	296 (98.0%)	
**DUSP4 Mutation**			
Present	0 (0.0%)	1 (0.3%)	1
Absent	52 (100.0%)	301 (99.7%)	
**EGFR Mutation**			
Present	3 (5.8%)	5 (1.7%)	0.09778
Absent	49 (94.2%)	297 (98.3%)	
**FGF13 Mutation**			
Present	0 (0.0%)	0 (0.0%)	1
Absent	52 (100.0%)	302 (100.0%)	
**FGF19 Mutation**			
Present	0 (0.0%)	4 (1.3%)	1
Absent	52 (100.0%)	298 (98.7%)	
**FGF3 Mutation**			
Present	1 (1.9%)	5 (1.7%)	1
Absent	51 (98.1%)	297 (98.3%)	
**FGF4 Mutation**			
Present	0 (0.0%)	5 (1.7%)	1
Absent	52 (100.0%)	297 (98.3%)	
**FGFR1 Mutation**			
Present	2 (3.8%)	6 (2.0%)	0.3331
Absent	50 (96.2%)	296 (98.0%)	
**FGFR2 Mutation**			
Present	4 (7.7%)	9 (3.0%)	0.1073
Absent	48 (92.3%)	293 (97.0%)	
**FGFR3 Mutation**			
Present	2 (3.8%)	11 (3.6%)	1
Absent	50 (96.2%)	291 (96.4%)	
**FGFR4 Mutation**			
Present	1 (1.9%)	5 (1.7%)	1
Absent	51 (98.1%)	297 (98.3%)	
**HRAS Mutation**			
Present	1 (1.9%)	2 (0.7%)	0.38
Absent	51 (98.1%)	300 (99.3%)	

**JUN Mutation**			
Present	0 (0.0%)	2 (0.7%)	1
Absent	52 (100.0%)	300 (99.3%)	
**KRAS Mutation**			
Present	18 (34.6%)	124 (41.1%)	0.47
Absent	34 (65.4%)	178 (58.9%)	
**MAP2K1 Mutation**			
Present	3 (5.8%)	4 (1.3%)	0.06807
Absent	49 (94.2%)	298 (98.7%)	
**MAP2K2 Mutation**			
Present	1 (1.9%)	1 (0.3%)	0.2726
Absent	51 (98.1%)	301 (99.7%)	
**MAP2K4 Mutation**			
Present	2 (3.8%)	9 (3.0%)	0.668
Absent	50 (96.2%)	293 (97.0%)	
**MAP3K1 Mutation**			
Present	4 (7.7%)	12 (4.0%)	0.2694
Absent	48 (92.3%)	290 (96.0%)	
**MAP3K13 Mutation**			
Present	0 (0.0%)	9 (3.0%)	0.3669
Absent	52 (100.0%)	293 (97.0%)	
**MAP3K14 Mutation**			
Present	0 (0.0%)	0 (0.0%)	1
Absent	52 (100.0%)	302 (100.0%)	
**MAPK1 Mutation**			
Present	2 (3.8%)	3 (1.0%)	0.158
Absent	50 (96.2%)	299 (99.0%)	
**MAPK3 Mutation**			
Present	3 (5.8%)	3 (1.0%)	**0.04335**
Absent	49 (94.2%)	299 (99.0%)	
**MAX Mutation**			
Present	0 (0.0%)	1 (0.3%)	1
Absent	52 (100.0%)	301 (99.7%)	
**MYC Mutation**			
Present	0 (0.0%)	1 (0.3%)	1
Absent	52 (100.0%)	301 (99.7%)	
**NF1 Mutation**			
Present	10 (19.2%)	18 (6.0%)	**0.002728**
Absent	42 (80.8%)	284 (94.0%)	
**NFATC2 Mutation**			
Present	0 (0.0%)	0 (0.0%)	1
Absent	52 (100.0%)	302 (100.0%)	
**NRAS Mutation**			
Present	3 (5.8%)	6 (2.0%)	0.1318
Absent	49 (94.2%)	296 (98.0%)	
**NTRK1 Mutation**			
Present	2 (3.8%)	9 (3.0%)	0.668
Absent	50 (96.2%)	293 (97.0%)	
**NTRK2 Mutation**			
Present	2 (3.8%)	8 (2.6%)	0.6456
Absent	50 (96.2%)	294 (97.4%)	

**PAK1 Mutation**			
Present	2 (3.8%)	3 (1.0%)	0.158
Absent	50 (96.2%)	299 (99.0%)	
**PDGFRA Mutation**			
Present	5 (9.6%)	16 (5.3%)	0.3684
Absent	47 (90.4%)	286 (94.7%)	
**PDGFRB Mutation**			
Present	3 (5.8%)	7 (2.3%)	0.1693
Absent	49 (94.2%)	295 (97.7%)	
**PLA2G3 Mutation**			
Present	0 (0.0%)	0 (0.0%)	1
Absent	52 (100.0%)	302 (100.0%)	
**RAC1 Mutation**			
Present	0 (0.0%)	3 (1.0%)	1
Absent	52 (100.0%)	299 (99.0%)	
**RAC2 Mutation**			
Present	0 (0.0%)	2 (0.7%)	1
Absent	52 (100.0%)	300 (99.3%)	
**RAF1 Mutation**			
Present	2 (3.8%)	9 (3.0%)	0.668
Absent	50 (96.2%)	293 (97.0%)	
**RASA1 Mutation**			
Present	3 (5.8%)	14 (4.6%)	0.7245
Absent	49 (94.2%)	288 (95.4%)	
**RPS6KA4 Mutation**			
Present	5 (9.6%)	8 (2.6%)	**0.03866**
Absent	47 (90.4%)	294 (97.4%)	
**RPS6KA6 Mutation**			
Present	0 (0.0%)	1 (0.3%)	1
Absent	52 (100.0%)	301 (99.7%)	
**RRAS Mutation**			
Present	1 (1.9%)	0 (0.0%)	0.1469
Absent	51 (98.1%)	302 (100.0%)	
**RRAS2 Mutation**			
Present	0 (0.0%)	4 (1.3%)	1
Absent	52 (100.0%)	298 (98.7%)	
**SOS1 Mutation**			
Present	1 (1.9%)	11 (3.6%)	1
Absent	51 (98.1%)	291 (96.4%)	
**TGFB2 Mutation**			
Present	0 (0.0%)	0 (0.0%)	1
Absent	52 (100.0%)	302 (100.0%)	
**TGFBR1 Mutation**			
Present	2 (3.8%)	9 (3.0%)	0.668
Absent	50 (96.2%)	293 (97.0%)	
**TGFBR2 Mutation**			
Present	6 (11.5%)	16 (5.3%)	0.1583
Absent	46 (88.5%)	286 (94.7%)	
**TP53 Mutation**			
Present	42 (80.8%)	228 (75.5%)	0.5163
Absent	10 (19.2%)	74 (24.5%)	

**TRAF2 Mutation**			
Present	0 (0.0%)	4 (1.3%)	1
Absent	52 (100.0%)	298 (98.7%)	

Table S11. - Comparison of Late-Onset Hispanic/Latino (H/L) versus Late-Onset Non-Hispanic White (NHW) Patients Not Treated with FOLFOX.

**MAPK Pathway**			
**Gene**	**Late-Onset Hispanic/Latino Treated with FOLFOX n (%)**	**Late-Onset NHW Treated with FOLFOX n (%)**	**p-value**
**ACVR1 Mutation**			
Present	0 (0.0%)	7 (0.8%)	1
Absent	91 (100.0%)	912 (99.2%)	
**AKT1 Mutation**			
Present	2 (2.2%)	16 (1.7%)	0.6731
Absent	89 (97.8%)	903 (98.3%)	
**AKT2 Mutation**			
Present	2 (2.2%)	6 (0.7%)	0.1574
Absent	89 (97.8%)	913 (99.3%)	
**AKT3 Mutation**			
Present	2 (2.2%)	9 (1.0%)	0.2603
Absent	89 (97.8%)	910 (99.0%)	
**BRAF Mutation**			
Present	15 (16.5%)	102 (11.1%)	0.1741
Absent	76 (83.5%)	817 (88.9%)	
**CACNA1H Mutation**			
Present	0 (0.0%)	0 (0.0%)	1
Absent	91 (100.0%)	919 (100.0%)	
**CACNA2D1 Mutation**			
Present	0 (0.0%)	1 (0.1%)	1
Absent	91 (100.0%)	918 (99.9%)	
**CACNB1 Mutation**			
Present	0 (0.0%)	1 (0.1%)	1
Absent	91 (100.0%)	918 (99.9%)	
**CDC42 Mutation**			
Present	1 (1.1%)	2 (0.2%)	0.2469
Absent	90 (98.9%)	917 (99.8%)	
**CRKL Mutation**			
Present	0 (0.0%)	1 (0.1%)	1
Absent	91 (100.0%)	918 (99.9%)	
**DAXX Mutation**			
Present	0 (0.0%)	14 (1.5%)	0.6284
Absent	91 (100.0%)	905 (98.5%)	
**DUSP4 Mutation**			
Present	1 (1.1%)	1 (0.1%)	0.1722
Absent	90 (98.9%)	918 (99.9%)	
**EGFR Mutation**			
Present	1 (1.1%)	22 (2.4%)	0.7141
Absent	90 (98.9%)	897 (97.6%)	
**FGF13 Mutation**			
Present	1 (1.1%)	0 (0.0%)	0.0901

Absent	90 (98.9%)	919 (100.0%)	
**FGF19 Mutation**			
Present	1 (1.1%)	5 (0.5%)	0.4333
Absent	90 (98.9%)	914 (99.5%)	
**FGF3 Mutation**			
Present	2 (2.2%)	13 (1.4%)	0.6379
Absent	89 (97.8%)	906 (98.6%)	
**FGF4 Mutation**			
Present	0 (0.0%)	1 (0.1%)	1
Absent	91 (100.0%)	918 (99.9%)	
**FGFR1 Mutation**			
Present	0 (0.0%)	15 (1.6%)	0.386
Absent	91 (100.0%)	904 (98.4%)	
**FGFR2 Mutation**			
Present	0 (0.0%)	8 (0.9%)	1
Absent	91 (100.0%)	911 (99.1%)	
**FGFR3 Mutation**			
Present	3 (3.3%)	16 (1.7%)	0.241
Absent	88 (96.7%)	903 (98.3%)	
**FGFR4 Mutation**			
Present	1 (1.1%)	17 (1.8%)	1
Absent	90 (98.9%)	902 (98.2%)	
**HRAS Mutation**			
Present	0 (0.0%)	4 (0.4%)	1
Absent	91 (100.0%)	915 (99.6%)	
**JUN Mutation**			
Present	0 (0.0%)	13 (1.4%)	0.6207
Absent	91 (100.0%)	906 (98.6%)	
**KRAS Mutation**			
Present	39 (42.9%)	403 (43.9%)	0.9428
Absent	52 (57.1%)	516 (56.1%)	
**MAP2K1 Mutation**			
Present	0 (0.0%)	12 (1.3%)	0.6152
Absent	91 (100.0%)	907 (98.7%)	
**MAP2K2 Mutation**			
Present	2 (2.2%)	7 (0.8%)	0.191
Absent	89 (97.8%)	912 (99.2%)	
**MAP2K4 Mutation**			
Present	1 (1.1%)	18 (2.0%)	1
Absent	90 (98.9%)	901 (98.0%)	
**MAP3K1 Mutation**			
Present	1 (1.1%)	27 (2.9%)	0.5044
Absent	90 (98.9%)	892 (97.1%)	
**MAP3K13 Mutation**			
Present	2 (2.2%)	16 (1.7%)	0.6731
Absent	89 (97.8%)	903 (98.3%)	
**MAP3K14 Mutation**			
Present	0 (0.0%)	0 (0.0%)	1
Absent	91 (100.0%)	919 (100.0%)	
**MAPK1 Mutation**			
Present	0 (0.0%)	1 (0.1%)	1
Absent	91 (100.0%)	918 (99.9%)	

**MAPK3 Mutation**			
Present	1 (1.1%)	4 (0.4%)	0.3769
Absent	90 (98.9%)	915 (99.6%)	
**MAX Mutation**			
Present	0 (0.0%)	7 (0.8%)	1
Absent	91 (100.0%)	912 (99.2%)	
**MYC Mutation**			
Present	0 (0.0%)	7 (0.8%)	1
Absent	91 (100.0%)	912 (99.2%)	
**NF1 Mutation**			
Present	2 (2.2%)	46 (5.0%)	0.3063
Absent	89 (97.8%)	873 (95.0%)	
**NFATC2 Mutation**			
Present	0 (0.0%)	1 (0.1%)	1
Absent	91 (100.0%)	918 (99.9%)	
**NRAS Mutation**			
Present	6 (6.6%)	36 (3.9%)	0.3449
Absent	85 (93.4%)	883 (96.1%)	
**NTRK1 Mutation**			
Present	2 (2.2%)	14 (1.5%)	0.6487
Absent	89 (97.8%)	905 (98.5%)	
**NTRK2 Mutation**			
Present	0 (0.0%)	16 (1.7%)	0.3866
Absent	91 (100.0%)	903 (98.3%)	
**PAK1 Mutation**			
Present	0 (0.0%)	8 (0.9%)	1
Absent	91 (100.0%)	911 (99.1%)	
**PDGFRA Mutation**			
Present	0 (0.0%)	18 (2.0%)	0.3962
Absent	91 (100.0%)	901 (98.0%)	
**PDGFRB Mutation**			
Present	0 (0.0%)	16 (1.7%)	0.3866
Absent	91 (100.0%)	903 (98.3%)	
**PLA2G3 Mutation**			
Present	0 (0.0%)	0 (0.0%)	1
Absent	91 (100.0%)	919 (100.0%)	
**RAC1 Mutation**			
Present	0 (0.0%)	0 (0.0%)	1
Absent	91 (100.0%)	919 (100.0%)	
**RAC2 Mutation**			
Present	0 (0.0%)	0 (0.0%)	1
Absent	91 (100.0%)	919 (100.0%)	
**RAF1 Mutation**			
Present	2 (2.2%)	8 (0.9%)	0.2255
Absent	89 (97.8%)	911 (99.1%)	
**RASA1 Mutation**			
Present	4 (4.4%)	19 (2.1%)	0.1454
Absent	87 (95.6%)	900 (97.9%)	
**RPS6KA4 Mutation**			
Present	3 (3.3%)	17 (1.8%)	0.4148
Absent	88 (96.7%)	902 (98.2%)	

**RPS6KA6 Mutation**			
Present	0 (0.0%)	0 (0.0%)	1
Absent	91 (100.0%)	919 (100.0%)	
**RRAS Mutation**			
Present	0 (0.0%)	2 (0.2%)	1
Absent	91 (100.0%)	917 (99.8%)	
**RRAS2 Mutation**			
Present	1 (1.1%)	5 (0.5%)	0.4333
Absent	90 (98.9%)	914 (99.5%)	
**SOS1 Mutation**			
Present	3 (3.3%)	8 (0.9%)	0.06862
Absent	88 (96.7%)	911 (99.1%)	
**TGFB2 Mutation**			
Present	0 (0.0%)	1 (0.1%)	1
Absent	91 (100.0%)	918 (99.9%)	
**TGFBR1 Mutation**			
Present	3 (3.3%)	13 (1.4%)	0.1685
Absent	88 (96.7%)	906 (98.6%)	
**TGFBR2 Mutation**			
Present	5 (5.5%)	38 (4.1%)	0.7334
Absent	86 (94.5%)	881 (95.9%)	
**TP53 Mutation**			
Present	71 (78.0%)	675 (73.4%)	0.4111
Absent	20 (22.0%)	244 (26.6%)	
**TRAF2 Mutation**			
Present	0 (0.0%)	10 (1.1%)	1
Absent	91 (100.0%)	909 (98.9%)	

Table S12. - Comparison of Late-Onset Hispanic/Latino (H/L) versus Late-Onset Non-Hispanic White (NHW) Patients Not Treated with FOLFOX.

**MAPK Pathway**			
**Gene**	**Late-Onset Hispanic/Latino Not Treated with FOLFOX n (%)**	**Late-Onset NHW Not Treated with FOLFOX n (%)**	**p-value**
**ACVR1 Mutation**			
Present	0 (0.0%)	8 (1.2%)	1
Absent	50 (100.0%)	645 (98.8%)	
**AKT1 Mutation**			
Present	2 (4.0%)	19 (2.9%)	0.6558
Absent	48 (96.0%)	634 (97.1%)	
**AKT2 Mutation**			
Present	0 (0.0%)	12 (1.8%)	1
Absent	50 (100.0%)	641 (98.2%)	
**AKT3 Mutation**			
Present	2 (4.0%)	12 (1.8%)	0.2621
Absent	48 (96.0%)	641 (98.2%)	
**BRAF Mutation**			
Present	8 (16.0%)	93 (14.2%)	0.8947
Absent	42 (84.0%)	560 (85.8%)	
**CACNA1H Mutation**			
Present	0 (0.0%)	1 (0.2%)	1

Absent	50 (100.0%)	652 (99.8%)	
**CACNA2D1 Mutation**			
Present	0 (0.0%)	0 (0.0%)	1
Absent	50 (100.0%)	653 (100.0%)	
**CACNB1 Mutation**			
Present	0 (0.0%)	0 (0.0%)	1
Absent	50 (100.0%)	653 (100.0%)	
**CDC42 Mutation**			
Present	1 (2.0%)	2 (0.3%)	0.1988
Absent	49 (98.0%)	651 (99.7%)	
**CRKL Mutation**			
Present	0 (0.0%)	8 (1.2%)	1
Absent	50 (100.0%)	645 (98.8%)	
**DAXX Mutation**			
Present	1 (2.0%)	13 (2.0%)	1
Absent	49 (98.0%)	640 (98.0%)	
**DUSP4 Mutation**			
Present	0 (0.0%)	6 (0.9%)	1
Absent	50 (100.0%)	647 (99.1%)	
**EGFR Mutation**			
Present	3 (6.0%)	24 (3.7%)	0.4311
Absent	47 (94.0%)	629 (96.3%)	
**FGF13 Mutation**			
Present	0 (0.0%)	0 (0.0%)	1
Absent	50 (100.0%)	653 (100.0%)	
**FGF19 Mutation**			
Present	2 (4.0%)	5 (0.8%)	0.08273
Absent	48 (96.0%)	648 (99.2%)	
**FGF3 Mutation**			
Present	0 (0.0%)	14 (2.1%)	0.6146
Absent	50 (100.0%)	639 (97.9%)	
**FGF4 Mutation**			
Present	0 (0.0%)	4 (0.6%)	1
Absent	50 (100.0%)	649 (99.4%)	
**FGFR1 Mutation**			
Present	0 (0.0%)	16 (2.5%)	0.62
Absent	50 (100.0%)	637 (97.5%)	
**FGFR2 Mutation**			
Present	1 (2.0%)	11 (1.7%)	1
Absent	49 (98.0%)	642 (98.3%)	
**FGFR3 Mutation**			
Present	2 (4.0%)	19 (2.9%)	0.6558
Absent	48 (96.0%)	634 (97.1%)	
**FGFR4 Mutation**			
Present	1 (2.0%)	22 (3.4%)	1
Absent	49 (98.0%)	631 (96.6%)	
**HRAS Mutation**			
Present	1 (2.0%)	6 (0.9%)	0.4047
Absent	49 (98.0%)	647 (99.1%)	
**JUN Mutation**			
Present	0 (0.0%)	21 (3.2%)	0.3898
Absent	50 (100.0%)	632 (96.8%)	

**KRAS Mutation**			
Present	20 (40.0%)	279 (42.7%)	0.8202
Absent	30 (60.0%)	374 (57.3%)	
**MAP2K1 Mutation**			
Present	0 (0.0%)	10 (1.5%)	1
Absent	50 (100.0%)	643 (98.5%)	
**MAP2K2 Mutation**			
Present	1 (2.0%)	10 (1.5%)	0.5585
Absent	49 (98.0%)	643 (98.5%)	
**MAP2K4 Mutation**			
Present	1 (2.0%)	17 (2.6%)	1
Absent	49 (98.0%)	636 (97.4%)	
**MAP3K1 Mutation**			
Present	1 (2.0%)	31 (4.7%)	0.7202
Absent	49 (98.0%)	622 (95.3%)	
**MAP3K13 Mutation**			
Present	0 (0.0%)	15 (2.3%)	0.6164
Absent	50 (100.0%)	638 (97.7%)	
**MAP3K14 Mutation**			
Present	0 (0.0%)	0 (0.0%)	1
Absent	50 (100.0%)	653 (100.0%)	
**MAPK1 Mutation**			
Present	0 (0.0%)	6 (0.9%)	1
Absent	50 (100.0%)	647 (99.1%)	
**MAPK3 Mutation**			
Present	0 (0.0%)	5 (0.8%)	1
Absent	50 (100.0%)	648 (99.2%)	
**MAX Mutation**			
Present	0 (0.0%)	5 (0.8%)	1
Absent	50 (100.0%)	648 (99.2%)	
**MYC Mutation**			
Present	2 (4.0%)	9 (1.4%)	0.1809
Absent	48 (96.0%)	644 (98.6%)	
**NF1 Mutation**			
Present	2 (4.0%)	44 (6.7%)	0.7642
Absent	48 (96.0%)	609 (93.3%)	
**NFATC2 Mutation**			
Present	0 (0.0%)	0 (0.0%)	1
Absent	50 (100.0%)	653 (100.0%)	
**NRAS Mutation**			
Present	3 (6.0%)	19 (2.9%)	0.2011
Absent	47 (94.0%)	634 (97.1%)	
**NTRK1 Mutation**			
Present	2 (4.0%)	14 (2.1%)	0.3169
Absent	48 (96.0%)	639 (97.9%)	
**NTRK2 Mutation**			
Present	3 (6.0%)	9 (1.4%)	**0.0472**
Absent	47 (94.0%)	644 (98.6%)	
**PAK1 Mutation**			
Present	0 (0.0%)	6 (0.9%)	1
Absent	50 (100.0%)	647 (99.1%)	

**PDGFRA Mutation**			
Present	0 (0.0%)	24 (3.7%)	0.4049
Absent	50 (100.0%)	629 (96.3%)	
**PDGFRB Mutation**			
Present	3 (6.0%)	16 (2.5%)	0.1466
Absent	47 (94.0%)	637 (97.5%)	
**PLA2G3 Mutation**			
Present	0 (0.0%)	1 (0.2%)	1
Absent	50 (100.0%)	652 (99.8%)	
**RAC1 Mutation**			
Present	0 (0.0%)	3 (0.5%)	1
Absent	50 (100.0%)	650 (99.5%)	
**RAC2 Mutation**			
Present	1 (2.0%)	3 (0.5%)	0.256
Absent	49 (98.0%)	650 (99.5%)	
**RAF1 Mutation**			
Present	1 (2.0%)	19 (2.9%)	1
Absent	49 (98.0%)	634 (97.1%)	
**RASA1 Mutation**			
Present	1 (2.0%)	24 (3.7%)	1
Absent	49 (98.0%)	629 (96.3%)	
**RPS6KA4 Mutation**			
Present	3 (6.0%)	34 (5.2%)	0.7411
Absent	47 (94.0%)	619 (94.8%)	
**RPS6KA6 Mutation**			
Present	0 (0.0%)	0 (0.0%)	1
Absent	50 (100.0%)	653 (100.0%)	
**RRAS Mutation**			
Present	0 (0.0%)	8 (1.2%)	1
Absent	50 (100.0%)	645 (98.8%)	
**RRAS2 Mutation**			
Present	2 (4.0%)	3 (0.5%)	**0.04318**
Absent	48 (96.0%)	650 (99.5%)	
**SOS1 Mutation**			
Present	0 (0.0%)	15 (2.3%)	0.6164
Absent	50 (100.0%)	638 (97.7%)	
**TGFB2 Mutation**			
Present	0 (0.0%)	0 (0.0%)	1
Absent	50 (100.0%)	653 (100.0%)	
**TGFBR1 Mutation**			
Present	1 (2.0%)	12 (1.8%)	1
Absent	49 (98.0%)	641 (98.2%)	
**TGFBR2 Mutation**			
Present	3 (6.0%)	46 (7.0%)	1
Absent	47 (94.0%)	607 (93.0%)	
**TP53 Mutation**			
Present	36 (72.0%)	445 (68.1%)	0.684
Absent	14 (28.0%)	208 (31.9%)	
**TRAF2 Mutation**			
Present	1 (2.0%)	15 (2.3%)	1
Absent	49 (98.0%)	638 (97.7%)	

Table S12. Distribution of MAPK Pathway Mutation Classes by Ancestry, Age at Diagnosis, and FOLFOX Exposure in Colorectal Cancer**.** This supplementary table details the spectrum of somatic mutation classes observed in MAPK signaling pathway genes across colorectal cancer subgroups stratified by ancestral background (Hispanic/Latino [H/L] vs. non-Hispanic White [NHW]), age of onset (early-onset vs. late-onset), and FOLFOX chemotherapy status (treated vs. not treated). Mutation categories include missense substitutions, nonsense mutations, frameshift insertions and deletions, in-frame insertions/deletions, splice-site and splice-region variants, nonstop mutations, and translation start-site alterations. For each subgroup, percentages reflect the relative contribution of each mutation class to the total number of MAPK pathway alterations identified within that stratum. By comparing mutation-type distributions across demographic and treatment-defined cohorts, this table provides insight into how ancestry, age at diagnosis, and chemotherapy exposure influence the qualitative nature of MAPK pathway genomic disruption, complementing gene-level frequency and pathway-level analyses presented in the main text.

	Hispanic/Latino Samples				Non-Hispanic White Samples			
	Early-Onset		Late-Onset		Early-Onset		Late-Onset	
	Treated with FOLFOX	Not Treated with FOLFOX	Treated with FOLFOX	Not Treated with FOLFOX	Treated with FOLFOX	Not Treated with FOLFOX	Treated with FOLFOX	Not Treated with FOLFOX
**ACVR1**								
Frame Shift Insertion	0.0%	0.0%	0.0%	0.0%	25.0%	0.0%	0.0%	0.0%
Missense Mutation	100.0%	100.0%	0.0%	0.0%	75.0%	83.3%	100.0%	100.0%
Nonsense Mutation	0.0%	0.0%	0.0%	0.0%	0.0%	16.7%	0.0%	0.0%
**AKT1**								
Frame Shift Deletion	0.0%	0.0%	0.0%	0.0%	11.1%	0.0%	0.0%	0.0%
In Frame Deletion	25.0%	0.0%	0.0%	0.0%	0.0%	0.0%	0.0%	20.0%
Missense Mutation	75.0%	100.0%	100.0%	100.0%	88.9%	100.0%	100.0%	80.0%
**AKT2**								
Frame Shift Deletion	0.0%	0.0%	50.0%	0.0%	0.0%	0.0%	0.0%	7.7%
Missense Mutation	0.0%	0.0%	50.0%	100.0%	100.0%	100.0%	100.0%	92.3%
**AKT3**								
Frame Shift Deletion	0.0%	0.0%	50.0%	0.0%	0.0%	0.0%	11.1%	0.0%
Missense Mutation	100.0%	33.3%	50.0%	50.0%	100.0%	73.3%	66.7%	100.0%
Nonsense Mutation	0.0%	66.7%	0.0%	50.0%	0.0%	26.7%	22.2%	0.0%
**BRAF**								
Frame Shift Deletion	0.0%	0.0%	0.0%	0.0%	0.0%	3.6%	3.8%	1.0%
Frame Shift Insertion	0.0%	0.0%	0.0%	0.0%	0.0%	0.0%	0.9%	4.0%
In Frame Deletion	0.0%	0.0%	0.0%	0.0%	0.0%	3.6%	0.0%	0.0%
Missense Mutation	100.0%	100.0%	100.0%	100.0%	100.0%	82.1%	95.3%	94.9%
Nonsense Mutation	0.0%	0.0%	0.0%	0.0%	0.0%	7.1%	0.0%	0.0%
Splice Site	0.0%	0.0%	0.0%	0.0%	0.0%	3.6%	0.0%	0.0%
**CACNA1H**								
Missense Mutation	0.0%	0.0%	0.0%	0.0%	0.0%	0.0%	0.0%	100.0%
**CACNA2D1**								
Splice Site	0.0%	0.0%	0.0%	0.0%	0.0%	0.0%	100.0%	0.0%
**CACNB1**								
Missense Mutation	0.0%	0.0%	0.0%	0.0%	0.0%	0.0%	100.0%	0.0%
**CDC42**								
Frame Shift Deletion	0.0%	0.0%	0.0%	0.0%	0.0%	50.0%	50.0%	50.0%
Missense Mutation	0.0%	0.0%	100.0%	100.0%	0.0%	50.0%	50.0%	50.0%
**CRKL**								
Missense Mutation	0.0%	0.0%	0.0%	0.0%	100.0%	100.0%	100.0%	87.5%
Nonsense Mutation	0.0%	0.0%	0.0%	0.0%	0.0%	0.0%	0.0%	12.5%
**DUSP3**								
Frame Shift Deletion	0.0%	50.0%	0.0%	0.0%	0.0%	0.0%	7.1%	0.0%
Frame Shift Insertion	0.0%	0.0%	0.0%	0.0%	0.0%	14.3%	0.0%	0.0%
In Frame Deletion	0.0%	0.0%	0.0%	0.0%	0.0%	0.0%	0.0%	7.7%
Missense Mutation	100.0%	50.0%	0.0%	100.0%	100.0%	85.7%	85.7%	92.3%
Splice Site	0.0%	0.0%	0.0%	0.0%	0.0%	0.0%	7.1%	0.0%
**DUSP4**								
Frame Shift Deletion	0.0%	0.0%	0.0%	0.0%	100.0%	0.0%	0.0%	16.7%
Missense Mutation	0.0%	0.0%	100.0%	0.0%	0.0%	100.0%	100.0%	83.3%
**EGFR**								
Frame Shift Deletion	0.0%	33.3%	0.0%	0.0%	0.0%	0.0%	0.0%	0.0%
In Frame Deletion	0.0%	0.0%	0.0%	0.0%	0.0%	0.0%	7.7%	0.0%
In Frame Insertion	0.0%	0.0%	0.0%	0.0%	0.0%	0.0%	3.8%	0.0%
Missense Mutation	100.0%	33.3%	100.0%	100.0%	100.0%	100.0%	76.9%	96.2%
Nonsense Mutation	0.0%	0.0%	0.0%	0.0%	0.0%	0.0%	3.8%	3.8%
Splice Site	0.0%	33.3%	0.0%	0.0%	0.0%	0.0%	7.7%	0.0%
**FGF13**								
Nonsense Mutation	0.0%	0.0%	100.0%	0.0%	0.0%	0.0%	0.0%	0.0%
**FGF19**								
Frame Shift Deletion	0.0%	0.0%	0.0%	50.0%	0.0%	0.0%	0.0%	0.0%
Missense Mutation	100.0%	0.0%	100.0%	50.0%	100.0%	100.0%	83.3%	100.0%
Splice Site	0.0%	0.0%	0.0%	0.0%	0.0%	0.0%	16.7%	0.0%
**FGF3**								
Frame Shift Deletion	0.0%	100.0%	0.0%	0.0%	0.0%	0.0%	0.0%	7.1%
Missense Mutation	0.0%	0.0%	100.0%	0.0%	100.0%	100.0%	92.9%	85.7%
Nonsense Mutation	0.0%	0.0%	0.0%	0.0%	0.0%	0.0%	7.1%	7.1%
**FGF4**								
Missense Mutation	0.0%	0.0%	0.0%	0.0%	0.0%	100.0%	100.0%	100.0%
**FGFR1**								
Frame Shift Deletion	0.0%	0.0%	0.0%	0.0%	0.0%	14.3%	0.0%	5.9%
Missense Mutation	0.0%	100.0%	0.0%	0.0%	90.0%	85.7%	93.8%	88.2%
Splice Site	0.0%	0.0%	0.0%	0.0%	10.0%	0.0%	6.3%	5.9%
**FGFR2**								
Frame Shift Deletion	0.0%	0.0%	0.0%	0.0%	20.0%	0.0%	11.1%	0.0%
Frame Shift Insertion	0.0%	0.0%	0.0%	0.0%	0.0%	0.0%	11.1%	0.0%
Missense Mutation	0.0%	75.0%	0.0%	100.0%	40.0%	80.0%	77.8%	84.6%
Nonsense Mutation	0.0%	25.0%	0.0%	0.0%	40.0%	20.0%	0.0%	7.7%
Splice Site	0.0%	0.0%	0.0%	0.0%	0.0%	0.0%	0.0%	7.7%
**FGFR3**								
Frame Shift Deletion	0.0%	0.0%	0.0%	0.0%	40.0%	0.0%	5.0%	9.5%
Frame Shift Insertion	0.0%	0.0%	0.0%	0.0%	0.0%	15.4%	0.0%	9.5%
Missense Mutation	100.0%	100.0%	100.0%	100.0%	60.0%	76.9%	95.0%	76.2%
Nonsense Mutation	0.0%	0.0%	0.0%	0.0%	0.0%	7.7%	0.0%	0.0%
Splice Site	0.0%	0.0%	0.0%	0.0%	0.0%	0.0%	0.0%	4.8%
**FGFR4**								
Frame Shift Deletion	100.0%	0.0%	0.0%	0.0%	16.7%	0.0%	0.0%	20.0%
Frame Shift Insertion	0.0%	0.0%	0.0%	0.0%	0.0%	0.0%	0.0%	4.0%
Missense Mutation	0.0%	100.0%	100.0%	100.0%	83.3%	100.0%	100.0%	72.0%
Splice Site	0.0%	0.0%	0.0%	0.0%	0.0%	0.0%	0.0%	4.0%
**HRAS**								
Missense Mutation	100.0%	100.0%	0.0%	100.0%	100.0%	100.0%	100.0%	100.0%
**JUN**								
Frame Shift Deletion	40.0%	0.0%	0.0%	0.0%	25.0%	50.0%	46.2%	38.1%
Frame Shift Insertion	40.0%	0.0%	0.0%	0.0%	25.0%	50.0%	30.8%	14.3%
In Frame Deletion	0.0%	0.0%	0.0%	0.0%	0.0%	0.0%	7.7%	0.0%
Missense Mutation	20.0%	0.0%	0.0%	0.0%	41.7%	0.0%	15.4%	33.3%
Nonsense Mutation	0.0%	0.0%	0.0%	0.0%	8.3%	0.0%	0.0%	4.8%
Translation Start Site	0.0%	0.0%	0.0%	0.0%	0.0%	0.0%	0.0%	9.5%
**KRAS**								
Frame Shift Insertion	0.0%	0.0%	0.0%	0.0%	0.0%	0.0%	0.5%	0.0%
In Frame Insertion	0.0%	0.0%	0.0%	0.0%	0.0%	0.0%	1.0%	0.0%
Missense Mutation	100.0%	100.0%	100.0%	100.0%	99.4%	100.0%	98.5%	100.0%
Nonsense Mutation	0.0%	0.0%	0.0%	0.0%	0.6%	0.0%	0.0%	0.0%
**MAP2K1**								
Frame Shift Deletion	0.0%	0.0%	0.0%	0.0%	0.0%	0.0%	7.7%	20.0%
In Frame Deletion	0.0%	0.0%	0.0%	0.0%	0.0%	0.0%	0.0%	30.0%
Missense Mutation	100.0%	100.0%	0.0%	0.0%	100.0%	100.0%	92.3%	50.0%
**MAP2K2**								
Frame Shift Insertion	0.0%	0.0%	0.0%	0.0%	33.3%	0.0%	0.0%	0.0%
Missense Mutation	100.0%	100.0%	100.0%	100.0%	66.7%	100.0%	87.5%	100.0%
Splice Site	0.0%	0.0%	0.0%	0.0%	0.0%	0.0%	12.5%	0.0%
**MAP2K4**								
Frame Shift Deletion	0.0%	0.0%	0.0%	0.0%	14.3%	0.0%	15.0%	5.0%
Frame Shift Insertion	0.0%	0.0%	0.0%	0.0%	0.0%	0.0%	5.0%	5.0%
In Frame Deletion	0.0%	0.0%	0.0%	0.0%	0.0%	9.1%	0.0%	5.0%
Missense Mutation	33.3%	50.0%	100.0%	100.0%	42.9%	45.5%	70.0%	55.0%
Nonsense Mutation	33.3%	50.0%	0.0%	0.0%	42.9%	27.3%	10.0%	15.0%
Splice Site	33.3%	0.0%	0.0%	0.0%	0.0%	18.2%	0.0%	15.0%
**MAP3K1**								
Frame Shift Deletion	0.0%	0.0%	0.0%	0.0%	7.7%	7.7%	9.4%	5.7%
Frame Shift Insertion	0.0%	16.7%	0.0%	0.0%	0.0%	0.0%	6.3%	5.7%
In Frame Deletion	0.0%	0.0%	0.0%	0.0%	7.7%	0.0%	6.3%	0.0%
Missense Mutation	100.0%	83.3%	100.0%	100.0%	61.5%	69.2%	53.1%	68.6%
Nonsense Mutation	0.0%	0.0%	0.0%	0.0%	15.4%	23.1%	12.5%	17.1%
Splice Site	0.0%	0.0%	0.0%	0.0%	7.7%	0.0%	12.5%	2.9%
**MAP3K13**								
Frame Shift Deletion	0.0%	0.0%	0.0%	0.0%	0.0%	18.2%	6.3%	5.9%
Frame Shift Insertion	0.0%	0.0%	0.0%	0.0%	0.0%	0.0%	0.0%	5.9%
In Frame Deletion	0.0%	0.0%	0.0%	0.0%	0.0%	9.1%	0.0%	0.0%
Missense Mutation	100.0%	0.0%	100.0%	0.0%	66.7%	54.5%	87.5%	76.5%
Nonsense Mutation	0.0%	0.0%	0.0%	0.0%	33.3%	18.2%	6.3%	11.8%
**MAP3K14**								
Splice Site	0.0%	0.0%	0.0%	0.0%	100.0%	0.0%	0.0%	0.0%
**MAPK1**								
In Frame Deletion	0.0%	0.0%	0.0%	0.0%	33.3%	0.0%	0.0%	16.7%
Missense Mutation	0.0%	100.0%	0.0%	0.0%	66.7%	100.0%	0.0%	83.3%
Nonsense Mutation	0.0%	0.0%	0.0%	0.0%	0.0%	0.0%	100.0%	0.0%
**MAPK3**								
In Frame Deletion	0.0%	0.0%	0.0%	0.0%	0.0%	0.0%	0.0%	20.0%
Missense Mutation	100.0%	100.0%	100.0%	0.0%	100.0%	100.0%	80.0%	80.0%
Nonsense Mutation	0.0%	0.0%	0.0%	0.0%	0.0%	0.0%	20.0%	0.0%
**MAX**								
Frame Shift Deletion	0.0%	0.0%	0.0%	0.0%	0.0%	0.0%	28.6%	0.0%
Missense Mutation	0.0%	0.0%	0.0%	0.0%	100.0%	0.0%	71.4%	100.0%
Nonsense Mutation	0.0%	0.0%	0.0%	0.0%	0.0%	100.0%	0.0%	0.0%
**MYC**								
In Frame Deletion	0.0%	0.0%	0.0%	0.0%	50.0%	0.0%	0.0%	0.0%
Missense Mutation	0.0%	0.0%	0.0%	100.0%	50.0%	100.0%	87.5%	100.0%
Nonstop Mutation	0.0%	0.0%	0.0%	0.0%	0.0%	0.0%	12.5%	0.0%
**NF1**								
Frame Shift Deletion	25.0%	13.6%	33.3%	0.0%	5.3%	7.7%	9.4%	3.4%
Frame Shift Insertion	0.0%	9.1%	0.0%	0.0%	10.5%	15.4%	7.5%	5.1%
In Frame Insertion	0.0%	0.0%	0.0%	0.0%	0.0%	0.0%	0.0%	1.7%
Missense Mutation	50.0%	59.1%	33.3%	0.0%	47.4%	69.2%	56.6%	66.1%
Nonsense Mutation	25.0%	9.1%	0.0%	0.0%	31.6%	3.8%	15.1%	20.3%
Splice Site	0.0%	9.1%	33.3%	100.0%	5.3%	3.8%	11.3%	3.4%
**NFATC2**								
Missense Mutation	0.0%	0.0%	0.0%	0.0%	0.0%	0.0%	100.0%	0.0%
**NRAS**								
Missense Mutation	100.0%	100.0%	100.0%	100.0%	100.0%	100.0%	100.0%	100.0%
**NTRK1**								
Frame Shift Deletion	0.0%	0.0%	0.0%	33.3%	0.0%	9.1%	0.0%	0.0%
Frame Shift Insertion	0.0%	0.0%	0.0%	0.0%	10.0%	0.0%	0.0%	0.0%
Missense Mutation	100.0%	100.0%	100.0%	66.7%	90.0%	90.9%	100.0%	81.3%
Nonsense Mutation	0.0%	0.0%	0.0%	0.0%	0.0%	0.0%	0.0%	18.8%
**NTRK2**								
Missense Mutation	100.0%	100.0%	0.0%	100.0%	100.0%	88.9%	100.0%	80.0%
Nonsense Mutation	0.0%	0.0%	0.0%	0.0%	0.0%	11.1%	0.0%	20.0%
**PAK1**								
Frame Shift Insertion	0.0%	0.0%	0.0%	0.0%	14.3%	0.0%	11.1%	16.7%
Missense Mutation	0.0%	100.0%	0.0%	0.0%	71.4%	100.0%	88.9%	83.3%
Splice Site	0.0%	0.0%	0.0%	0.0%	14.3%	0.0%	0.0%	0.0%
**PDGFRA**								
Frame Shift Deletion	0.0%	0.0%	0.0%	0.0%	6.3%	0.0%	0.0%	0.0%
Frame Shift Insertion	0.0%	0.0%	0.0%	0.0%	0.0%	0.0%	5.3%	3.3%
Missense Mutation	100.0%	100.0%	0.0%	0.0%	81.3%	81.5%	89.5%	73.3%
Nonsense Mutation	0.0%	0.0%	0.0%	0.0%	6.3%	11.1%	5.3%	16.7%
Splice Site	0.0%	0.0%	0.0%	0.0%	6.3%	7.4%	0.0%	6.7%
**PDGFRB**								
Frame Shift Deletion	0.0%	0.0%	0.0%	0.0%	0.0%	0.0%	11.8%	6.3%
Frame Shift Insertion	0.0%	0.0%	0.0%	0.0%	0.0%	0.0%	11.8%	6.3%
In Frame Deletion	0.0%	0.0%	0.0%	0.0%	0.0%	0.0%	0.0%	6.3%
Missense Mutation	300.0%	400.0%	0.0%	100.0%	100.0%	90.0%	64.7%	81.3%
Nonsense Mutation	100.0%	0.0%	0.0%	0.0%	0.0%	0.0%	11.8%	0.0%
Splice Site	0.0%	0.0%	0.0%	0.0%	0.0%	10.0%	0.0%	0.0%
**PLA2G3**								
Missense Mutation	0.0%	0.0%	0.0%	0.0%	0.0%	0.0%	0.0%	100.0%
**RAC1**								
Missense Mutation	0.0%	0.0%	0.0%	0.0%	100.0%	100.0%	0.0%	100.0%
**RAC2**								
Missense Mutation	0.0%	0.0%	0.0%	100.0%	100.0%	100.0%	0.0%	100.0%
**RAF1**								
Frame Shift Deletion	0.0%	0.0%	50.0%	0.0%	0.0%	0.0%	0.0%	9.5%
Frame Shift Insertion	0.0%	0.0%	0.0%	0.0%	0.0%	0.0%	12.5%	4.8%
Missense Mutation	100.0%	100.0%	50.0%	100.0%	88.9%	88.9%	75.0%	81.0%
Nonsense Mutation	0.0%	0.0%	0.0%	0.0%	11.1%	11.1%	0.0%	4.8%
Splice Site	0.0%	0.0%	0.0%	0.0%	0.0%	0.0%	12.5%	0.0%
**RASA1**								
Frame Shift Deletion	100.0%	50.0%	50.0%	0.0%	0.0%	4.8%	17.4%	14.7%
Frame Shift Insertion	0.0%	0.0%	25.0%	0.0%	7.7%	0.0%	8.7%	5.9%
In Frame Deletion	0.0%	0.0%	0.0%	0.0%	0.0%	0.0%	0.0%	2.9%
Missense Mutation	0.0%	25.0%	25.0%	0.0%	61.5%	71.4%	56.5%	61.8%
Nonsense Mutation	0.0%	25.0%	0.0%	0.0%	30.8%	14.3%	13.0%	8.8%
Splice Site	0.0%	0.0%	0.0%	100.0%	0.0%	9.5%	4.3%	5.9%
**RPS6KA4**								
Frame Shift Deletion	0.0%	20.0%	0.0%	33.3%	33.3%	25.0%	25.0%	21.6%
Frame Shift Insertion	0.0%	0.0%	0.0%	0.0%	0.0%	0.0%	0.0%	2.7%
In Frame Deletion	0.0%	0.0%	0.0%	0.0%	0.0%	0.0%	5.0%	0.0%
Missense Mutation	0.0%	80.0%	100.0%	66.7%	66.7%	75.0%	65.0%	75.7%
Nonsense Mutation	0.0%	0.0%	0.0%	0.0%	0.0%	0.0%	5.0%	0.0%
**RPS6KA6**								
Missense Mutation	0.0%	0.0%	0.0%	0.0%	0.0%	100.0%	0.0%	0.0%
**RRAS**								
Frame Shift Deletion	0.0%	0.0%	0.0%	0.0%	0.0%	0.0%	0.0%	12.5%
Missense Mutation	0.0%	100.0%	0.0%	0.0%	100.0%	0.0%	100.0%	75.0%
Splice Site	0.0%	0.0%	0.0%	0.0%	0.0%	0.0%	0.0%	12.5%
**RRAS2**								
Frame Shift Deletion	0.0%	0.0%	100.0%	0.0%	0.0%	0.0%	0.0%	0.0%
In Frame Deletion	0.0%	0.0%	0.0%	50.0%	0.0%	0.0%	0.0%	0.0%
Missense Mutation	0.0%	0.0%	0.0%	50.0%	0.0%	100.0%	100.0%	100.0%
**SOS1**								
Frame Shift Deletion	0.0%	0.0%	0.0%	0.0%	11.1%	8.3%	25.0%	6.7%
Frame Shift Insertion	0.0%	0.0%	0.0%	0.0%	0.0%	0.0%	0.0%	6.7%
Missense Mutation	0.0%	0.0%	100.0%	0.0%	66.7%	75.0%	75.0%	80.0%
Nonsense Mutation	0.0%	100.0%	0.0%	0.0%	22.2%	16.7%	0.0%	6.7%
**TGFB2**								
Missense Mutation	0.0%	0.0%	0.0%	0.0%	0.0%	0.0%	100.0%	0.0%
**TGFBR1**								
Frame Shift Deletion	0.0%	0.0%	0.0%	0.0%	10.0%	0.0%	0.0%	14.3%
In Frame Deletion	0.0%	0.0%	0.0%	0.0%	0.0%	0.0%	6.3%	7.1%
Missense Mutation	66.7%	100.0%	100.0%	100.0%	50.0%	81.8%	81.3%	71.4%
Nonsense Mutation	0.0%	0.0%	0.0%	0.0%	40.0%	18.2%	12.5%	7.1%
Translation Start Site	33.3%	0.0%	0.0%	0.0%	0.0%	0.0%	0.0%	0.0%
**TGFBR2**								
Frame Shift Deletion	0.0%	66.7%	40.0%	33.3%	27.3%	38.9%	23.3%	45.1%
Frame Shift Insertion	0.0%	0.0%	0.0%	0.0%	0.0%	5.6%	4.7%	3.9%
In Frame Deletion	0.0%	0.0%	0.0%	0.0%	0.0%	5.6%	2.3%	3.9%
In Frame Insertion	0.0%	0.0%	0.0%	0.0%	9.1%	0.0%	0.0%	0.0%
Missense Mutation	100.0%	33.3%	60.0%	66.7%	63.6%	50.0%	60.5%	39.2%
Nonsense Mutation	0.0%	0.0%	0.0%	0.0%	0.0%	0.0%	4.7%	3.9%
Splice Site	0.0%	0.0%	0.0%	0.0%	0.0%	0.0%	4.7%	3.9%
**TP53**								
Frame Shift Deletion	6.6%	2.1%	15.3%	10.0%	8.3%	7.6%	6.3%	7.6%
Frame Shift Insertion	3.3%	6.3%	4.2%	2.5%	4.6%	3.6%	3.8%	2.3%
In Frame Deletion	0.0%	0.0%	2.8%	0.0%	1.2%	0.8%	2.3%	1.4%
In Frame Insertion	0.0%	0.0%	2.8%	0.0%	0.6%	0.8%	0.5%	0.8%
Missense Mutation	63.9%	68.8%	54.2%	72.5%	64.4%	64.0%	65.9%	67.8%
Nonsense Mutation	19.7%	14.6%	15.3%	7.5%	16.0%	16.8%	15.3%	13.1%
Nonstop Mutation	0.0%	0.0%	0.0%	0.0%	0.0%	0.0%	0.1%	0.0%
Splice Region	0.0%	0.0%	0.0%	2.5%	0.3%	0.0%	0.1%	0.0%
Splice Site	6.6%	8.3%	5.6%	5.0%	4.6%	6.4%	5.5%	7.0%
**TRAF2**								
Frame Shift Deletion	100.0%	0.0%	0.0%	0.0%	20.0%	0.0%	10.0%	46.7%
Frame Shift Insertion	0.0%	0.0%	0.0%	0.0%	20.0%	0.0%	10.0%	6.7%
In Frame Deletion	0.0%	0.0%	0.0%	0.0%	0.0%	0.0%	10.0%	0.0%
Missense Mutation	0.0%	0.0%	0.0%	100.0%	60.0%	75.0%	60.0%	46.7%
Nonsense Mutation	0.0%	0.0%	0.0%	0.0%	0.0%	25.0%	0.0%	0.0%
Splice Site	0.0%	0.0%	0.0%	0.0%	0.0%	0.0%	10.0%	0.0%

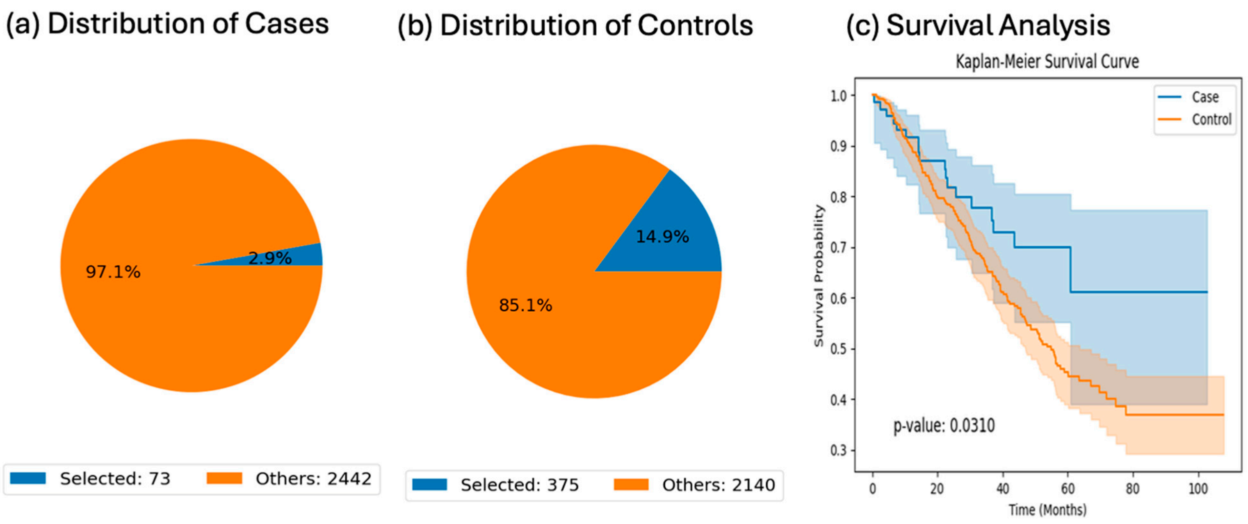

Figure S1. AI-directed cohort selection and survival comparison for early-onset treated Hispanic/Latino (H/L) and Non-Hispanic White (NHW) colorectal cancer (CRC) patients. This figure illustrates how the conversational AI platform identified matched case and control groups using user-defined clinical criteria and subsequently evaluated differences in overall survival (OS). (a) Within early-onset H/L CRC cases treated with FOLFOX, the AI tool isolated 73 eligible samples (2.9% of all available records), while the remaining 97.1% were not selected based on inclusion parameters. (b) Among early-onset NHW CRC controls treated with FOLFOX, 375 patients (14.9% of all available records) met the same selection rules, with 85.1% of records excluded. (c) Kaplan-Meier analysis visualizes OS differences between case and control cohorts, demonstrating a statistically significant survival distinction (log-rank p = 0.0310), with the case cohort displaying improved outcomes over time. Confidence intervals (95%) are shown for each curve, reflecting uncertainty around survival estimates. The figure highlights the ability of AI-driven cohort generation to support survival comparisons across demographic groups sharing matched clinical features.

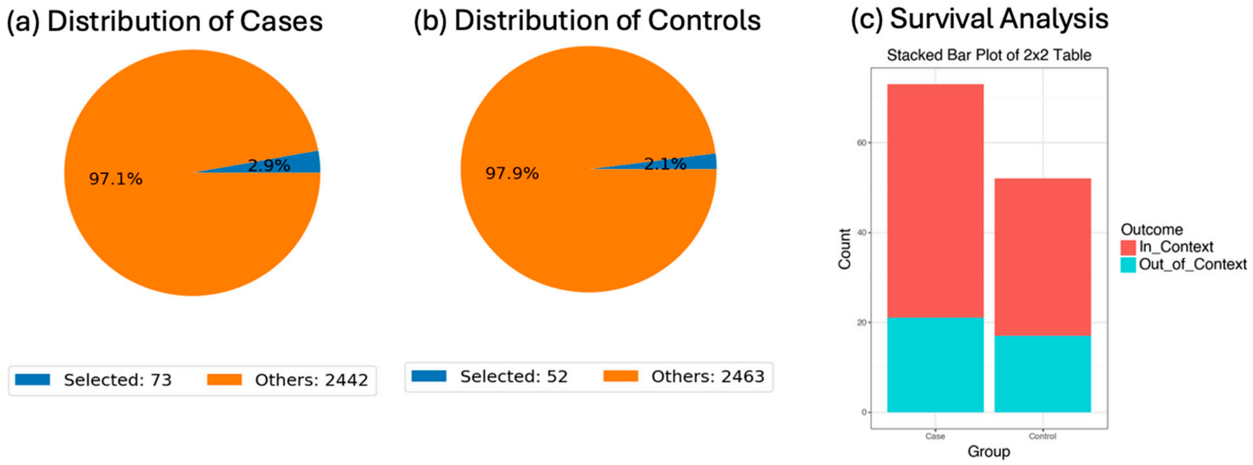

Figure S2. AI-driven analysis of MSI stability patterns among early-onset Hispanic/Latino colorectal cancer patients stratified by FOLFOX treatment exposure. This figure demonstrates how conversational querying was used to isolate and compare two subgroups within early-onset H/L colorectal cancer (CRC): individuals treated with FOLFOX (case cohort; n = 73) and those who did not receive FOLFOX (control cohort; n = 52). Panels (a) and (b) show the distribution of selected samples (blue) relative to the total available dataset (orange) for each subgroup, highlighting the proportion of patients meeting the defined criteria. Panel (c) presents a stacked bar visualization of a 2×2 odds ratio test evaluating whether microsatellite stability (MSI-stable genotype) differed between treatment groups. Fisher's exact test revealed no significant association between FOLFOX exposure and MSI stability status (p = 0.785), with similar proportions of MSI-stable tumors

observed in both groups. These findings suggest that, within early-onset H/L CRC, MSI stability patterns remain consistent regardless of FOLFOX treatment, and further demonstrate the utility of AI-guided cohort construction for efficient molecular comparison and validation across clinically defined strata.

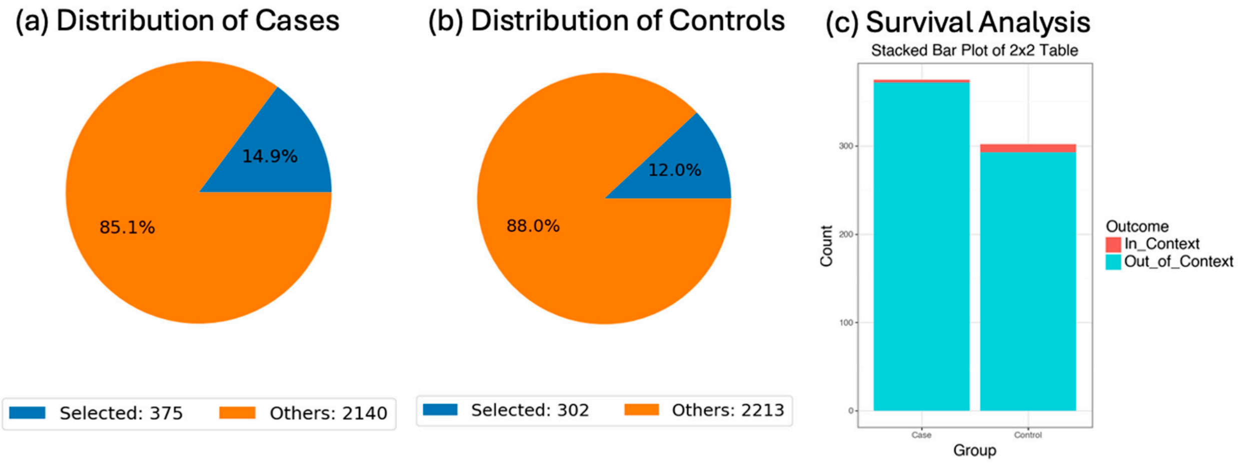

Figure S3. AI-guided assessment of AKT3 mutation frequency among early-onset Non-Hispanic White (NHW) colorectal cancer patients stratified by FOLFOX exposure. This figure illustrates how conversational filtering was used to compare AKT3 mutation prevalence between early-onset NHW CRC patients treated with FOLFOX (case cohort; n = 375) and those who did not receive FOLFOX (control cohort; n = 302). Panels (a) and (b) show pie charts representing the proportion of selected samples meeting the AKT3 mutation criterion ("in-context"; blue) versus all other samples ("out-of-context"; orange) within each subgroup. Panel (c) displays a stacked bar visualization of the odds ratio analysis, contrasting AKT3-mutated and non-mutated cases across treatment strata. Fisher's exact testing revealed no statistically significant association between FOLFOX exposure and AKT3 mutation status (p = 0.065), although mutation frequency was numerically lower in the treated cohort. These results suggest that AKT3 mutation prevalence remains relatively consistent regardless of chemotherapy exposure in early-onset NHW CRC and demonstrate the utility of AI-based cohort selection for rapid, treatment-specific genomic comparisons.

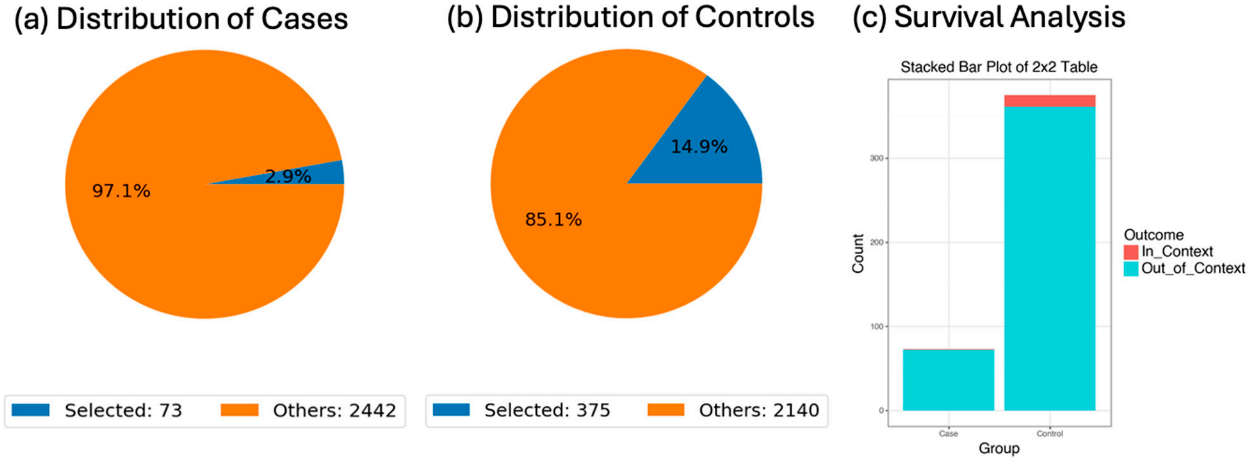

Figure S4. AI-supported evaluation of PDGFRB mutation prevalence in early-onset colorectal cancer patients treated with FOLFOX across Hispanic/Latino (H/L) and Non-Hispanic White (NHW) populations. This figure shows how the conversational AI framework was used to generate matched cohorts for comparing PDGFRB mutation rates between early-onset H/L CRC patients treated with FOLFOX (case cohort; n = 73) and early-onset NHW patients also treated with FOLFOX (control cohort; n = 375). Pie charts in panels (a) and (b) depict the proportion of cases meeting the

PDGFRB mutation criterion ("selected") relative to the total number of available samples within each subgroup. Panel (c) presents a stacked bar visualization of the odds ratio analysis, comparing the number of PDGFRB-mutated and non-mutated samples across the two ancestry groups. Fisher's exact testing did not reveal a statistically significant difference in PDGFRB mutation prevalence between treated H/L and NHW patients (p = 0.502), although mutation frequency was numerically lower in the H/L cohort. These findings suggest broadly comparable PDGFRB mutation rates across FOLFOX-treated early-onset patients irrespective of ancestry, and highlight the value of AI-driven cohort selection in enabling rapid, ancestry-aware molecular comparisons.

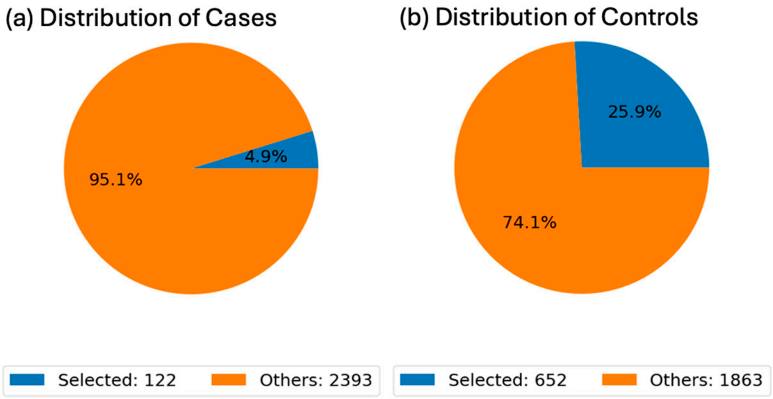

(c) Attribute Associations between case and control groups

Attribute	p-value
TP53_mutation_status	2.82E-51
APC_mutation_status	2.86E-50
KRAS_mutation_status	1.18E-14
TCF7L2_mutation_status	8.75E-06
PIK3CA_mutation_status	0.00011696
SMAD4_mutation_status	0.00032308
BRAF_mutation_status	0.0083243
PIK3R1_mutation_status	0.01838968
AMER1_mutation_status	0.03827215
SMAD3_mutation_status	0.04884447
NF1_mutation_status	0.05054189
CTNNB1_mutation_status	0.05285078
IGF1R_mutation_status	0.06172712
SMAD2_mutation_status	0.0632292
AXIN2_mutation_status	0.07071871
PTEN_mutation_status	0.07280087
ATR_mutation_status	0.08846251
ERBB3_mutation_status	0.08846251
ERBB2_mutation_status	0.09894139
ALK_mutation_status	0.10001999
TGFBR2_mutation_status	0.12378158

Figure S5. AI-enabled comparison of MAPK-altered early-onset colorectal cancer tumors reveals ancestry-linked molecular differences. This figure demonstrates how the conversational AI platform was used to evaluate mutation-level attributes distinguishing early-onset Hispanic/Latino (H/L) colorectal cancer (CRC) tumors from early-onset Non-Hispanic White (NHW) tumors, restricted to MAPK-altered samples. Pie charts in panels (a) and (b) display the proportion of selected (MAPK-altered) samples relative to all available cases within each ancestry group, showing a larger selected fraction among NHW patients (652/2,515; 25.9%) compared with H/L patients (122/2,515; 4.9%). Panel (c) summarizes mutation attributes that differed significantly between groups based on AI-driven statistical prioritization. The most strongly associated alterations included TP53, APC, KRAS, TCF7L2, and PIK3CA (all $p < 0.001$), followed by others such as SMAD4, BRAF, and NF1. The Figure highlight pronounced ancestry-associated molecular heterogeneity within MAPK-altered early-onset CRC and illustrate how an interactive AI framework can rapidly surface genomic features driving differences between clinical populations.
